# Internally promoted reactivity of carbonized butadiene and amines enables a recyclable polymer composite

**DOI:** 10.1126/sciadv.aed1295

**Published:** 2026-05-06

**Authors:** Keaton M. Turney, Abdol Hadi Mokarizadeh, Mesfin Tsige, James M. Eagan

**Affiliations:** School of Polymer Science and Polymer Engineering, The University of Akron, Akron, OH 44325-3909, USA.

## Abstract

The catalytic conversion of carbon dioxide into polymers via high-energy comonomers offers a sustainable, low-cost, and low-emission approach to developing conveniently manufactured high-performance materials without competing for land use or food resources. We present the synthesis of poly(amidoamine) polymers stoichiometrically derived from carbon dioxide, butadiene, and amines displaying useful mechanical properties (tensile strength, 43 MPa; Young’s modulus, 840 MPa; and flexural modulus, 2.6 GPa). The low viscosity precursors (20 centipoise at 25°C) are applicable to producing carbon fiber reinforced polymers with fiber wetting and rapid network formation (16 min at 150°C). This work reveals that the reactivity of the internal hydrogen bonding catalyzes the ring-opening polymerization, and the intramolecular alcohol moiety promotes chemical recyclability to the monomers under acidic conditions, allowing the carbon fibers to be recovered with <1.0 wt % difference and reused in the manufacturing of recycled composites.

## Teaser

Carbon dioxide is combined with abundant chemicals to produce recyclable polymers and carbon fiber composites.

## INTRODUCTION

**Fig. 1.**
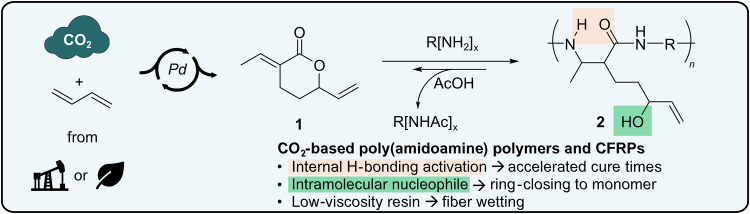
The catalytic carbonization of butadiene yields recyclable poly(amidoamines). The Pd-catalyzed coupling of CO_2_ with 1,3-butadiene as a path to bis-electrophile monomers for the self-catalyzed copolymerization with amines and acid-promoted depolymerization.

The combined utilization of biomass with carbon dioxide (CO_2_) to produce polymeric materials is critical to remaining within the environmental planetary boundaries for plastics (*1*). 1,3-Butadiene is a near-term carbon-negative chemical derived from low-cost fossil or bio-feedstocks (*2*–*5*) that can be catalytically and continuously converted into unsaturated lactone **1** through the Pd-catalyzed telomerization with CO_2_ (Fig. 1) (*6*–*10*). Lactone **1** exhibits a range of divergent polymerization mechanisms (*11*–*13*) to produce durable (*14*), reversible (*15*), or degradable (*16*) linkages and thermomechanical properties suitable for applications as elastomers (*17*), adhesives (*18*), and films (*19*, *20*).

Low-viscosity chemical precursors to high-strength materials are another vital technology for strategic anthropogenic decarbonization (*21*, *22*). When reinforced with agents such as carbon fiber, thermoset composites exhibit specific tensile strengths exceeding steel or aluminum counterparts (*23*). However, conventional thermosets are permanent polymer networks, which complicate the recovery of the energy-intensive carbon fiber as well as the polymer matrix. There continues to be a need for efficient methods for recovering intact carbon fibers and diverting carbon fiber reinforced polymer (CFRP) waste from landfills (*24*, *25*). Advances toward this goal have been made in cleaving epoxy/amine thermoset matrices (*26*–*28*), reprocessable dynamic networks (*29*–*34*), and bio-based composites (*32*, *35*–*38*). Relative to biomass feedstocks, however, CO_2_ utilization in preparing recyclable fiber-reinforced thermoset matrices is less developed (*39*). An example of this paradigm was reported by Detrembleur and co-workers in which reversible urethane (*40*) or dynamic N,S-acetal exchanges (*41*) were used to impart sustainability, performance, and recovery of the components.

**Fig. 2.**
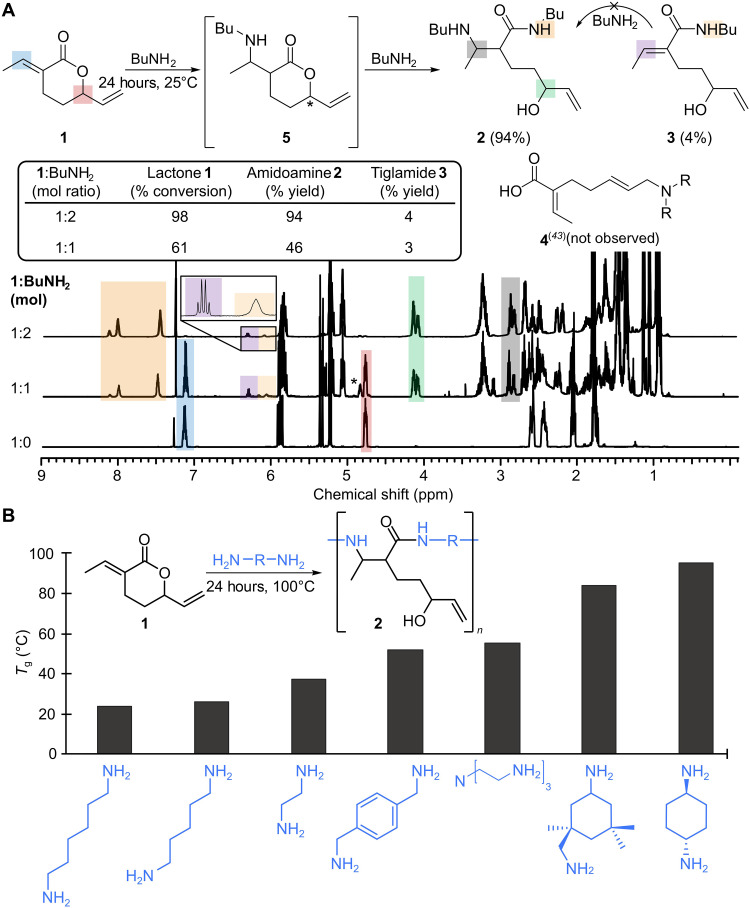
Lactone reactivity and the structure-property relationship between diamine monomer with resulting glass transition temperatures of poly(amidoamine) products. (**A**) ^1^H-NMR (500 MHz, CDCl_3_) analysis of products and intermediates for the amidoamination reaction between monofunctional *n*-butylamine and lactone **1**. (**B**) Glass transition temperatures of the poly(amidoamines) as measured by differential scanning calorimetry (DSC) using the inflection point of the second heating cycle at 10°C min^−1^.

The understanding of reactivity between **1** and amine hardeners is not fully elucidated. Nozaki and co-workers reported the postpolymerization amidation modification of polyacrylate polymers from **1** in the presence of stoichiometric strong base (*n*-BuLi) and showed the partial reversibility of the process at 260°C (*13*, *42*). Earlier, Behr and Henze described the metal-catalyzed amination of **1** with amines to yield small-molecule amino acids (e.g., **4**; Fig. 2A) (*43*–*45*). Given the broad use of multifunctional amines in the preparation of lightweight composites for decarbonization, we investigate here the catalyst-free amidation/amination reactivity of carbonized butadiene (i.e., lactone **1**) and its applications to recyclable CFRPs.

## RESULTS

In the absence of catalyst, lactone **1** undergoes conjugate 1,4-addition (i.e., *aza*-Michael) and ring-opening amidation to yield poly(amidoamine) small molecules, polymers, and thermosets from monofunctional, difunctional, and trifunctional amines, respectively (**2**; Fig. 2A). The 1,4-conjugate addition and ring-opening of **1** with amines is not entirely unexpected—acrylic esters and amines are used in the production of poly(amidoamine) dendrimers (*46*), and **1** is known to undergo conjugate and ring-opening polymerization (*11*)—however, amidoamines have not previously been observed from **1**. In the course of studying metal-catalyzed hydroamination, Behr and Henze proposed that lactone **1** reacts with amines to yield unsaturated amino acid **4** (Fig. 2A) via the metal π-allyl intermediate (*43*, *44*). Monosubstituted amines were investigated and several plausible products (table S1) were provided (*45*). In the absence of catalyst, we find that combining **1** and a monosubstituted primary amine (*n*-butylamine, BuNH_2_, 2 equiv) at 25°C for 24 hours in the absence of solvent yields dibutylamidoamine **2** in 94% yield.

The nuclear magnetic resonance (NMR) spectra of **2** are distinct from the amino acid moieties (e.g., **4**) at both the tiglate and allylic resonances (table S1). Compound **2** is absent of tiglate β-hydrogen and retains the monosubstituted alkene; connectivity of which was confirmed by a combination of two-dimensional (2D) NMR techniques (figs. S1 to S23). ^1^H-NMR of the reaction indicated full conversion of **1** to a mixture of diastereomers of compound **2** and notable resonances at 7.47, 8.02, and 8.12 parts per million (ppm) integrating to a single proton (Fig. 2A). These downfield signals are assigned to the diastereotopic intramolecular hydrogen bonding of the β-amidoamine moiety, and these resonances are quenched in the presence of D_2_O (fig. S4).

To ascertain the sequence of the 1,4 conjugate addition amination (i.e., *aza*-Michael) and ring-opening amidation reactions, *n*-butylamine was made the limiting reagent by subjecting lactone **1** to a single equivalent of amine. Over the course of 24 hours, in situ NMR observed the bis-addition product (**2**) and starting material in a near 1:1 ratio (Fig. 2A); the tiglamide coproduct (**3**) was also quantified in 3% yield. Aminolactone **5** was observed in 13% yield as evidenced by the minor lactone resonance at 4.9 ppm, but degrades during isolation. The low yield of monoaddition products at 1:1 stoichiometry (**1**:BuNH_2_) suggests that the proposed intermediates **3** and/or **5** are kinetically more reactive than the starting material is to the *aza*-Michael or ring-opening amidation, respectively. When tiglamide **3** was isolated from the reaction by column chromatography and resubjected to BuNH_2_ (up to 10 equiv, 25°C, 24 hours) no reaction was observed (fig. S7). This demonstrates that ring-opening of the unsaturated lactone is not the dominant pathway and supports the hypothesis that 1,4-addition precedes ring-opening via a saturated lactone moiety.

With this understanding of the chemical reactivity, we applied the amination/amidation sequence to producing polymeric products using diamine comonomers in a step-growth addition polymerization. First, the reaction conditions were optimized using 1,6-hexanediamine in equimolar ratio to **1**, achieving full conversion by ^1^H-NMR within 3 hours, and a maximum molecular weight (5640 g/mol) within 24 hours at 100°C (table S2). Matrix-assisted laser desorption/ionization mass spectrometry (MALDI-MS) was used to investigate the repeat structure and end groups of the poly(amidoamine) product. The MALDI-MS of the **1**/1,6-hexanediamine copolymer (figs. S24 to S55) showed a distribution of polymeric species differing by 268 mass/charge ratio, consistent with an A_2_/B_2_ step-growth polymerization. This precludes the bis-alkylation reaction of amine moieties (*46*), an observation that is attributed to the internal H-bond stabilizing the β-amine functionality from further alkylation. Diffusion-ordered NMR spectroscopy (DOSY) revealed that the minor tiglamide resonance, observed in the model studies, was chemically incorporated into the polymers and contributes to the termination mechanism (fig. S30). The tiglamide end-group concentration increases with temperature, up to 20% at 150°C (fig. S93), beyond which radical cross-linking began to take place.

Toward the aim of synthesizing high-strength polymers, we observed higher polymer glass transition temperatures (*T*_g_s) with rigid amine comonomers (Fig. 2B). The length of the alkyl diamine was inversely related to the *T*_g_ such that hexanediamine, bio-based pentanediamine, and ethylenediamine afforded polyamidoamines with *T*_g_s near room temperature (24°, 26°, and 37°C, respectively). More rigid aromatic *p*-xylylenediamine increased the *T*_g_ to 52°C, while the saturated *trans*-1,4-diaminocyclohexane afforded the highest *T*_g_ of 95°C. Amine hardeners such as tris(2-aminoethyl)amine (TREN) and isophoronediamine also produced high *T*_g_ poly(amidoamines) of 55° and 84°C, respectively. Other comonomers such as aminotelechelic oligoethers yielded *T*_g_s as low as −30°C (table S3).

**Fig. 3.**
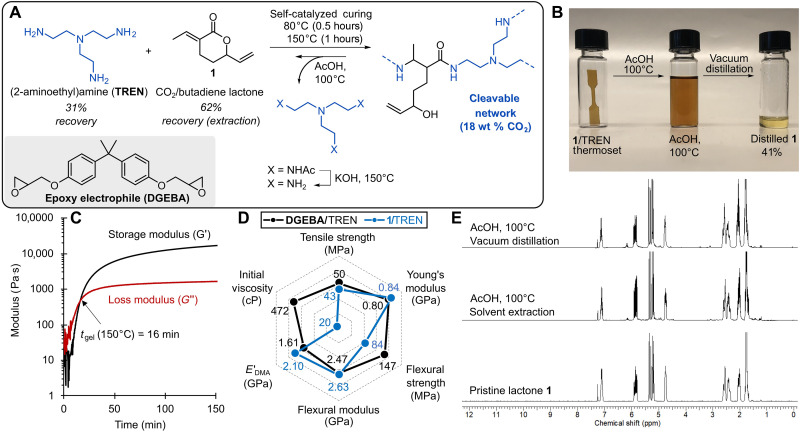
Synthesis, properties, and recycling of poly(amidoamine) networks derived from butadiene, CO_2_, and amine hardener. (**A**) The synthesis of baseline epoxy/amine resins compared to recyclable amidoamine resins. (**B**) Optical image of test specimen and stages of recycling. (**C**) SAOS rheometry of the liquid resin at 150°C. (**D**) Spider plot comparing performance of epoxy/amine and poly(amidoamine) resins. (**E**) ^1^H-NMR of virgin lactone **1** and recovered lactone **1** from organic extraction or vacuum distillation after acetic acid depolymerization.

Motivated by the use of multifunctional electrophiles such as diglycidyl ether bisphenol-A (DGEBA; Fig. 3A) to make high-strength, rigid materials, we studied the bis-electrophilic behavior of **1** paired with TREN to synthesize poly(amidoamine) networks. Mixtures of **1**/TREN (3:2 mol ratio) solidify at room temperature into insoluble thermosets over 24 hours without any added catalyst. Alternatively, by increasing the reaction temperature to 150°C, we found that the cure time could be decreased to 1.5 hours to yield a translucent orange poly(amidoamine) network with a *T*_g_ of 55°C (Fig. 3B). We observed the transition from a viscous liquid to an elastic solid (*G*′ > *G*″) within 16 min, as measured by small amplitude oscillatory shear (SAOS) rheology (Fig. 3C). The viscosity of **1**/TREN [20 centipoise (cP) at 25°C] is notably lower than the DGEBA/TREN (472 cP) by rotary viscometer, and the time to viscosity doubling was 3 and 37 min, respectively (fig. S59). This catalyst-free reactivity accelerates manufacturing throughput with less energy and is attributed to the internal H-bond catalyzing the amidation reaction.

Mechanical test specimens were prepared in a two-stage process of first heating at 80°C for 30 min, followed by heating at 150°C for 1 hour. This procedure proved optimal for minimizing sample defects in test specimens. Shown in Fig. 3D are the mechanical properties of the resulting networks, which rivaled amine-cured DGEBA epoxy permanent thermosets. Both the poly(amidoamine) and epoxy reference materials have high ultimate tensile strengths (43 ± 4 and 50 ± 2 MPa, respectively), Young’s moduli (840 ± 41 and 796 ± 72 MPa), and flexural moduli (2.6 ± 0.2 and 2.5 ± 0.1 GPa).

To understand the network’s stability, a chemical-resistance study was conducted (figs. S61 and S62). In the presence of solvents [acetone, *N*,*N*′-dimethylformamide (DMF), MeCN, MeOH, and H_2_O] and under basic conditions (1 M NaOH), the gel fractions remained above 90% after 24 hours of submersing the specimens, indicating that the network had not lost percolation connectivity. However, when heated to 100°C in acidic environments (i.e., AcOH), the gel fraction decreased to 0% over 24 hours (table S4). After exposure to acid, it was anticipated that the amide moiety would be stable and that the retro-*aza*-Michael elimination would occur to produce a mixture of mono-, di-, and tri-tiglamide products; however, these species were not observed spectroscopically. Instead, the major products observed by ^1^H-NMR was lactone **1** (Fig. 3E). Although reversible lactonization of the amide moiety by the intramolecular cyclization of the alcohol is considered thermodynamically unfavored in the reaction equilibrium, when heated to 100°C, even trace reversion to the monomer **1** is trapped due to the free amine’s nucleophilicity being sequestered, thereby shifting the equilibrium process and funneling the reaction products to starting lactone **1** (Fig. 3A). Unlike condensation polymerizations (*46*), the departing alkoxy remains covalently attached in the products from lactone **1**, which imparts atom economy and mild chemical recyclability in these thermosets. Analysis by ^1^H-NMR showed reversion to lactone **1** in an observed yield of 72%, which could be experimentally recovered by simple organic extraction with a 20:80 ethyl acetate:hexanes organic phase (62% isolated yield), yielding **1** in a 25:1 ratio of *E*/*Z* isomers, consistent with a retro-*thia*-Michael sequence reported by Zhang *et al.* (figs. S63 to S68) (*47*). Alternatively, direct distillation was also suitable for chemical recycling of monomer **1** (41% isolated yield), and the unsaturated lactone was isolated in a 10:1 ratio of *E*/*Z* isomers with this procedure, with the amine recovered from the pitching flask as TREN-trisacetate (figs. S95 and S96). The TREN-trisacetate was hydrolyzed with KOH at 150°C to afford TREN in a 31% overall yield. Notably, the crude solution NMR analysis indicates good yields and purity, despite the overall low recovery, such that we attribute material losses to intractable cross-linking side reactions that result from the divergent reactivity of **1** and its derivatives (*12*). When the repurified monomers were resubjected to the reaction conditions, thermosets were obtained with comparable uniaxial tensile properties with ultimate tensile strengths of 41 ± 5 MPa and Young’s modulus of 788 ± 54 MPa (figs. S97), indicating that the network integrity was not degraded by the *E*/*Z* isomerism of **1**.

**Fig. 4.**
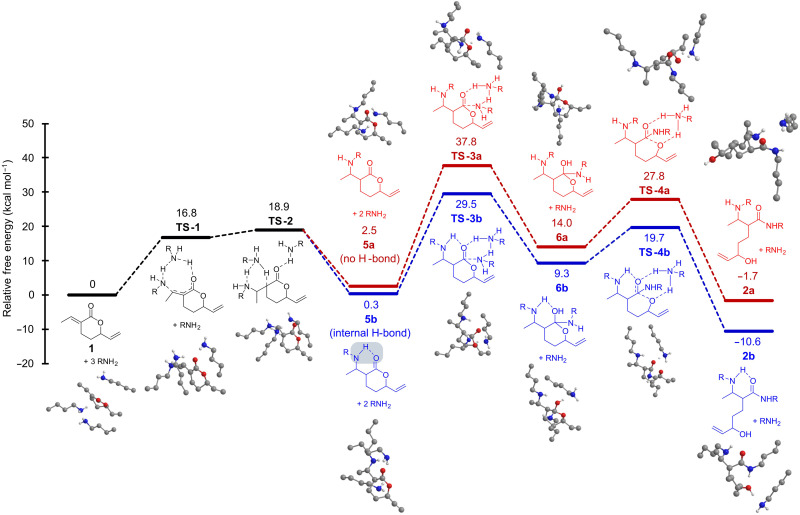
Energy diagram of amidoamination pathways and the impact of internal H-bonding on the relative free-energies. Values Δ*G* (kcal mol^−1^) are relative to the starting lactone **1** and three equivalents of *n*-butylamine (R = nBu) according to DFT calculations with a B3LYP/6-311G++(d,p) functional/basis set. Transition states were found for all transformations, geometrically optimized, and confirmed by the intrinsic reaction coordinates. The reaction pathway without internal H-bonding (a) is shown in red, while the H-bonded pathway (b) is shown in blue. Nonpolar hydrogen atoms are omitted for clarity.

The mechanistic origins of the fast cure times and cleavability were next investigated using density functional theory (DFT) calculations. Complementing the experimental spectroscopic and reactivity data, computational insights provide quantitative values to the lowest energy pathway and prove the importance of the internal H-bonding catalysis. In a prior DFT calculation of *aza*-Michael reactions (*48*), the lowest barrier pathways were observed for amine-assisted (i.e., two amines involved in proton transfer) and stepwise conjugate addition (i.e., enol formation and tautomerization) mechanisms. An analogous amine-assisted proton-shuttling pathway has been modeled for the amidation of formate esters to aid the collapse of the tetrahedral hemi-carbamate intermediate (*49*). Figure 4 presents the results of this approach for the amidoamination of lactone **1** using *n*-butylamine with (blue) and without (red) internal H-bond activation.

The initial *aza*-Michael reaction proceeds through an amine-assisted 1,4-addition (**TS-1**) and proton transfer (**TS-2**). The free-energy barrier of the assisted pathway (Δ*G*^‡^ = 18.9 kcal mol^−1^) was lower than the concerted pathway (Δ*G*^‡^ = 42.8 kcal mol^−1^; figs. S15 and S16) (*48*). The resulting aminolactone intermediates **5a** (Δ*G* = 2.5 kcal mol^−1^) and **5b** (Δ*G* = 0.3 kcal mol^−1^) differ by their ability to internally hydrogen-bond between the β-amine and lactone carbonyl. In addition to stabilizing the intermediate, the H-bond pathway lowers the activation energy for nucleophilic amidation. The H-bonded transition state (Δ*G*^‡^ = **TS-3b**, 29.5 kcal mol^−1^) provides an activation barrier (ΔΔ*G*^‡^) that is 6.1 kcal mol^−1^ lower than the **TS-3a** pathway, which lacks internal hydrogen bonding. The resulting hemi-carbamate intermediate **6b** (Δ*G* = 9.3 kcal mol^−1^) is also stabilized by the β-NH interaction, and the ring-opening transition state (**TS-4b**) was 8.1 kcal mol^−1^ less than the uncatalyzed pathway (**TS-4a**). The entire amidoamination reaction was exergonic with the internal H-bond interaction producing the more stable **2b** (Δ*G*° = −10.6 kcal mol^−1^) relative to **2a** (Δ*G*° = −1.7 kcal mol^−1^). As a result, the internal H-bond both stabilizes the reaction intermediates and products, and lower the activation energies in the self-catalyzed amidoamination reaction. The effects related to H-bond stabilization of the poly(amidoamines) are pronounced during dynamic mechanical analysis (DMA). The storage moduli of the **1**/TREN and DGEBA/TREN networks were subjected to temperature ramp experiments, and their molecular weights between cross-links were calculated (fig. S98). Despite **1**/TREN showing a higher modulus below *T*_g_ (2.1 ± 0.5 GPa) than the DGEBA/TREN network (1.7 ± 0.1 GPa), the material shows a large drop in storage modulus at elevated temperatures. Greater molecular weight between cross-links is observed in the **1**/TREN (*M*_c_ = 10.3 ± 0.6 kDa) compared to DGEBA/TREN (*M*_c_ = 2.3 ± 0.1 kDa). We suggest that this is related to the cyclic internal hydrogen bonds in the poly(amidoamines) that generates a more rigid polymer backbone, improving modulus at lower temperatures. As the H-bond interactions are thermally disrupted, the segmental motion increases above the poylmer’s *T*_g_, showing a drop in modulus and increased *M*_c_.

**Fig. 5.**
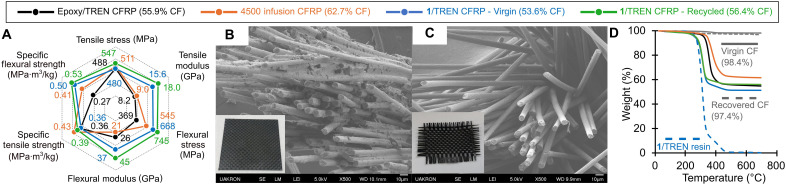
Properties and recyclability of poly(amidoamine) carbon fiber composites. (**A**) Spider plot comparing the performance of the poly(amidoamine) and epoxy CFRPs. (**B**) SEM image of fractured poly(amidoamine) CFRP fiber bundles with inlayed optical image of full panel. (**C**) SEM images of the recovered fibers from acetic acid digestion (100°C, 1 hour) with inlaid optical image. (**D**) TGA of poly(amidoamine) resin, CFRPs, and carbon fibers.

The low viscosity, short cure times, tensile properties, and chemical recyclability of these 18 wt % CO_2_ networks were identified as being particularly useful in preparing CFRPs with recoverable fibers (Fig. 5). CFRPs using the **1**/TREN, DGEBA/TREN, and commercial epoxy resins (Fibre Glast 4500) were prepared by a hand-layup procedure of resin with four plies of 3K continuous plain weave carbon fiber. This prepreg was cured using a low-temperature precure at 80°C for 30 min followed by a high-temperature curing period at 150°C for 1 hour. The poly(amidoamine) CFRP exhibited tensile strength, Young’s modulus, flexural strength, and flexural modulus properties (σ_break_ = 480 ± 46 MPa, *E*_Young’s_ = 15.6 ± 2.8 GPa, σ_flexural_ = 668 ± 111 MPa, and *E*_flexural_ = 37 ± 7.0 GPa) that were comparable or superior to those of the DGEBA/TREN CFRP control samples as well as the commercial epoxy resin in ambient environments, indicating that the resin may be competitive in certain applications. The density calculations revealed the same trends in specific strengths (table S5). The relative higher modulus and flexural stress values in **1**/TREN are proposed to arise from the lower viscosity and ability for the poly(amidoamine) moieties to transfer stress between the matrix and fibers. This is evidenced by flexural specimens exhibiting a stress-induced failure in **1**/TREN, but a compressive failure in the epoxy-based resins. By thermogravimetric analysis (TGA), the carbon fiber content of the four-ply composites was found to be composed of 54, 56, and 63 wt % carbon fiber for the poly(amidoamine) and DGEBA/amine, and commercial epoxy CFRPs, respectively (Fig. 5D).

Scanning electron microscopy (SEM) of the cross-section of a fractured poly(amidoamine) CFRP specimen revealed that the polymer penetrates between fibers, which is facilitated by the low initial viscosity (20 cP at 25°C) of the **1**/TREN resin (Fig. 5B).The cleavable network enabled both the monomer and the carbon fiber fabrics to be recovered via degradation of the rigid composite in acetic acid at 100°C for 1 hour (Fig. 5C and movie S1). To evaluate the efficiency of matrix removal, the flexible fiber weave was removed from the acid, rinsed with water, dried in a vacuum oven at 60°C, and analyzed by SEM (Fig. 5C). The images showed no visual evidence of residual resin on the fibers, which was corroborated by TGA, which demonstrated a <1.0 wt % difference between the virgin and recovered fibers at 500°C (Fig. 5D) (*28*). With the recovered carbon fiber, we tested the performance of a recycled CFRP by refabricating a **1**/TREN panel with the visually pristine fibers. This recycled CFRP, consisting of 56 wt % carbon fiber, exhibited no loss in mechanical properties (Fig. 5A), suggesting that the mild digestion conditions cause negligible damage to the fiber surface when used with the poly(amidoamine) resin.

## DISCUSSION

In conclusion, the carbonization of butadiene with CO_2_ yields lactone **1**, which reacts with amine hardeners to afford poly(amidoamine) materials through an internal H-bond promoted tandem *aza*-Michael/ring-opening reaction (*50*). The structure of the amine determines the *T*_g_ (−30° to 95°C) and connectivity of the polymers, which can yield thermoset networks (σ = 43 MPa, *E*_Young’s_ = 0.8 GPa, and *E*_flex_ = 2.6 GPa) and CFRPs (σ = 480 MPa, *E*_Young’s_ = 15.6 GPa, σ_flexural_ = 668 MPa, and *E*_flex_ = 39.0 GPa). The low viscosity of the resin system (20 cP at 25°C) assists with fiber wetting, while the self-catalyzed network formation results in gel times of 16 min. It was further shown that in the presence of hot acetic acid, the carbon fibers and monomer could be recovered with high purities by the combination of an internal hydroxy nucleophile and shifting of the amidation/lactonization equilibrium, and the fibers were suitable for reuse with no observed loss in performance. These materials introduce reactivity for thermoset cleavability, which should be selected based on the composite’s use-time and environment; many long-term structural applications will still benefit from more durable thermosets. Careful life cycle assessment of CO_2_ utilization and end-of-life scenarios is also necessary to quantify the benefits of these flexible feedstocks relative to emerging bio-based paradigms. It is shown that through the synergistic utilization of CO_2_ with low-cost near-term biochemicals, the described poly(amidoamines) reactivity simultaneously addresses sustainable function, manufacturability, thermoset composite recycling, and low-carbon feedstocks.

## MATERIALS AND METHODS

### Materials

Carbon dioxide (99.5%) was purchased from Linde Gas and Equipment Company, and stabilized 1,3-butadiene (99%) was purchased from ChemSampCo. Fibre Glast 4500 infusion resin and 696-A Unfinished Edge Carbon Fiber Tape (6 inches by 10 yards) were purchased from Fibre Glast. All amines and other reagents were purchased from sources identified in the Supplementary Materials and used as received, unless otherwise noted.

### Methods

High-high pressure reactions were performed in 300-ml stainless steel Parr reactor with a pressure release valve. NMR data were collected using 5-mm outer diameter tubes at 30.0°C. ^1^H-NMR spectra were recorded using a Varian Mercury (300 MHz) spectrometer or a Varian Inova (500 MHz) spectrometer and were referenced to residual nondeuterated solvent shifts (CHCl_3_, δ = 7.26 ppm or C_2_HD_5_OS, δ = 2.50) (^1^H). ^13^C-NMR spectra of polymers were recorded on Varian Inova (500 MHz) spectrometer and were referenced versus solvent shifts (CDCl_3_, δ = 77.16 ppm or C_2_D_6_OS, δ = 39.52). Internal integration standards were used where noted. The 2D NMR spectra for correlation spectroscopy and heteronuclear single quantum correlation were conducted using a Varian Inova (500 MHz) spectrometer, and DOSY was performed using a Varian Inova (750 MHz) spectrometer; all were referenced to the residual nondeuterated solvent resonance. All NMR data were processed and analyzed using ACD/NMR Processor Academic Edition software apart from DOSY analysis being conducted using MestreNova 15.0.1.

Molecular weights (*M*_n_ and *M*_w_) and molecular weight distributions (*Ð* = *M*_w_/*M*_n_) were determined by gel permeation chromatography (GPC). Analyses were performed using a Tosoh EcoSEC HLC-8320 GPC equipped with a refractive index (RI) detector. Columns consisted of one guard column [6.0 mm inner diameter (ID) by 4 cm, 7 μm], two TSKgel G4000H HR sample columns (7.8 mm ID by 30 cm, 5 μm), and a TSKgel SuperH-RC reference column (6.0 mm by 15 cm by 4 μm). High-performance liquid chromatography–grade DMF (10 mM LiBr) was used as an eluent at a flow rate of 0.5 ml/min at 40°C. Data were measured relative to polystyrene standards (*Ð* < 1.1)

*T*_g_s were measured by differential scanning calorimetry (DSC) using a DSC-TA Discovery DSC 250. Analyses were performed in hermetic aluminum pans under a nitrogen atmosphere with a gas flow rate of 40.00 ml/min. Data were collected using ~5 mg of material from the second heating cycle at a heating rate of 10°C/min between −40° and 120°C. Polymer *T*_g_ was analyzed using TA TRIOS software reporting the inflection point analysis value. Polymer degradation temperatures (*T*_d_s) were measured by TGA using a TA Instruments Q50 TGA under a nitrogen environment with a gas flow rate of 40.00 ml/min. Temperature ramp rate experiments were performed at a ramp rate of 10°C/min on ~10 mg of material. Polymer *T*_d_ was analyzed using TA Universal Analysis software and determined by the point of 5% weight loss. Composite weight percent carbon fiber was analyzed using TA Universal Analysis software and determined at 500°C following thermal degradation of the matrix (*28*).

Uniaxial tensile testing and flexural testing was performed on an Instron 5567 Universal Test Frame. Tensile testing of matrices was performed in accordance with ASTM D638. Tensile testing of composite samples was performed in accordance with ASTM D3039. Flexural testing was performed in accordance with ASTM D790. Data were recorded using Bluehill Universal software. Reported flexural moduli correspond to the tangent modulus calculated at 1% strain. SAOS was performed using a TA Discovery Hybrid Rheometer (DHR-2) equipped with an 8-plate with samples ≥1000 μm in thickness, and the data acquired at 1.0% strain and 10 rad/s at 150°C were recorded using TA TRIOS software. DMA was performed using a TA Dynamic Mechanical Analyzer Q800 equipped with a gas cooling accessory supplied with liquid nitrogen. The DMA was set up for single cantilever analysis and the experiments were designed and conducted using TA Advantage software. A frequency sweep was conducted on unreinforced resins to identify the linear viscoelastic region (LVE). Temperature ramps were performed at a single frequency (1.00 Hz) at an amplitude of 20 μm. Ramp procedures started with a 5-min soak at the starting temperature (−60°C), followed by a 3°C temperature ramp up to 180°C. Data were processed using TA Universal Analysis. Molecular weight between cross-links (*M*_c_) was calculated using the following equation

$$M_{c}=\frac{\mathrm{RTd}}{{E^{\prime }}_{\text{rubbery}}}$$

Electrospray ionization mass spectrometry (ESI-MS) was carried out on a Waters Synapt HDMS 1 quadrupole/time-of-flight (Q/ToF) mass spectrometer equipped with an ESI source. Stock solutions of the samples were prepared in acetonitrile at a concentration of 10 mg/ml and filtered using Acrodisc 0.2-μm pore size polyvinylidene difluoride filters. The filtered samples were then diluted in acetonitrile to a concentration of 100 ng/ml before being introduced to the ESI source via direct infusion at a flow rate of 35 μl/min. For ESI-MS measurements, the instrument was calibrated using a NaI standard and operated in positive ion mode under the following conditions: capillary voltage of 2.8 kV, an extraction cone voltage of 2.6 V, sampling cone voltage of 40.0 V, desolvation gas flow rate of 400.0 liter/hour (N_2_), trap cell collision energy (CE) of 4.0 eV, transfer cell CE of 4.0 eV, a source temperature of 100°C, and a desolvation temperature of 350°C. MALDI-MS experiments were performed on a Bruker Ultraflex III MALDI ToF/ToF mass spectrometer equipped with an yttrium-aluminum-garnet–Nd laser emitting at 355 nm. Stock solutions of 2-[(2*E*)-3-(4-tert-butylphenyl)-2-methylprop-2-enylidene]malononitrile matrix (99.0%, MilliporeSigma) and NaTFA cationization salt (98%, Aldrich Chemistry) were prepared in methanol at concentrations of 20 and 10 mg/ml, respectively. These solutions were then mixed in a volume ratio of 10:1 before spotting ~0.5 μl onto the target plate. Samples were dissolved in methanol at a concentration of 10 mg/ml and were spotted onto the target plate using the sandwich method. The instrument was calibrated using 2000 Da polymethyl methacrylate standard (Aldrich Chemistry) prior to sample analysis. The MALDI-MS data were analyzed using Bruker’s UltrafleXtreme v3.4. software. Molecular weights were obtained using Polymerix software.

SEM imaging was carried out on a JEOL JSM-7401F Field Emission SEM. Samples were adhered to an aluminum billet using carbon tape and sputter-coated with gold for 45 s under vacuum. Thermoset network and CFRP densities were measured using a Mettler Toledo density kit for precision balances. Viscosity was measured using an IKA Rotovisc Lo-Vi rotary viscometer. Resin systems were analyzed as a function of time using a low-volume 2.1-ml spindle and chamber accessory. Temperatures were controlled during analysis by water circulation. Environmental exposure analysis was performed with the use of a Cincinnati Sub-Zero ZP-16 environmental chamber.

### Synthesis of lactone 1

Lactone **1** was synthesized in a similar procedure to previous literature (*51*–*53*). A 300-ml stainless steel Parr reactor was charged with hydroquinone (2.82 mmol, 0.311 g), tris(4-methoxyphenyl)phosphine (0.62 mmol, 0.219 g), and palladium tris(dibenzylideneacetone)dipalladium(0) (0.07 mmol, 0.064 g) and purged with nitrogen. Acetonitrile (20 ml) was added to the vessel via syringe followed by a 250-ml stainless steel canister containing condensed butadiene (1.52 mol, 82.0 g) injected, followed by carbon dioxide (30 atm). The reactor was moved to a 70°C oil bath and stirred for 16 hours. The vessel was cooled and disassembled, and the solution was filtered through celite into a round-bottom flask and concentrated in vacuo. The resulting crude material (60.5 g, ~53% conversion) was distilled at 80°C under <200 mtorr of vacuum to yield pure lactone **1** (34.5 g). The following were exhibited: ^1^H-NMR (500 MHz, CDCl_3_): δ 7.13 (qt, *J* = 7.3, 2.3 Hz, 1H), 5.88 (ddd, *J* = 17.4, 10.5, 5.4 Hz, 1H), 5.35 (dt, *J* = 17.2, 1.4 Hz, 1H), 5.23 (dt, *J* = 10.5, 1.3 Hz, 1H), 4.77 (dddt, *J* = 9.4, 5.4, 2.9, 1.5 Hz, 1H), 2.55–2.63 (m, 1H), 2.39–2.48 (m, 1H), 2.05 (dtd, *J* = 13.7, 5.4, 2.9 Hz, 1H), 1.78 (dt, *J* = 7.3, 1.5 Hz, 3H), and 1.76 (m, 1H); ^13^C NMR (500 MHz, CDCl_3_): δ = 166.14, 141.04, 135.79, 125.91, 116.82, 78.84, 27.58, 21.92, and 14.01; Fourier transform infrared (FTIR) with attenuated total reflectance (ATR): ν = 2924, 2905, 1709, 1635, 1436, 1365, 1317, 1253, 1207, 1145, 1061, 989, 955, 924, and 721 cm^−1^.

For the reaction of lactone **1** with *n*-butylamine, 3-ethyliden-6-hepten-5-olide (**1**) (3.2 mmol, 0.486 g) and *n*-butylamine (6.4 mmol, 0.468 g) were added to a 20-ml scintillation vial and stirred at 25°C for 24 hours. The yellow viscous material was purified via flash-column chromatography using a 20:75:5 ethyl acetate:hexanes:triethylamine mobile phase to afford the clear yellow, viscous mixture of diastereomers of dibutylamidoamine **2** (0.881 g, 94% yield) and tiglamide **3** as a viscous orange material (0.021 g, 2% yield). The following were exhibited for dibutylamidoamine (**2**); {*N*-butyl-2-[1-(butylamino)ethyl]-5-hydroxyhept-6-enamide}: ^1^H-NMR (500 MHz, CDCl_3_): δ 8.12, 8.02, 7.47 (3 br s, 1H), 5.85 (m, 1H), 5.22 (m, 1H), 5.07 (m, 1H), 4.09 (m, 1H), 3.20 (m, 2H), 2.82 (m, 1H), 2.58 (m, 2H), 2.21 (m, 1H), 1.84 (m, 1H), 1.70 (m, 1H), 1.59 (m, 2H), 1.44 (m, 4H), 1.34 (dt, *J* = 15.0, 7.4 Hz, 4H), 1.06 (m, 3H), and 0.92 (m, 6H); ^13^C-NMR (500 MHz, CDCl_3_): δ 174.73, 174.26, 141.27, 141.07, 114.29, 114.07, 113.92, 72.78, 72.46, 72.24, 56.10, 55.52, 54.21, 52.75, 48.83, 47.25, 46.98, 38.77, 38.65, 35.98, 35.42, 32.39, 32.28, 31.66, 26.30, 25.42, 23.67, 20.45, 20.21, 18.15, 16.90, 13.89, and 13.67; high-resolution mass spectrometry (HRMS) (ESI-MS): exact mass [M] calculated for C_17_H_34_N_2_O_2_, 298.26; [M + H]^+^ detected at 299.285; FTIR (ATR): *n* = 3291, 3079, 2959, 2931, 2874, 1638, 1550, 1458, 1379, 1226, 1144, 1068, 991, 918, 735, and 689 cm^−1^. For tiglamide (**3**); [(*E*)-*N*-butyl-2-ethylidene-5-hydroxyhept-6-enamide]: ^1^H-NMR (500 MHz, CDCl_3_): δ 6.32 (q, *J* = 6.8 Hz, 1H), 6.10 (s, 1H), 5.88 (ddd, *J* = 17.1, 10.5, 5.6 Hz, 1H), 5.24 (dt, *J* = 17.1, 1.5 Hz, 1H), 5.08 (dt, *J* = 10.8, 1.5 Hz, 1H), 4.09 (ddd, *J* = 9, 5.4, 4.2 Hz, 1H), 3.29 (m, 2H), 2.42 (m, 2H), 1.76 (d, *J* = 7.3 Hz, 3H), 1.62 (m, 2H), 1.50 (quin, *J* = 7.5 Hz, 2H), 1.35 (dq, *J* = 14.9, 7.4 Hz, 2H), 1.25 (s, 1H), and 0.92 (t, *J* = 7.3, 3H); ^13^C-NMR (500 MHz, CDCl_3_): δ 170.07, 140.89, 136.98, 130.37, 114.23, 71.27, 39.53, 35.95, 31.58, 22.41, 20.10, 13.84, and 13.71; HRMS (ESI-MS): exact mass [M] calculated for C_13_H_23_NO_2_, 225.17; [M + Na]^+^ detected at 248.141; FTIR (ATR): *n* = 3309, 3082, 2961, 2927, 2875, 1659, 1616, 1537, 1439, 1382, 1308, 1227, 1121, 1072, 992, and 920 cm^−1^.

### General polymerization procedure

To a 20-ml scintillation vial, diamine (2 mmol) was added, followed by lactone **1** (2 mmol, 0.304 g). The vial was heated at 100°C with stirring for 24 hours, resulting in a visible increase in viscosity. The material was cooled, dissolved in 1 ml of methanol, and precipitated by dropwise addition into Et_2_O (20 ml). The suspension was decanted, and the precipitate was rinsed three times with 1 ml of Et_2_O, dried in a vacuum oven for 18 hours, and characterized as described in the Supplementary Materials.

### Synthesis of 1/TREN thermoset network

To a 20-ml scintillation vial, TREN (6.6 mmol, 0.962 g) was added, followed by lactone **1** (9.9 mmol, 1.50 g), and the resulting mixture was gently stirred with a wooden stick to avoid aerating the low-viscosity mix. The material was then transferred to a polytetrafluoroethylene (PTFE) mold using a syringe, heated in an oven at 80°C for 30 min, and then transferred a 150°C oven for an additional 60 min. The mold was cooled and the specimen demolded to afford a yellow rigid material. FTIR (ATR): *n* = 3891, 3073, 2918, 2850, 1699, 1636, 1577, 1540, 1472, 1433, 1387, 1261, 1110, 1046, 988, 915, 793, 719, and 668 cm^−1^.

### Synthesis of DGEBA/TREN epoxy networks

DGEBA was gently heated to 45°C and transferred to an 240-ml plastic mixing cup as a colorless, opaque, and highly viscous material (5 mmol, 1.703 g). Next, TREN (3.34 mmol, 0.488 g) was transferred to the mixing cup and gently stirred using a wood mixing stick in a manner avoiding aeration of the mixture. The mixture was transferred to the PTFE mold, placed in an oven at 80°C for 30 min, and then transferred to a 150°C oven for an additional 60 min. After the reaction, the mold was allowed to cool yielding a pale yellow, opaque, rigid material. FTIR (ATR): *n* = 3304, 3034, 2957 2919, 2850, 1654, 1607, 1508, 1456, 1360, 1292, 1237, 1179, 1102, 1026, 934, 846, 825, 752, and 729 cm^−1^.

### Preparation of CFRPs

Four sheets of Fibre Glast 696-A 6-inch unfinished edge plain biaxial weave carbon fiber tape (17.5 to 20.0 g) were cut into rectangles ~15 cm by 10 cm. The mass of the carbon fiber was adjusted based on the measurement of the resin formulations to target a composite panel of 50 wt % carbon fiber. Respective resin formulations (17.5 to 20.0 g) were prepared in an 240-ml plastic cup, and a small amount of resin was applied to PTFE film and spread to the cover the bottom of the initial sheet of carbon fiber. One layer of carbon fiber was laid on the film, and additional resin was poured on top, then a flexible plastic blade was used to spread the resin and penetrate the resin into the weave pattern of the carbon fiber. This process was repeated, matching the direction weave pattern to the previous layer, until four-ply prepreg was reached. The prepreg was transferred on the PTFE sheet into the oven where it was cured. After curing, the four-ply composite panel was cut into strips using a bandsaw, after which each strip was machined to ~60 mm by 12.5 mm by 1 mm in accordance with standards outlined in ASTM D3039.

### Recycling of 1/TREN matrix

A matrix composed of **1**/TREN (15.0 g) was heated in AcOH (100 ml) at 100°C for 24 hours. The digestion solution was concentrated via rotary evaporation to remove excess AcOH. Deionized H_2_O (500 ml) was added to the crude material and then transferred to a separatory funnel, and lactone **1** was extracted with 20:80 EtOAc:hexanes (3 × 250 ml). The organic layer was collected, dried with Na_2_SO_4_, and concentrated, affording **1** as a faint beige liquid residue (5.67 g, 62% yield). Over the course of three trials, the yields ranged between 45 and 62% recovery. The aqueous layer from the above solvent extraction was collected and concentrated by rotary evaporation. Excess KOH (11.4 g) was added to this brown residue and heated for 18 hours at 150°C. The crude residue was extracted with CHCl_3_, and the organic layer dried under vacuum to yield TREN as a yellow residue (1.82 g, 31% yield). Over the course of three trials, the yields ranged between 22 and 31% recovery. To a 20 ml scintillation vial, the recovered TREN (2.66 mmol, 1.82 g) was added followed by lactone **1** in a 25:1 *E*/*Z* isomeric ratio (18.6 mmol, 2.83 g). The resulting mixture was gently stirred with a wooden stick in a manner avoiding aerating the mixture and then transferred to a PTFE mold using a syringe. The yellow material was then heated in an oven at 80°C for 30 min followed by 150°C for an additional 3 hours.

## Supplementary Materials

### The PDF file includes:

Supplementary Materials Figs. S1 to S104 Tables S1 to S5 Legend for movie S1 References
