# Supplementary material for: Internally promoted reactivity of carbonized butadiene and amines enables a recyclable polymer composite
Source: Sci Adv. 2026 May 6;12(19):eaed1295. doi: 10.1126/sciadv.aed1295 (PMC13148295; doi:10.1126/sciadv.aed1295)
Supplement: Supplementary file 1 — Supplementary Materials Figs. S1 to S104 Tables S1 to S5 Legend for movie S1 References [file sciadv.aed1295_sm.pdf]

Science Advances
AAAS

Supplementary Materials for

Internally promoted reactivity of carbonized butadiene and amines enables a recyclable polymer composite

Keaton M. Turney *et al.*

Corresponding author: James M. Eagan, eagan@uakron.edu

Sci. Adv. **12**, eaed1295 (2026)
DOI: 10.1126/sciadv.aed1295

The PDF file includes:

Supplementary Materials
Figs. S1 to S104
Tables S1 to S5
Legend for movie S1
References

Other Supplementary Material for this manuscript includes the following:

Movie S1

Materials

Carbon dioxide (99.5%) and nitrogen (99.99%) were both purchased from Linde Gas & Equipment Company. Tris(dibenzylideneacetone)dipalladium(0) (97%) and tris(2-aminoethyl)amine (97%) were purchased from Thermo Fisher. A 25 lb tank of stabilized 1,3-butadiene (99%) was purchased from ChemSampCo. Tris(4-methoxyphenyl)phosphine (98%), bis(hexamethylene)triamine (>95%), diethylenetriamine (>98%), m-xylylenediamine (>99%), and p-xylylenediamine (>99%) were purchased from TCI. Hydroquinone (99%), acetonitrile (HPLC Grade, ≥99.9%), Celite S, hexanes (ACS Grade), diethyl ether (Et_2O, ACS Reagent Grade, ≥99.0%), N,N'-Bis(3-aminopropyl)ethylenediamine (98%), 3-aminomethyl-3,5,5-trimethylcyclohexylamine (>99%), Dichloromethane (ACS Reagent Grade, ≥99.5%), N, N-Dimethylformamide (HPLC grade, ≥99.9%), magnesium chloride (anhydrous) (98%), tin(II)ethylhexanoate (95%), 1,8-diazabicyclo[5.4.0]undec-7-ene (98%), 1,5,7-triazabicyclo[4.4.0]dec-5-ene (98%), hexamethyldisiloxane (NMR grade, ≥99.5%), 1,2-ethylenediamine (≥99%), diethylamine, triethylamine, 1,4 Bis(trimethylsilyl)benzene (96%) and bisphenol A diglycidyl ether were purchased from Sigma-Aldrich. Ethyl acetate (Certified ACS Grade ≥99.5%), acetone (ACS Grade, ≥99.5%), methanol (Certified ACS Grade, ≥99.8%), toluene (Certified ACS Grade) (≥99.7%), and hydrochloric acid (Certified ACS Plus Grade) were purchased from Fisher Chemical. Trans-1,4-diaminocyclohexane (≥,98%) 1,6-hexanediamine (≥99.5%), tert-butylamine (99%), and deuterium oxide (*d*, 99.8%) were purchased from Acros. Chloroform-*d* (*d,* 99.8%) and Dimethyl sulfoxide-d_6 (*d*, 99.9%) were purchased from Cambridge Isotope Laboratories, Inc. Diisopropylamine (99%) was purchased from Beantown Chemical. Jeffamine D230 was purchased from Huntsman. Zinc chloride (≥98%) was purchased from Fluka Chemical. Acetic acid (Glacial) (≥99.7%) was purchased from VWR. Silica gel was purchased from Silicycle. FibreGlast 696-A Unfinished Edge Carbon Fiber Tape (6" x 10 yd) was purchased from FibreGlast. Multipurpose Flame-Retardant Garolite G-10/FR4 sheet (1/16" thick x 12" wide x 12" long) was purchased from McMaster-Carr. Loctite E-120HP adhesive, Loctite Multiple Ratio Two-Part Applicator, and Loctite Mixing Nozzle: A Style, for 1:2 mix ratios were purchased from Grainger.

^{1}H NMR of Small Molecules

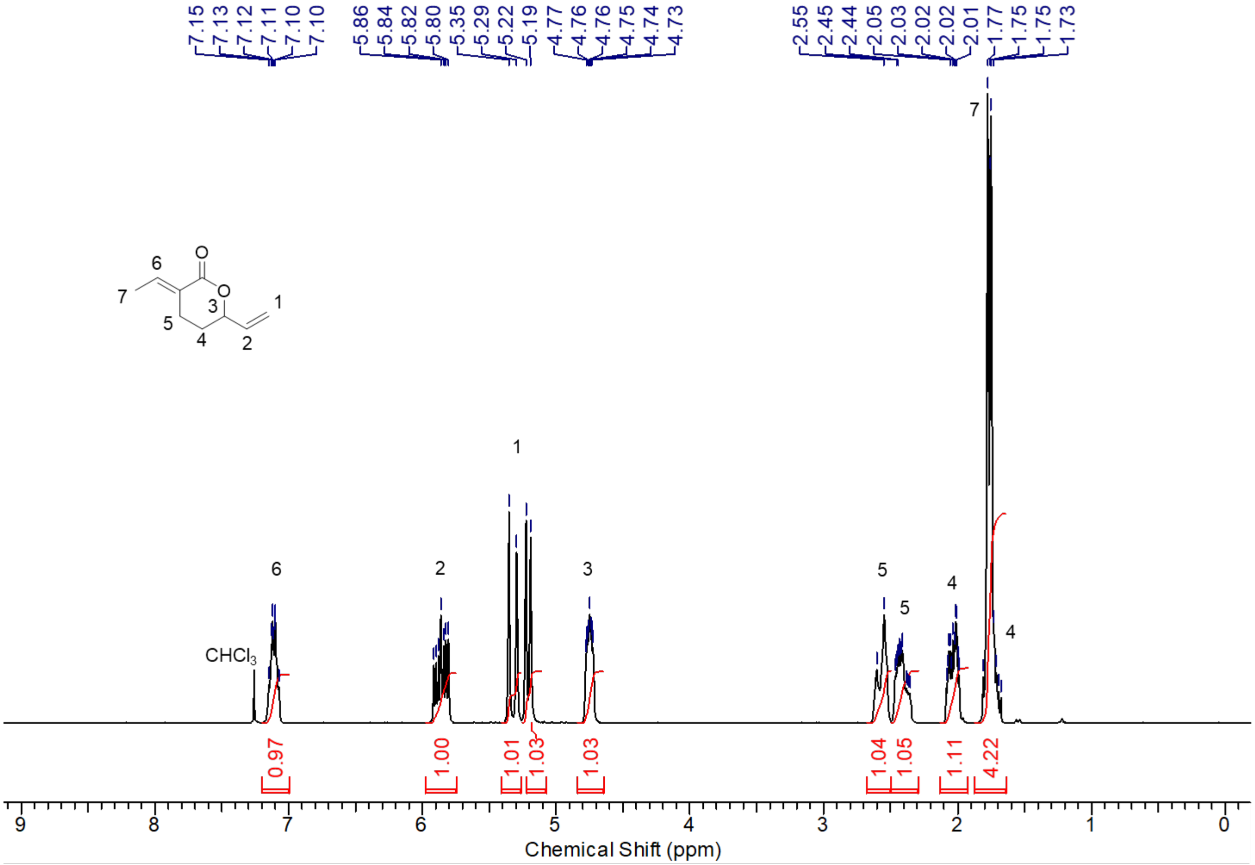

Fig. S1. ^{1}H NMR (500 MHz, $CDCl_3$) of lactone **1**.

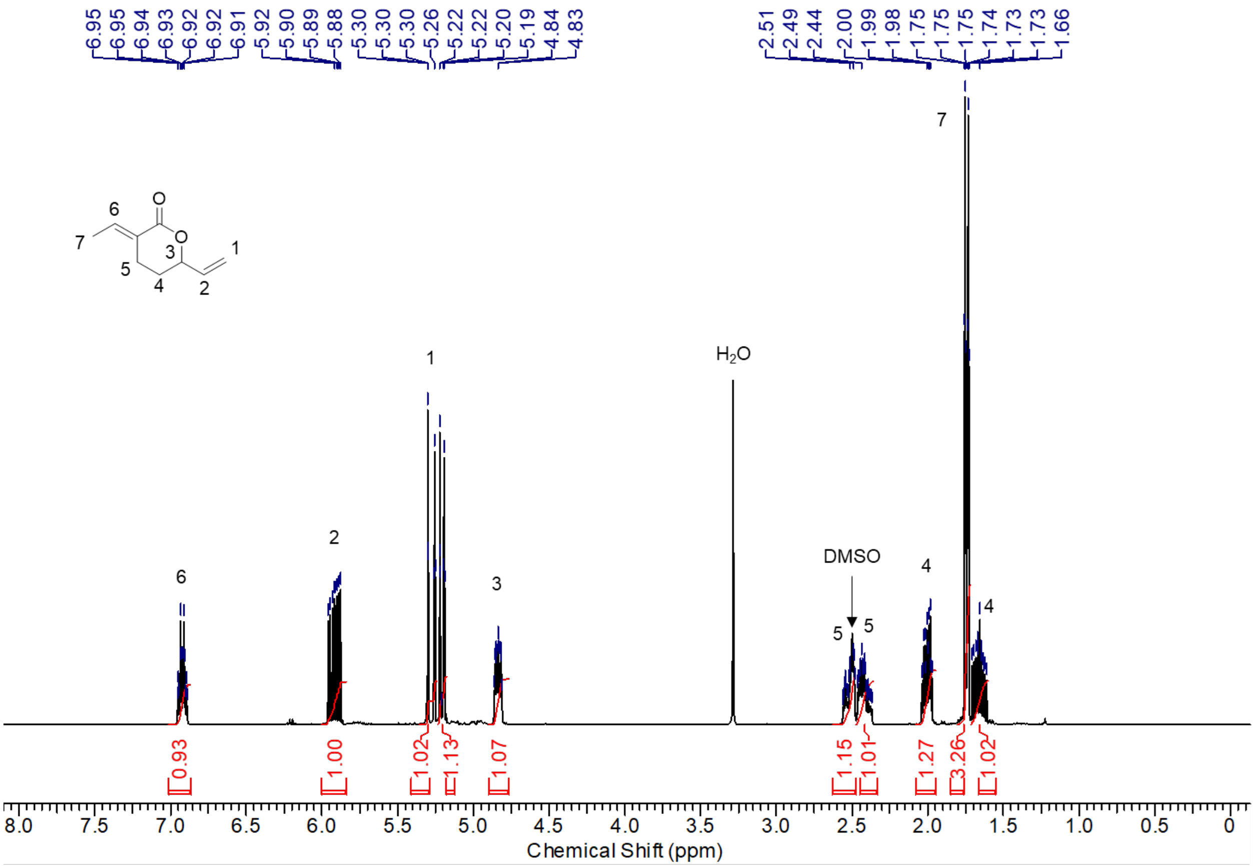

Fig. S2. ^{1}H NMR (500 MHz, DMSO-d_6) of lactone **1**.

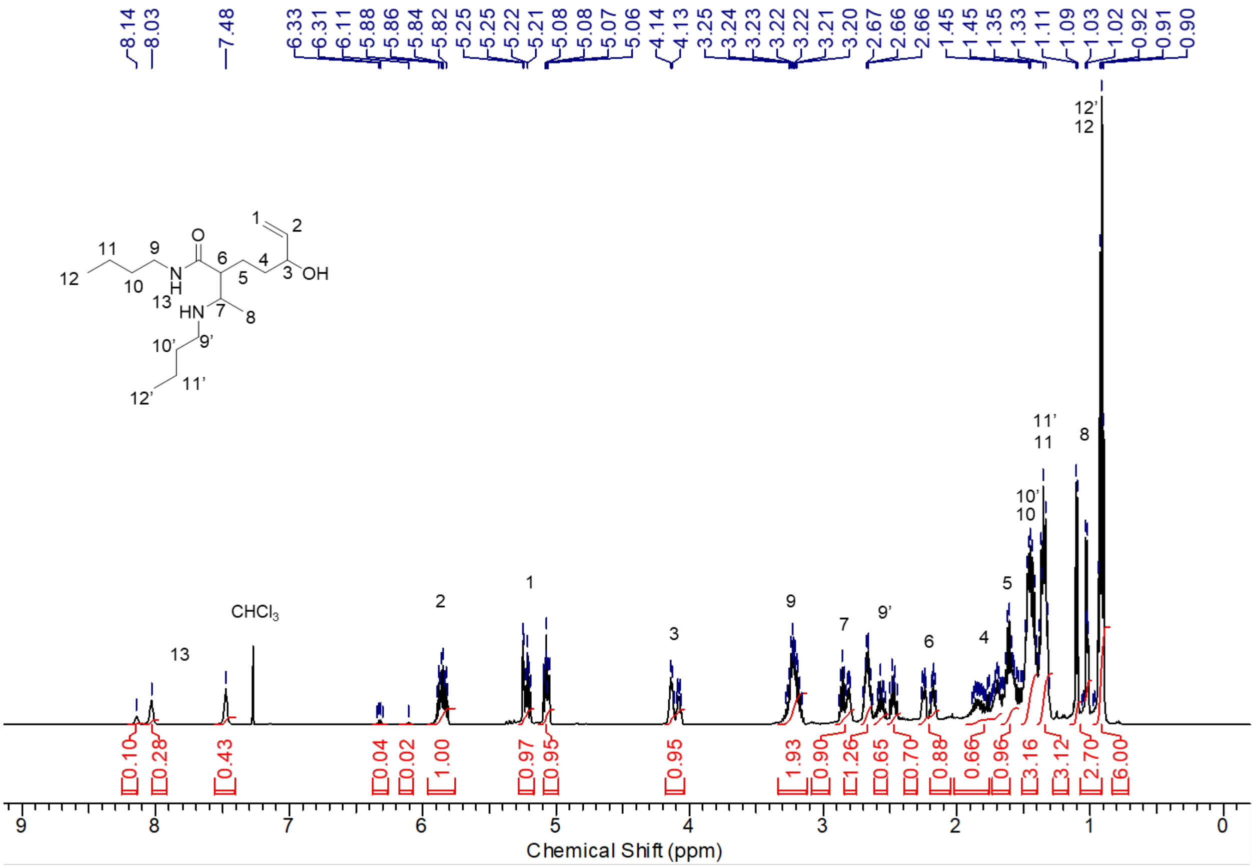

Fig. S3. ^{1}H NMR (500 MHz, $CDCl_3$) of amidoamine **2**.

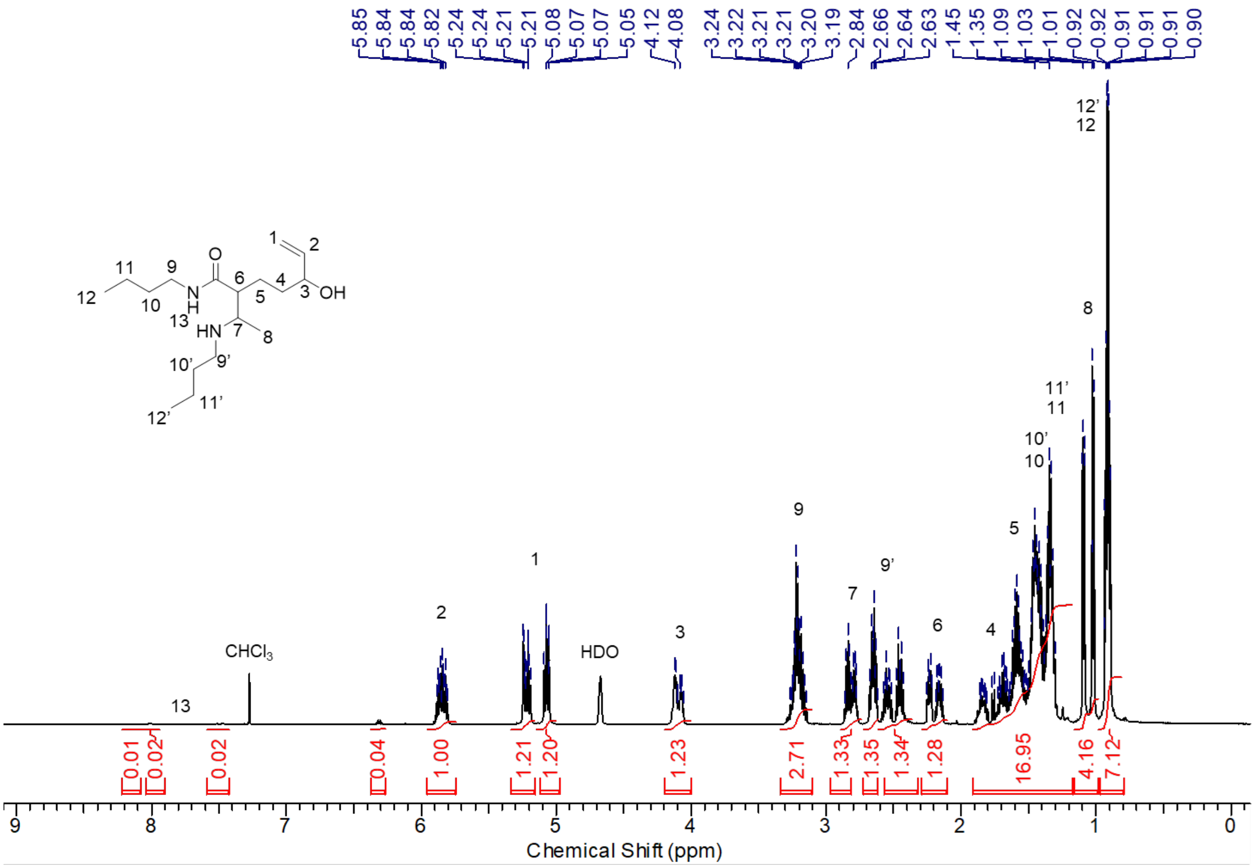

Fig. S4. ^{1}H NMR (500 MHz, $CDCl_3$, doped with D_2O) of amidoamine **2**.

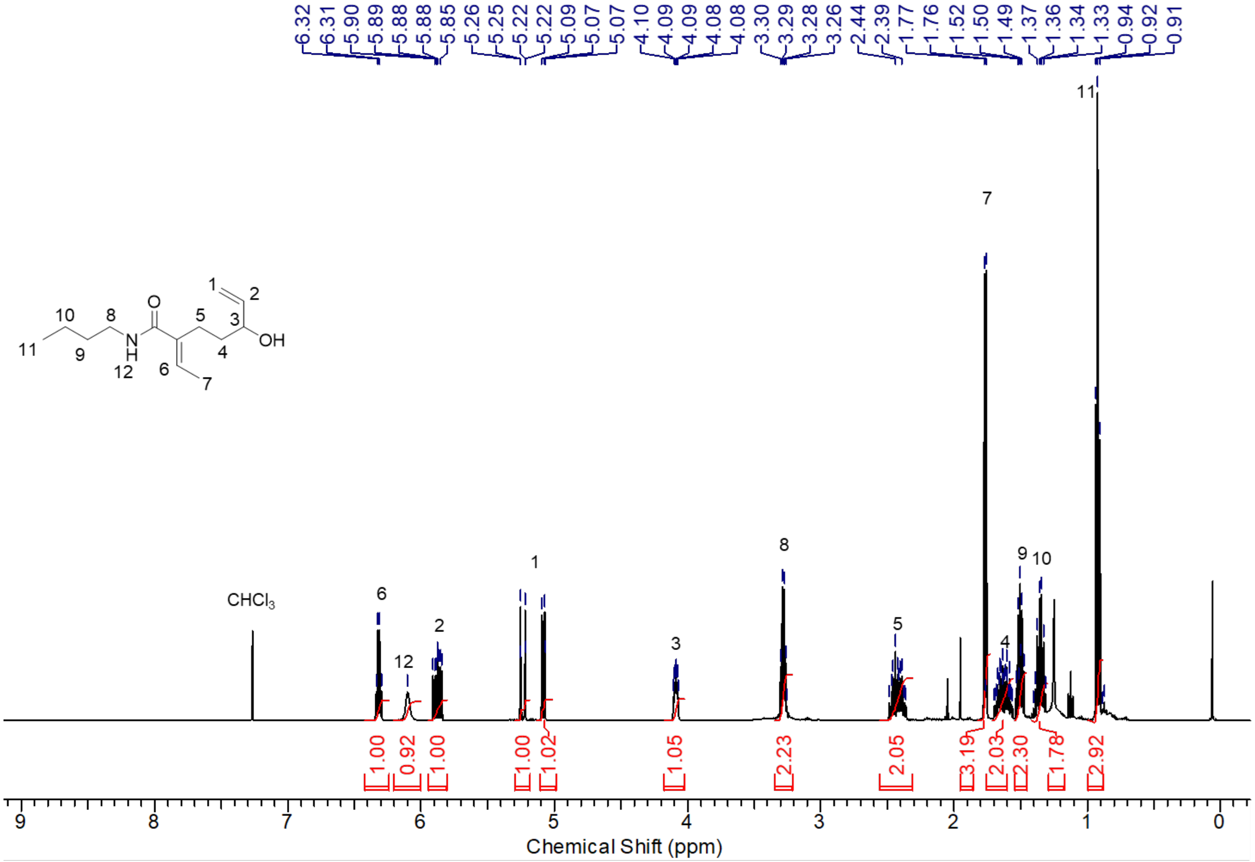

Fig. S5. ^{1}H NMR (500 MHz, $CDCl_3$) of tiglamide **3**.

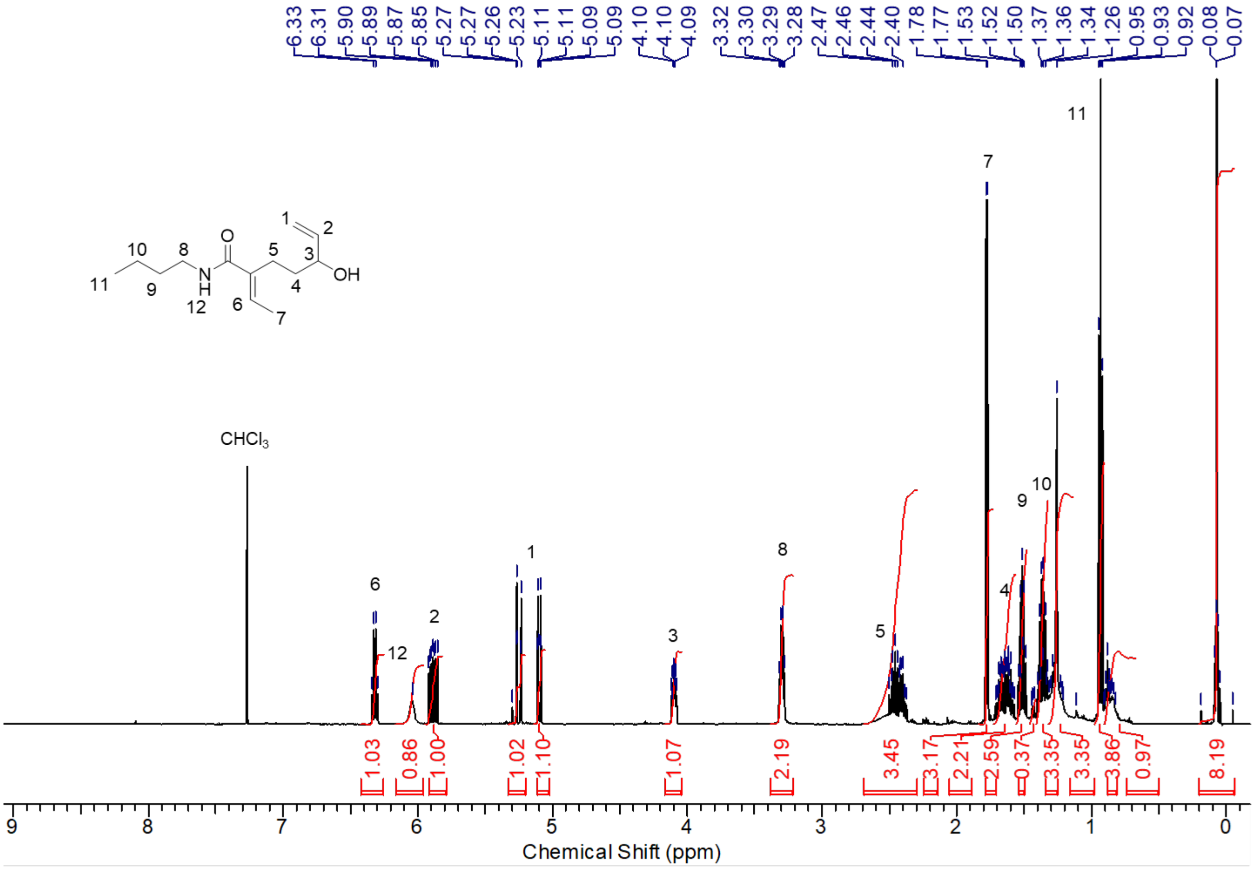

Fig. S6. ^{1}H NMR (500 MHz, CDCl$_3$) of tiglamide **3** following a sodium bicarbonate wash, an investigation which demonstrates that the minor product is not an acid species.

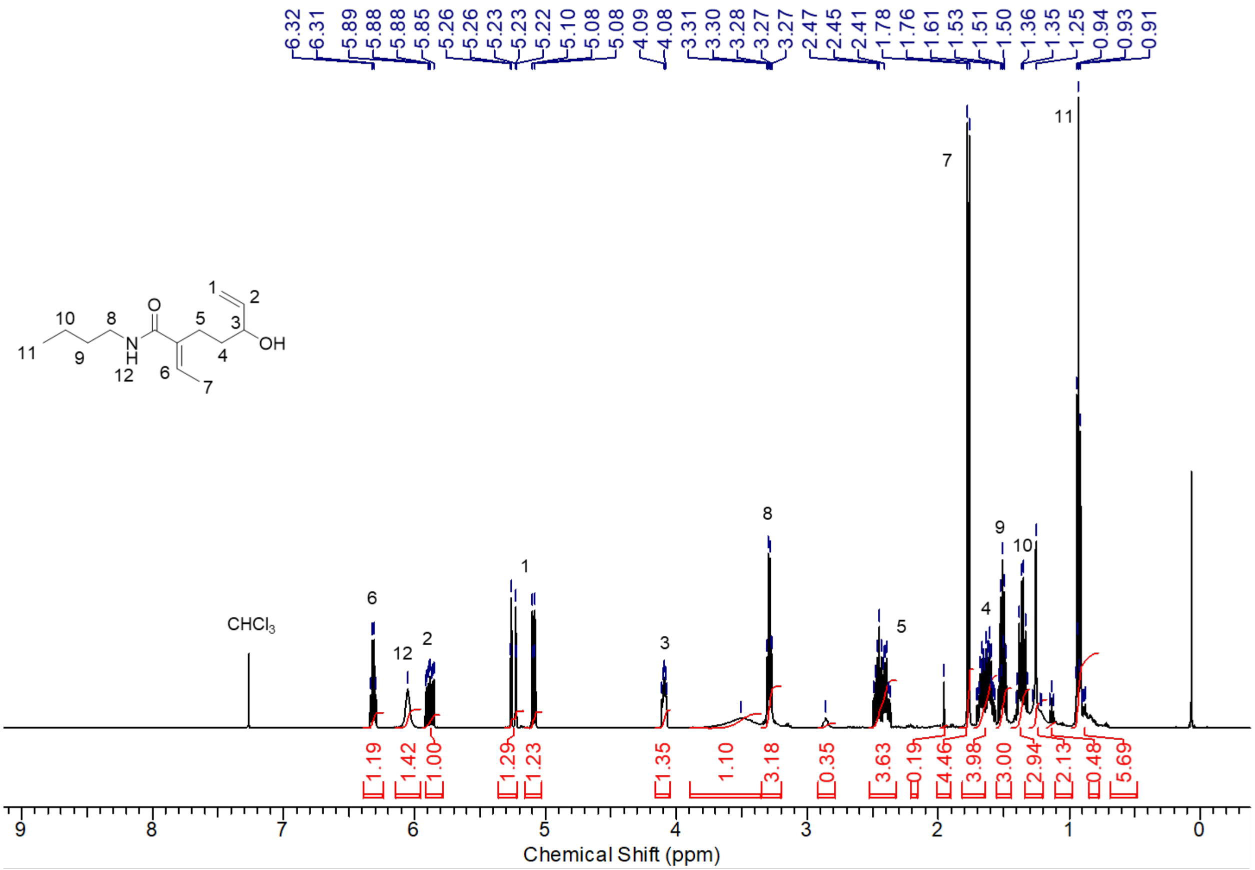

Fig. S7. ^{1}H NMR (500 MHz, $CDCl_3$) of tiglamide **3** following resubjection to *n*-butylamine (10 equivalents) at 25°C for 24 hours.

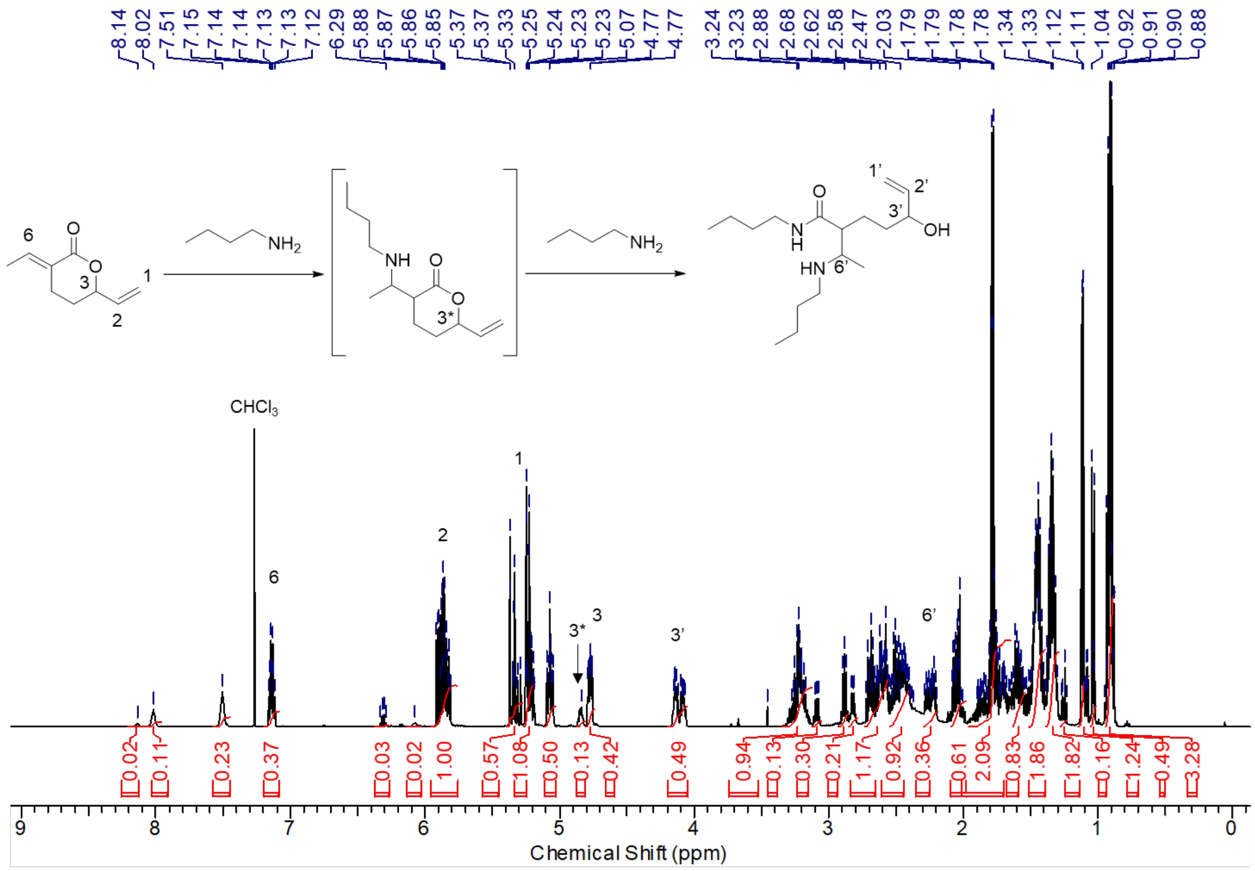

Fig. S8. ^{1}H NMR (500 MHz, $CDCl_3$) of 1:1 molar ratio **1**:*n*-butylamine after 24 hours.

^{13}C NMR of Small Molecules

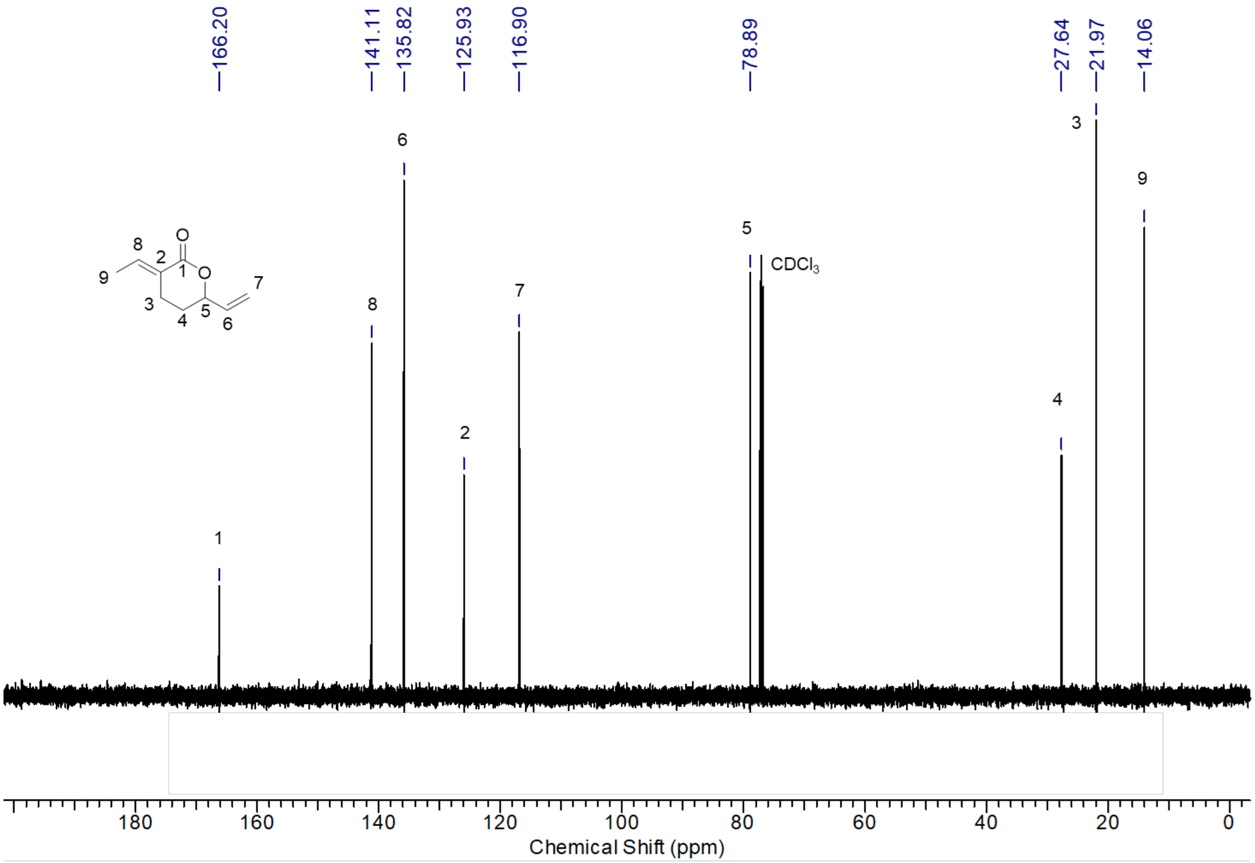

Fig. S9. ^{13}C NMR (125 MHz, $CDCl_3$) of lactone **1**.

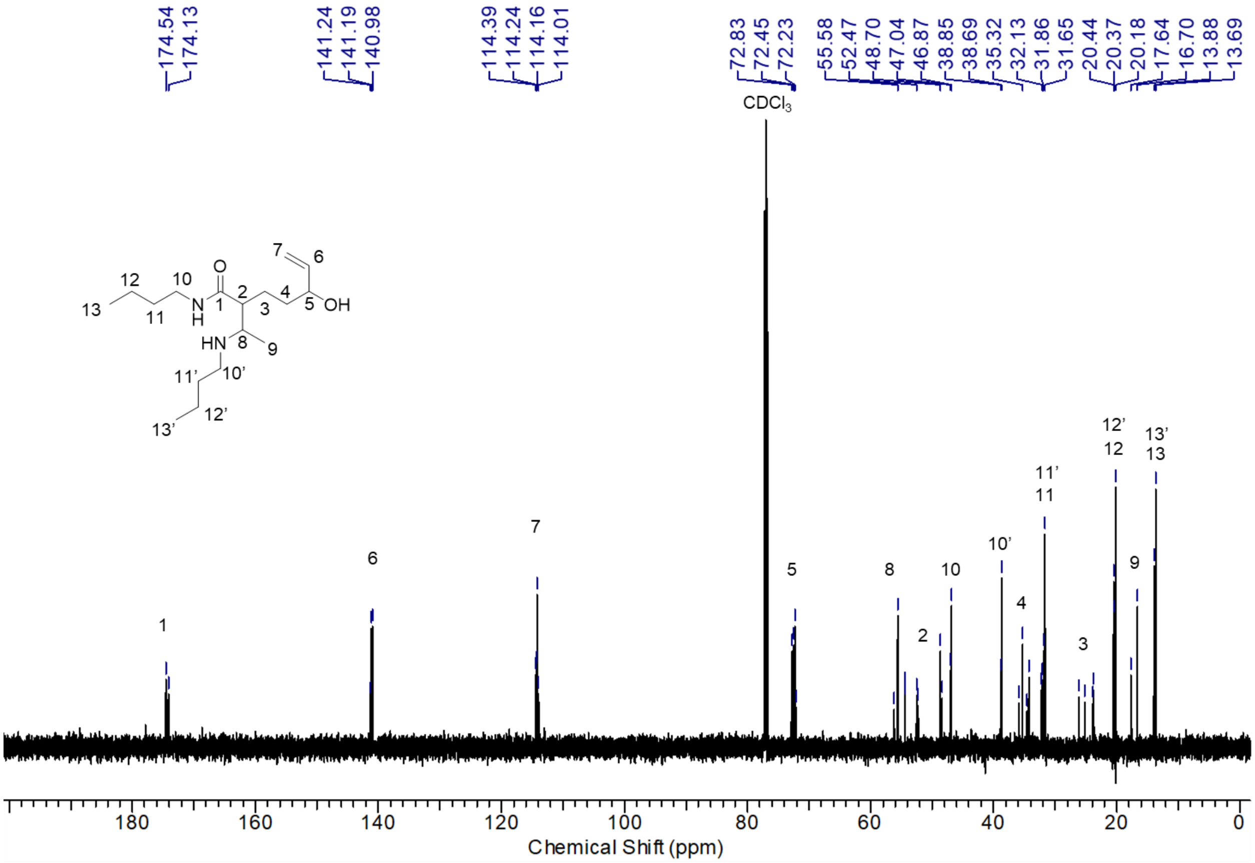

Fig. S10. ^{13}C NMR (125 MHz, $CDCl_3$) of amidoamine **2**.

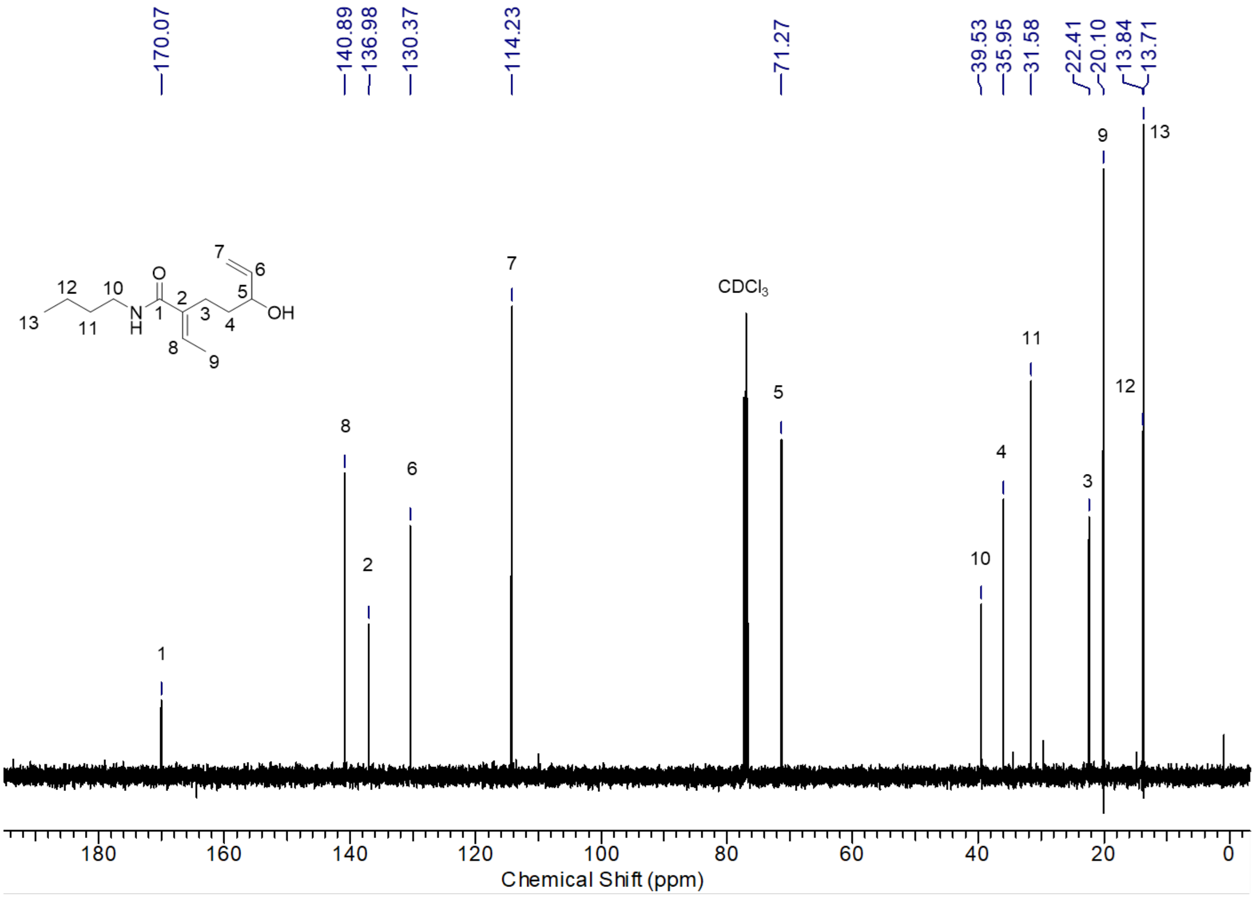

Fig. S11. ^{13}C NMR (125 MHz, $CDCl_3$) of tiglamide **3**.

2D NMR of Small Molecules

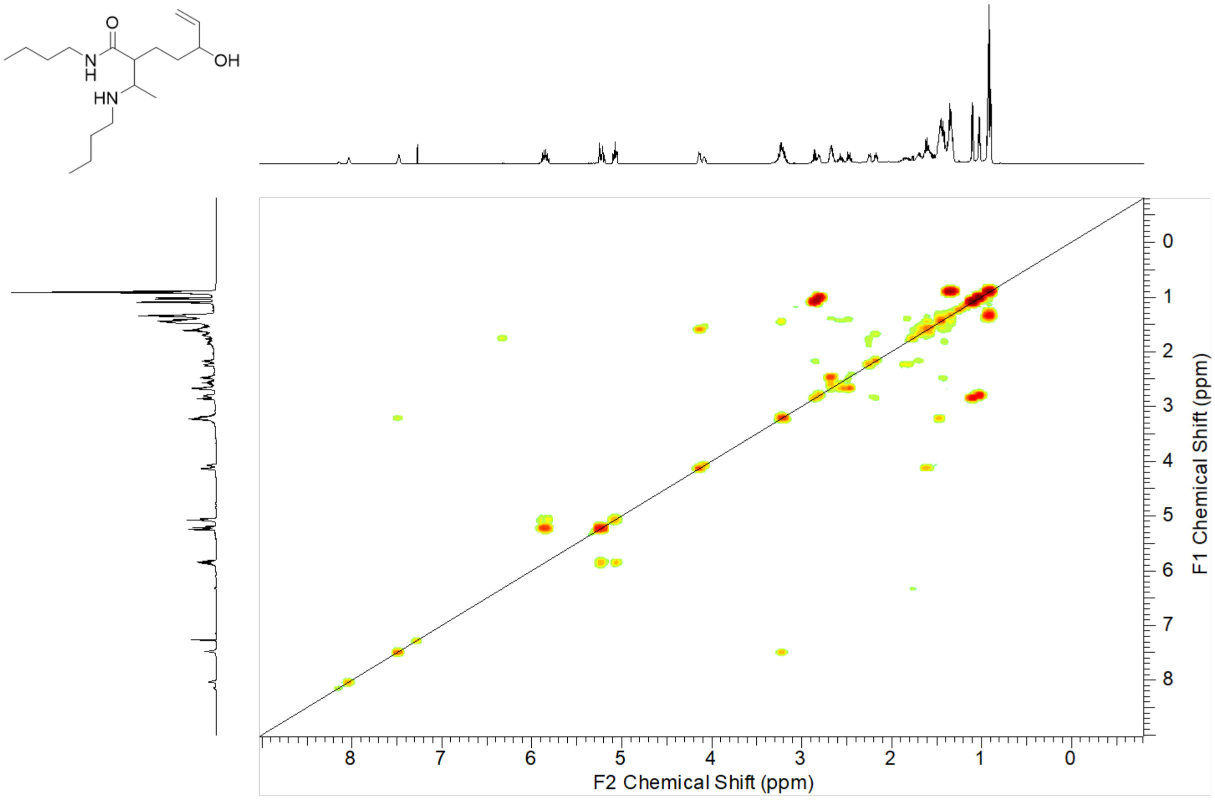

Fig. S12. Correlation Spectroscopy (COSY) NMR (500 MHz, $CDCl_3$) of amidoamine **2**.

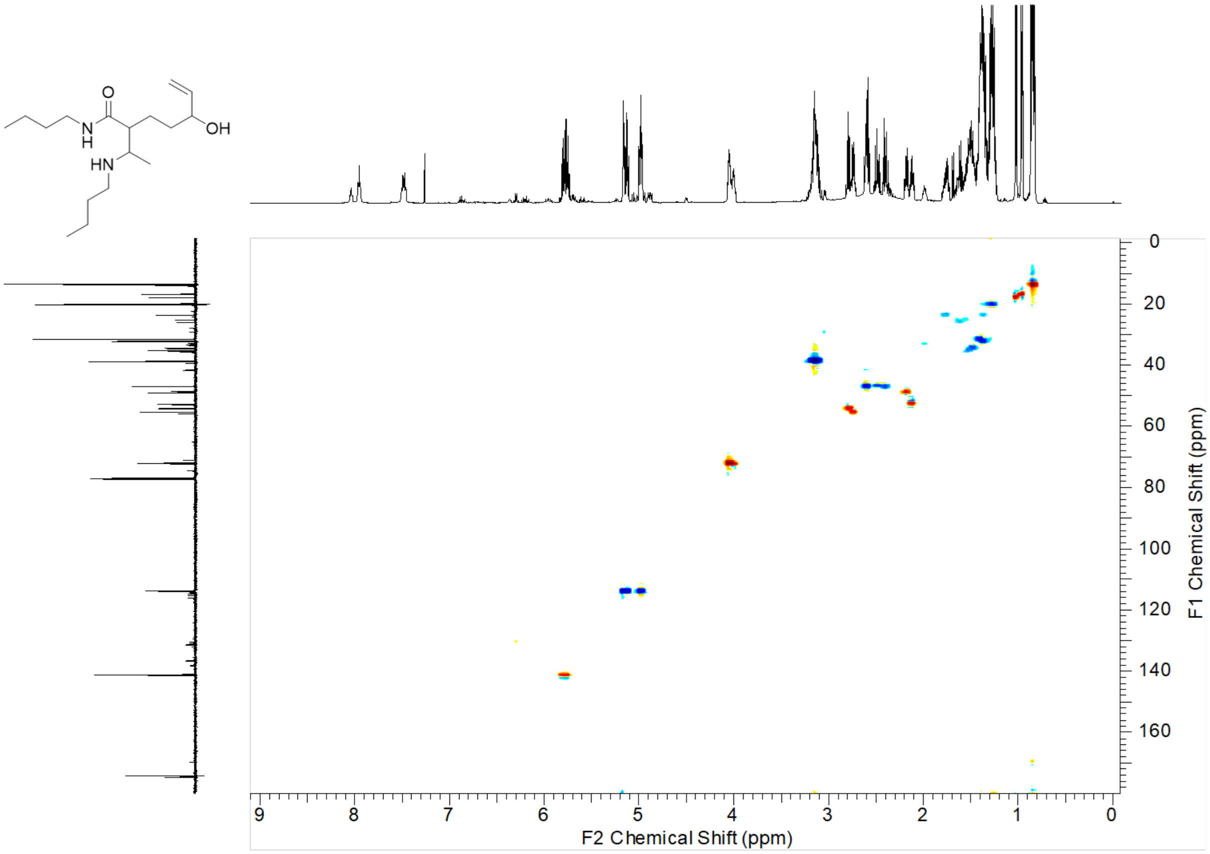

Fig. S13. Heteronuclear Single Quantum Coherence (HSQC) NMR (500 MHz, 125 MHz, $CDCl_3$) of amidoamine **2**.

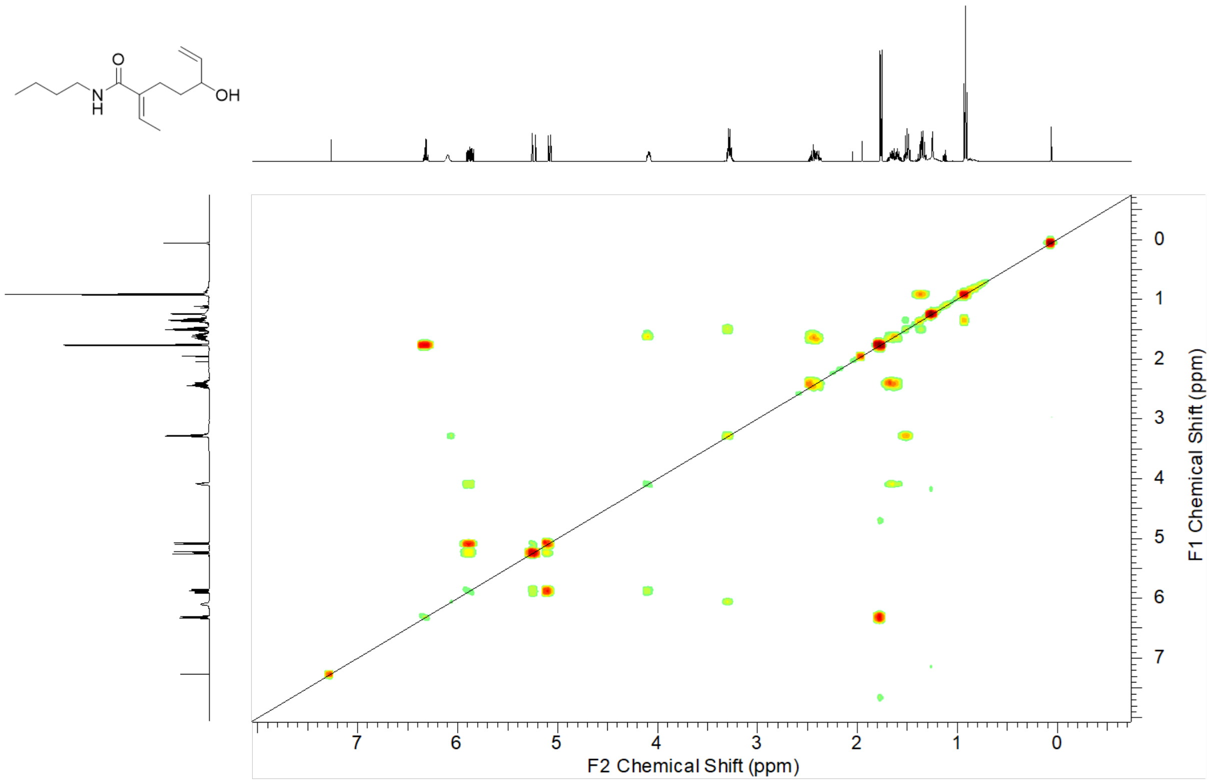

Fig. S14. COSY NMR (500 MHz, $CDCl_3$) of tiglamide **3**.

DFT Calculations

Density functional theory (DFT) was used to explore the potential energy surface of the reaction pathway. All calculations were performed using Gaussian16 software, with the B3LYP functional and 6-311++G(d,p) basis set selected for optimizations and harmonic frequency analyses in gas phase. Transition structures were identified by a single negative eigenvalue in the Hessian matrix, and the Intrinsic Reaction Coordinate (IRC) method was applied to confirm the transition states. For non-transition structures, frequency calculations ensured that all eigenvalues of the Hessian matrix were positive. Computed free energy values correspond to a temperature of 298 K and a pressure of 1 atm.

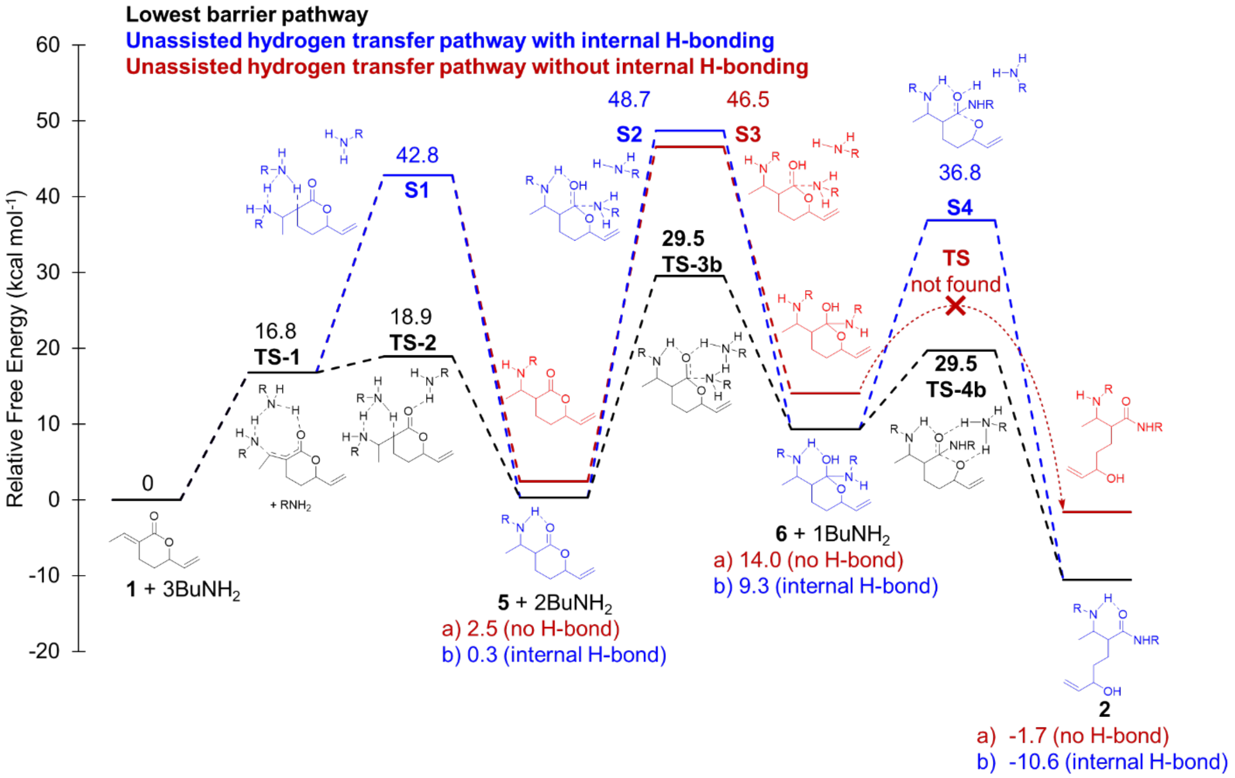

Fig. S15. Free energy diagram of amidoamination comparing the assisted hydrogen transfer pathway (black) to the unassisted hydrogen transfer pathways (blue, red). The transition state for unassisted ring opening without H-bonding (**6a**→**2**) was not found after repeated optimizations, suggesting an excessive energy barrier.

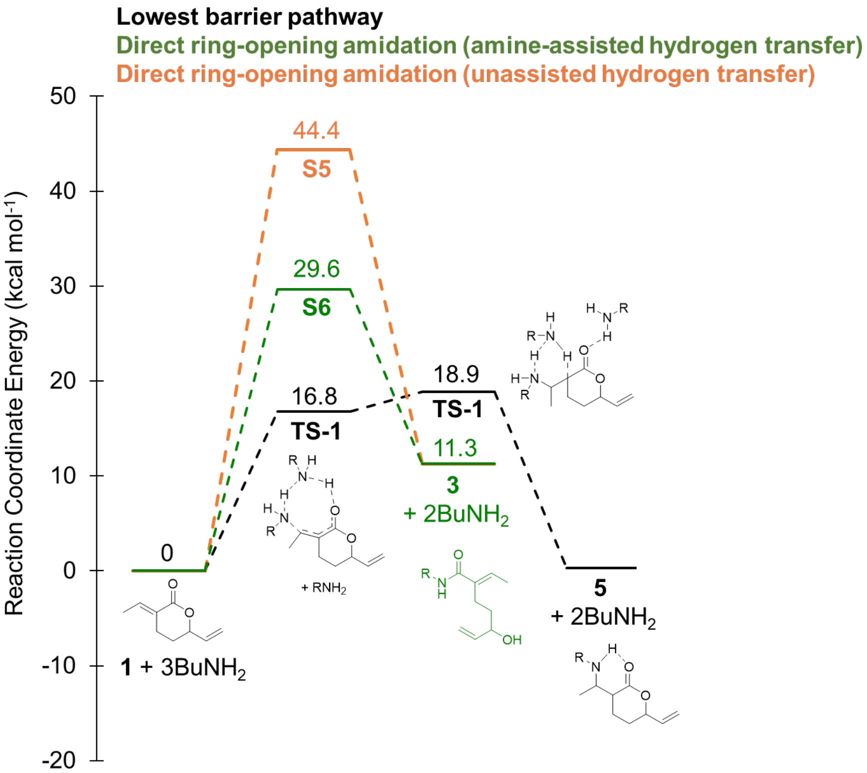

Fig. S16. Free energy diagram of amidoamination comparing the preferred assisted hydrogen transfer pathway from Fig. 4 of main text (black) to the direct ring-opening amidation pathways (green, orange).

NMR Comparisons with Literature

Table S1. Comparison of ^{1}H and ^{13}C NMR chemical shifts (500 MHz, 125 MHz, $CDCl_3$) of observed materials versus products previously reported in literature.(*43–45*)

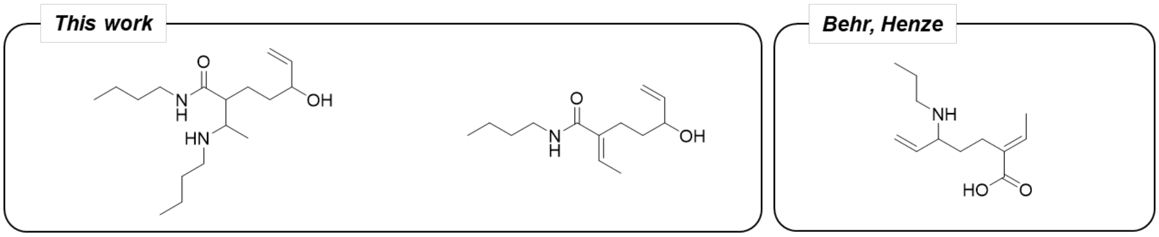

Amidoamine		Tiglamide		Amino acid (Behr)	
^{1}H δ (ppm), m, #H	^{13}C δ (ppm)	^{1}H δ (ppm), m, #H	^{13}C δ (ppm)	^{1}H δ (ppm), m, #H	^{13}C δ (ppm)
8.12, 8.02, 7.47, br s, 1H	174.5	6.32, q, 1H	170.1	6.27	171.5
5.85, m, 1H	141.2	6.10, s, 1H	140.9	5.83	142.4
5.22, m, 1H	114.4	5.88, ddd, 1H	137.0	5.18	138.2
5.07, m, 1H	72.8	5.24, dt, 1H	130.4	5.03	132.1
4.09, m, 1H	55.6	5.08, dt, 1H	114.2	3.90	115.6
3.20, m, 2H	46.9	4.09, ddd, 1H	71.3	3.20	72.7
2.82, m, 1H	38.7	3.29, m, 2H	39.5	2.38	43.0
2.58, m, 2H	35.3	2.42, m, 2H	36.0	1.72	37.4
2.21, m, 1H	31.7	1.76, d, 3H	31.6	1.65 – 1.47	24.2
1.84, m, 1H	25.2	1.62, m, 2H	22.4	0.88	23.8
1.70, m, 1H	20.2	1.50, quin, 2H	20.1		15.3
1.59, m, 2H	16.7	1.35, dq, 2H	13.8		12.8
1.44, m, 4H	13.7	1.25, s, 1H	13.7		
1.34, dt, 4H		0.92, t, 3H			
1.06, m, 3H					
0.92, m, 6H					

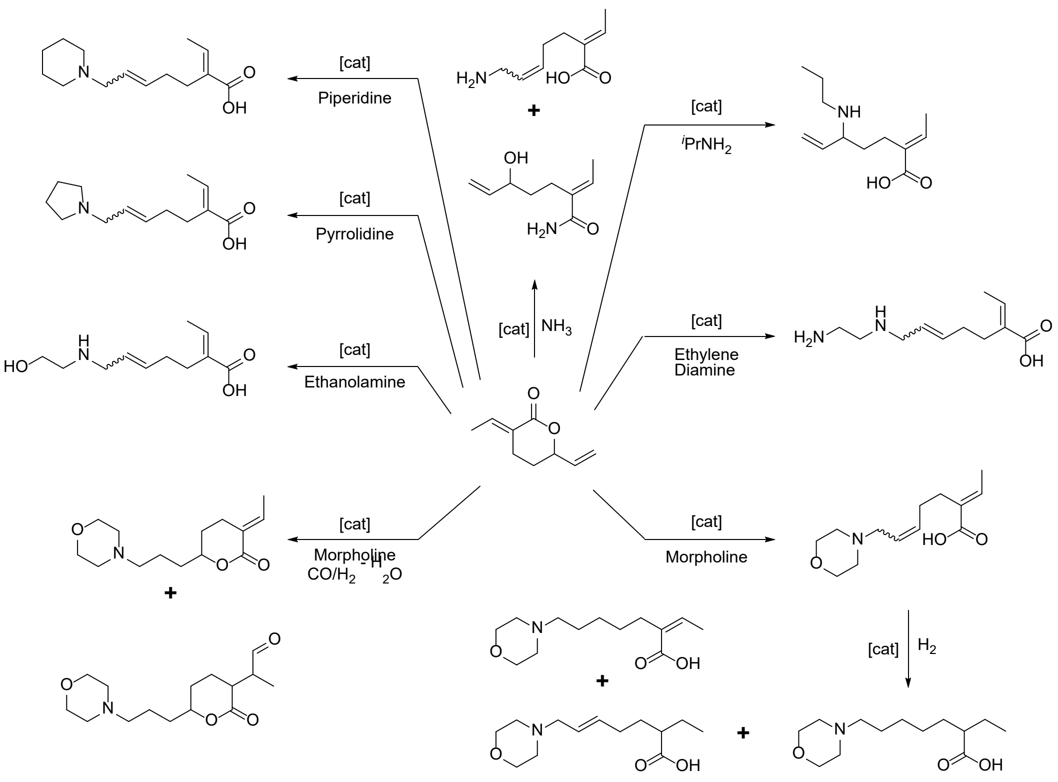

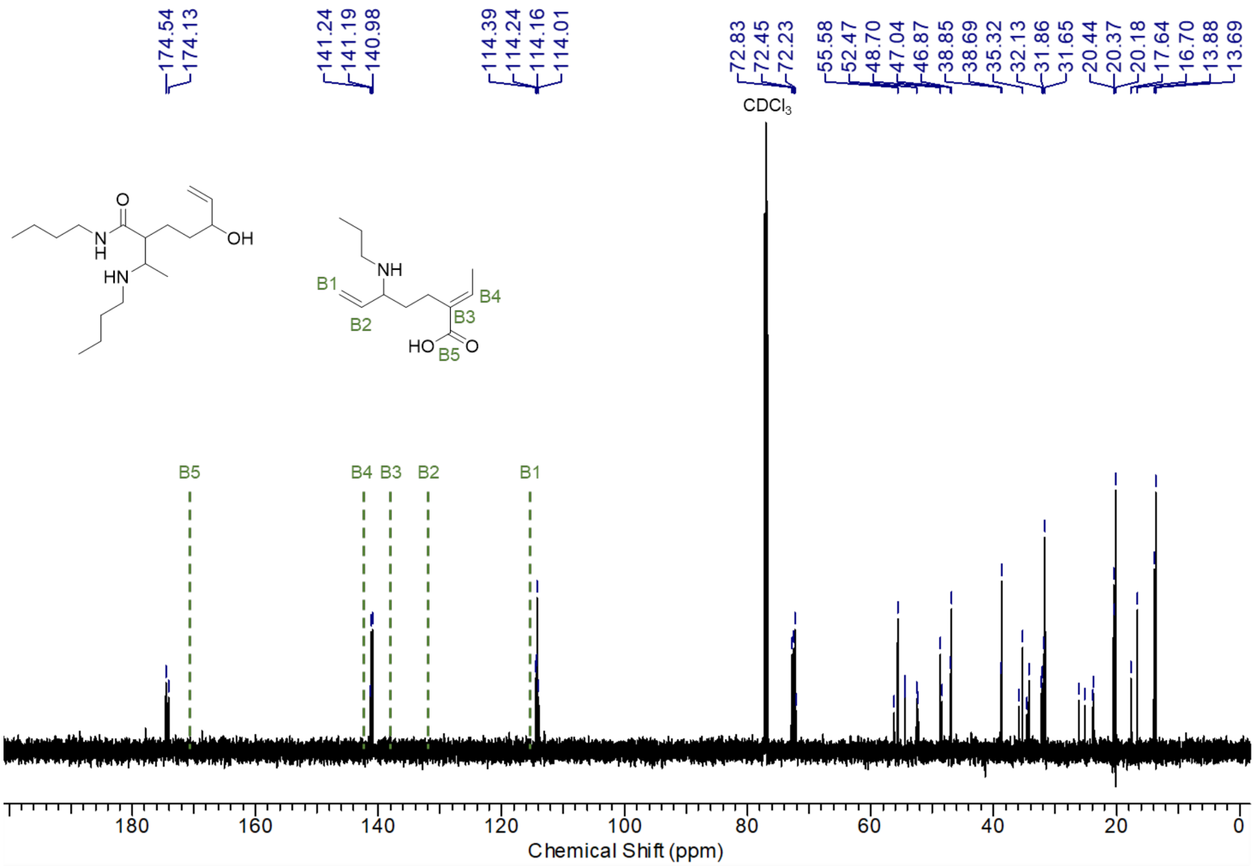

Fig. S17. ^{13}C NMR (125 MHz, $CDCl_3$) of amidoamine **2**. Signals of interest from the *n*-propylamine amino acid adduct are annotated (green) as reported by Henze and Behr and shown to be absent in experimental spectra of amidoamine **2**.(*45*)

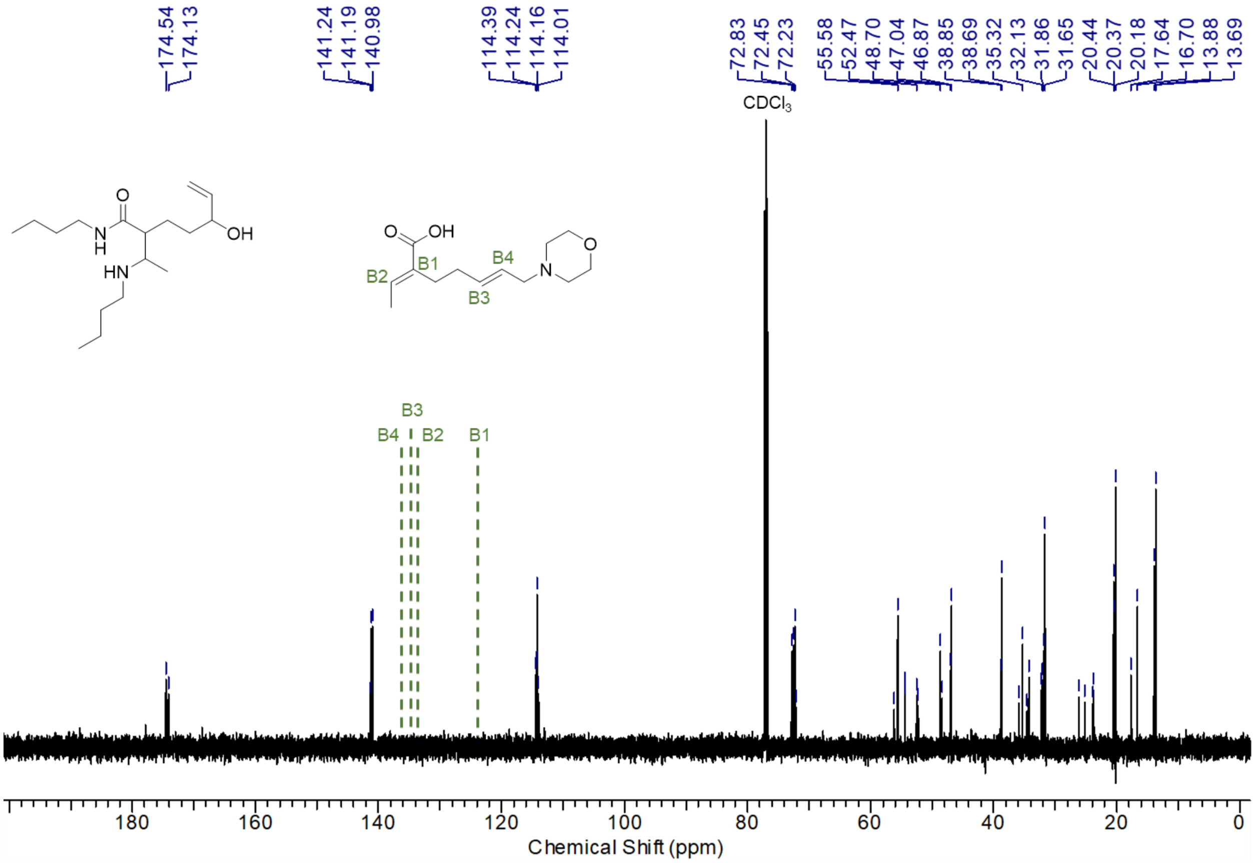

Fig. S18. ^{13}C NMR (125 MHz, $CDCl_3$) of amidoamine **2**. Signals of interest from the morpholine amino acid adduct are annotated (green) as reported by Henze and Behr in literature and shown to be absent in experimental spectra of amidoamine **2**.(*43*)

Mass Spectrometry of Small Molecules

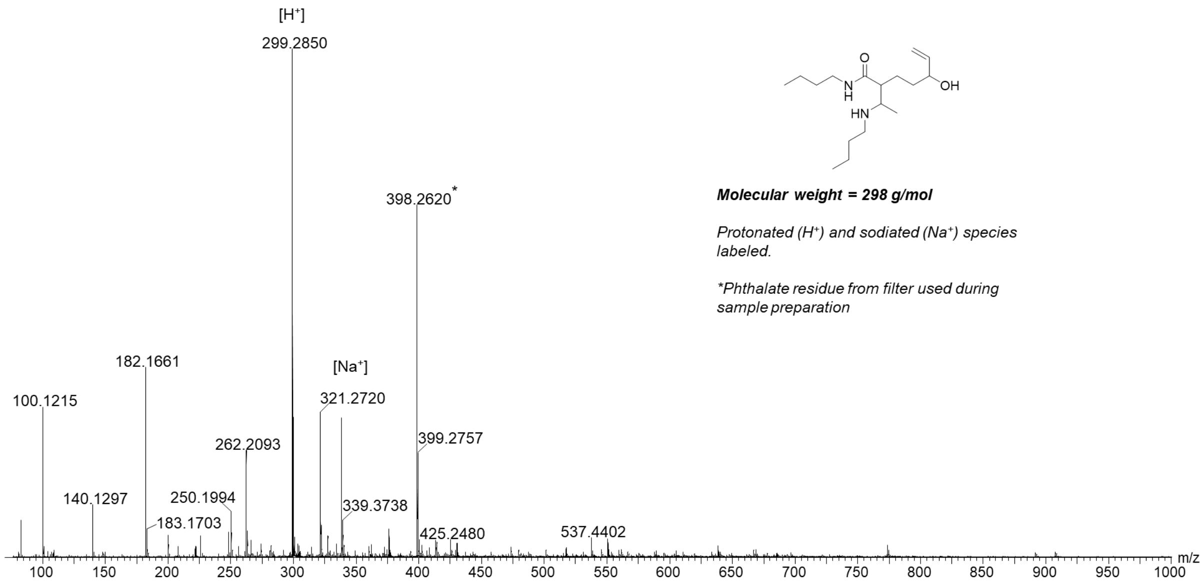

Fig. S19. ESI-MS of amidoamine **2**. 182 m/z is attributed to the methanolysis of a Lactone **1** (ESI-MS sample was prepared in MeOH). 262 m/z is attributed to the potassiated species of an oxidized dibutylamidoamine fragmenting a butylamine unit. 299 m/z is attributed to the protonated main bisbutylamidoamine product. 321 m/z is attributed to the sodiated bisbutylamidoamine product.

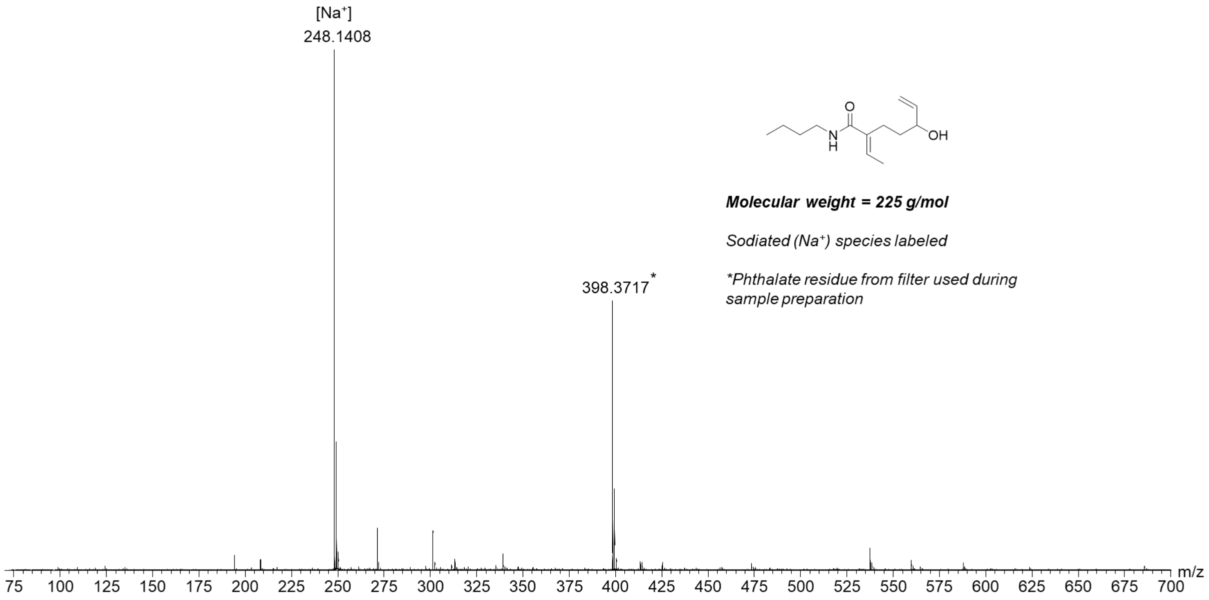

Fig. S20. ESI-MS of tiglamide **3** + Na^{+}.

ATR-FTIR of Small Molecules

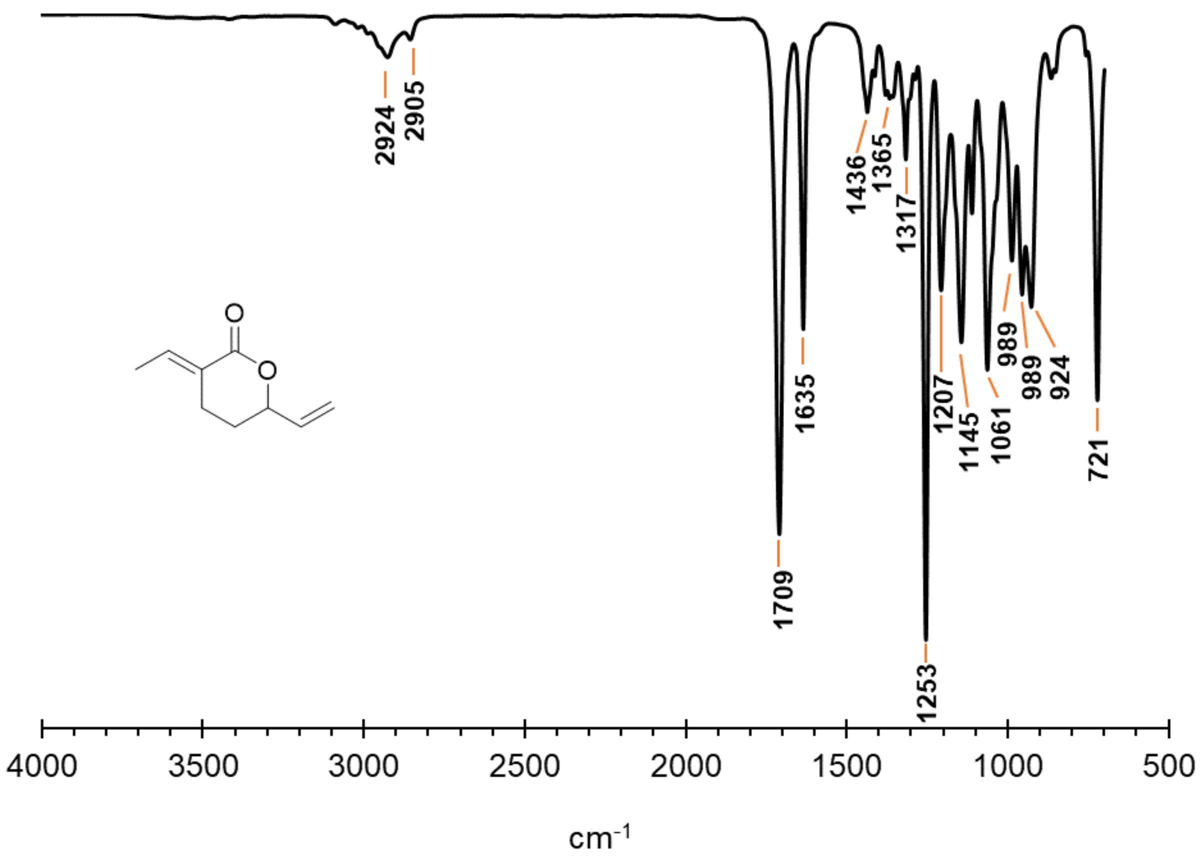

Fig. S21. ATR-FTIR of lactone **1.**

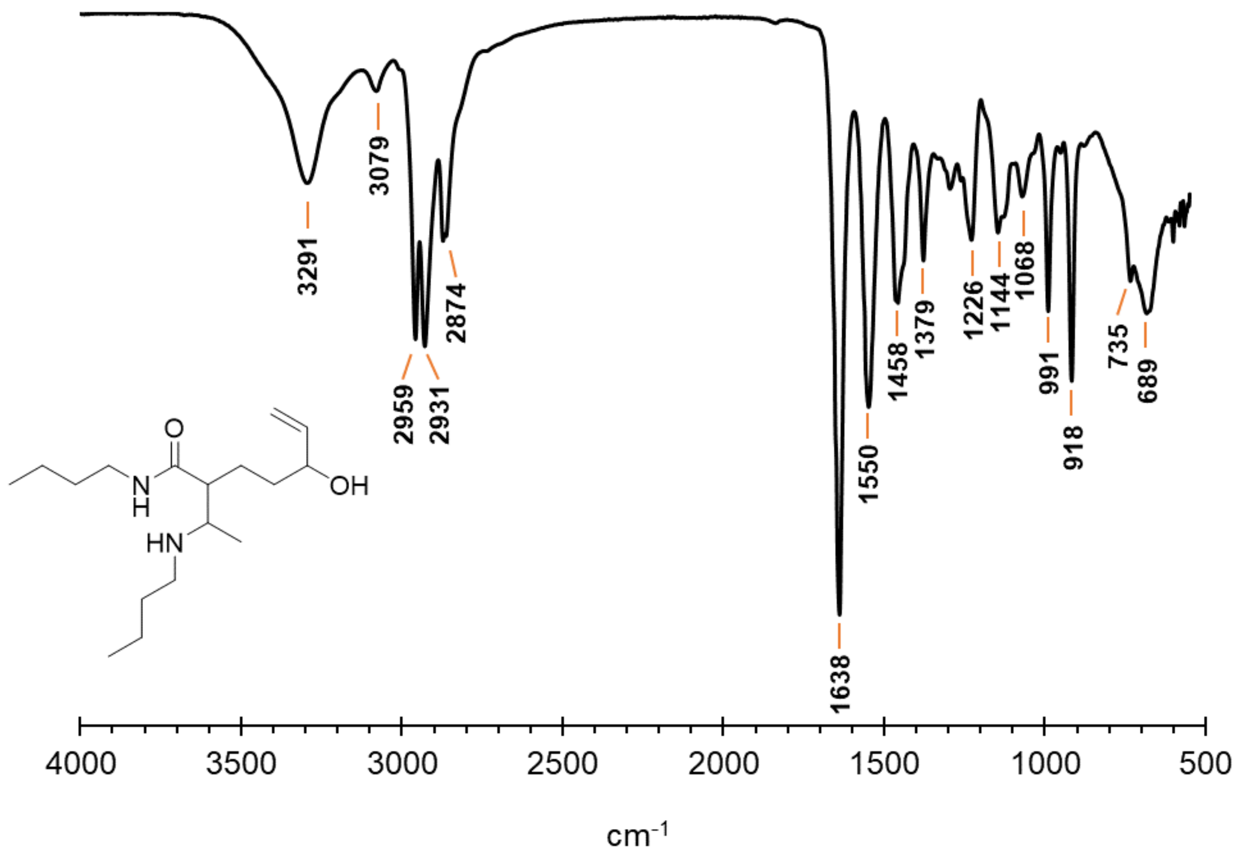

Fig. S22. ATR-FTIR of amidoamine **2**.

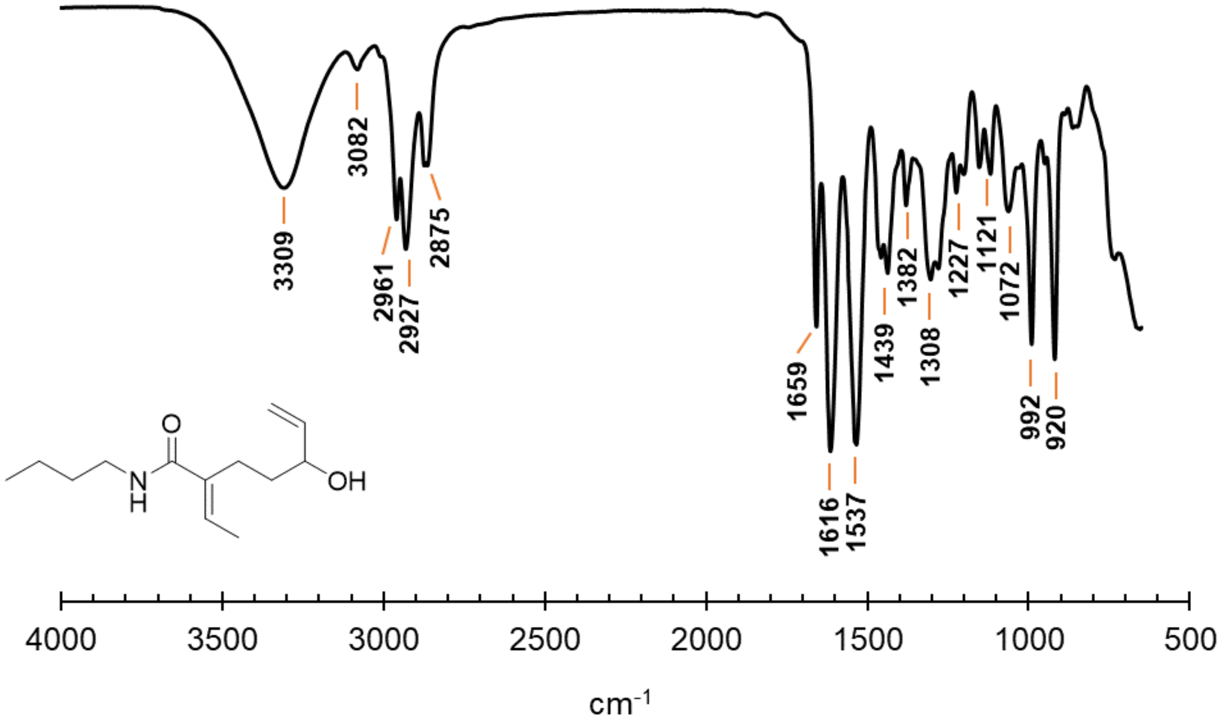

Fig. S23. ATR-FTIR of tiglamide **3**.

^{1}H NMR of Poly(amidoamine)

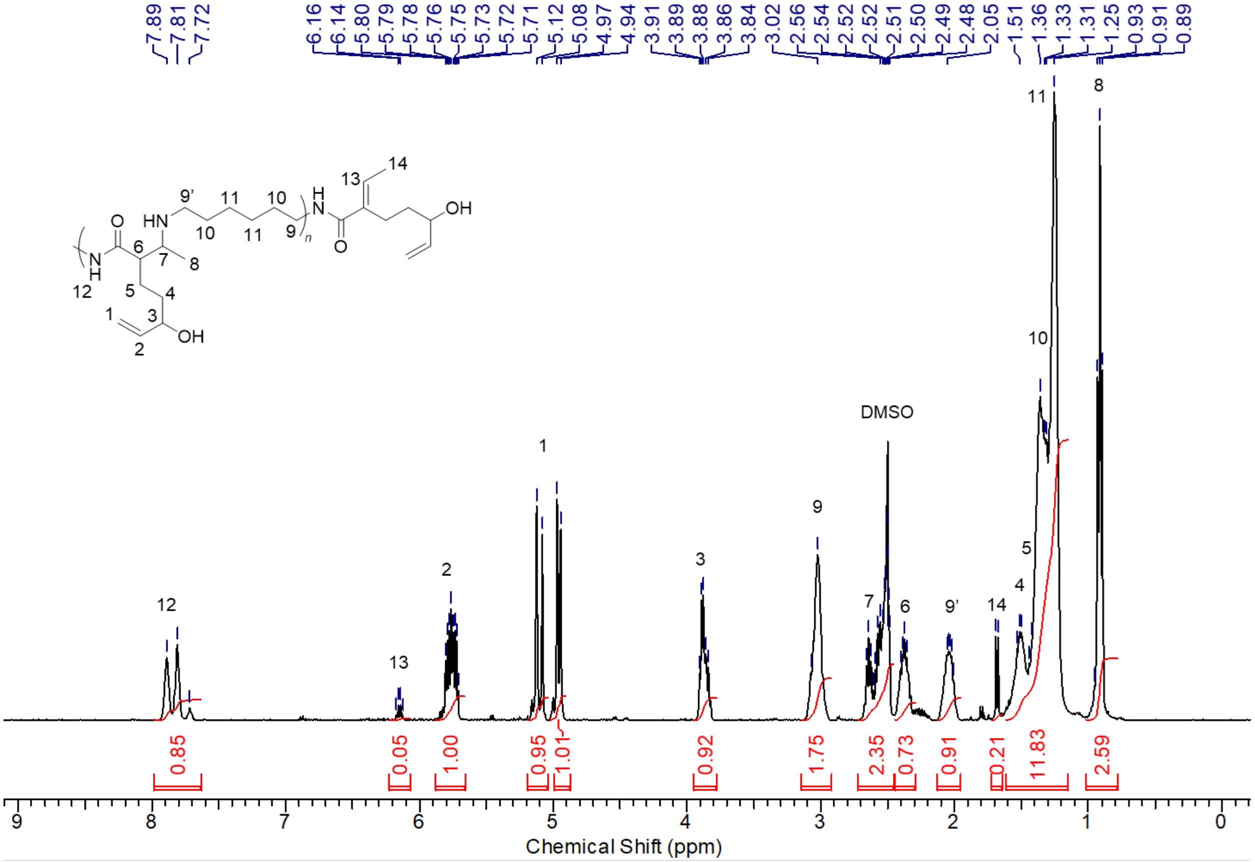

Fig. S24. ^{1}H NMR (500 MHz, DMSO-d_6) of poly(**1**/1,6-hexanediamine). Note that the solubility of the polymer is poor in chloroform, requiring the use of DMSO as a solvent for the following polymeric species.

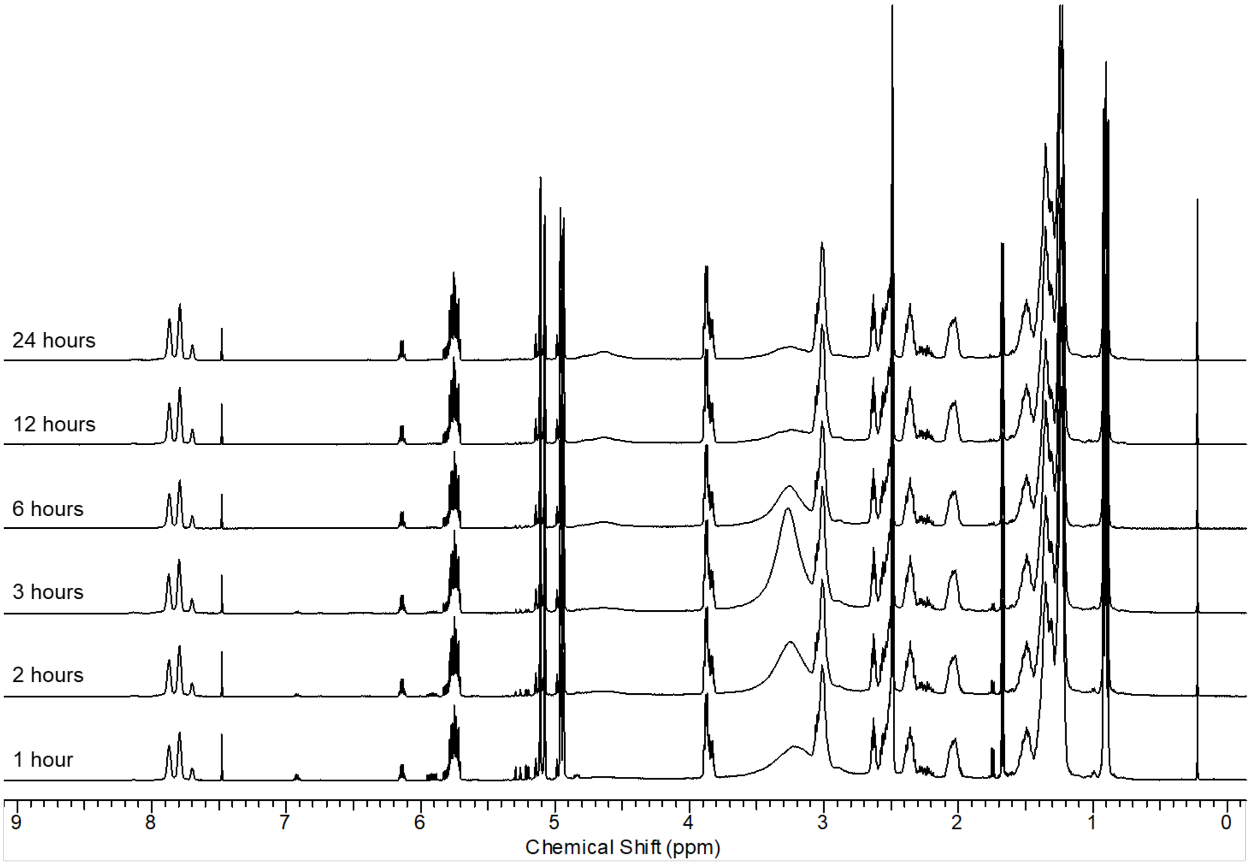

Fig. S25. Elapsed ^{1}H NMR (500 MHz, DMSO-d_6) overlay of poly(**1**/1,6-hexanediamine) synthesis.

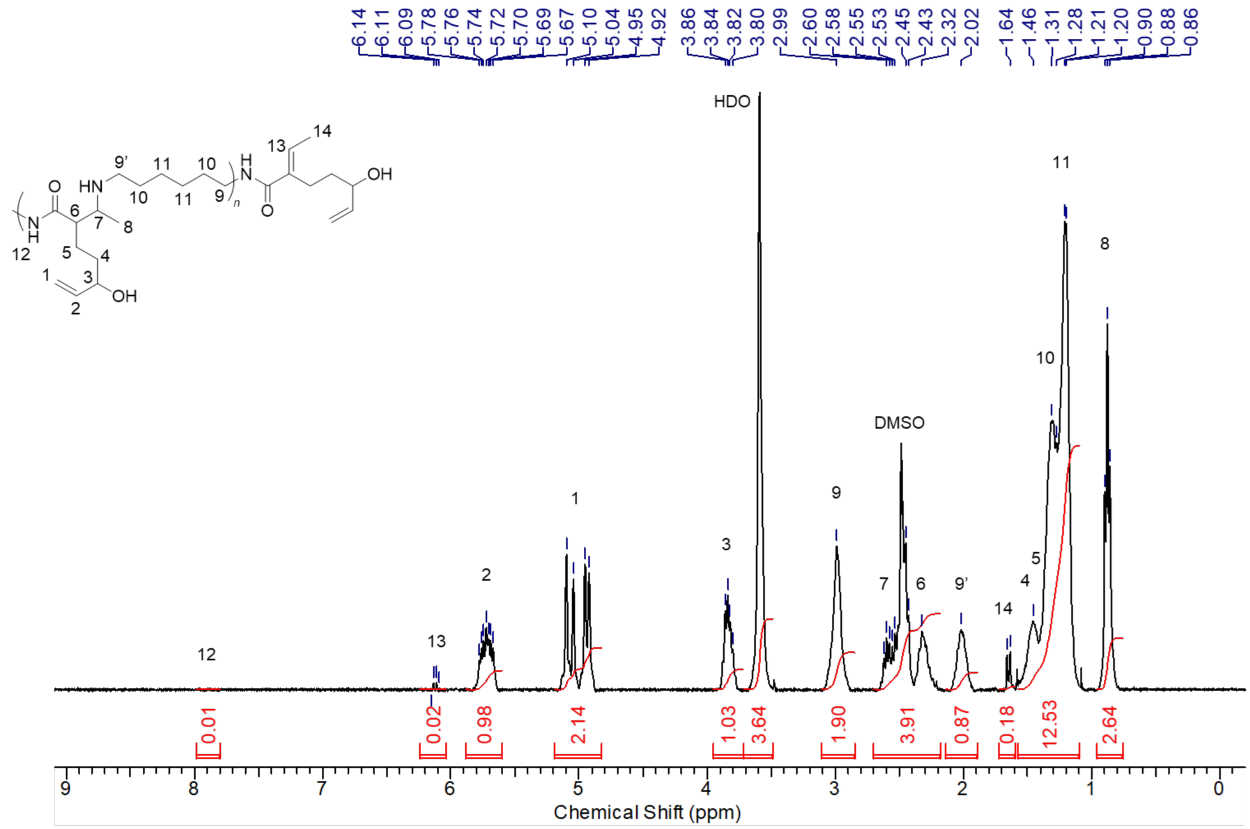

Fig. S26. ^{1}H NMR (500 MHz, DMSO-d_6, doped with D_2O) of poly(**1**/1,6-hexanediamine).

^{13}C NMR of Poly(amidoamine)

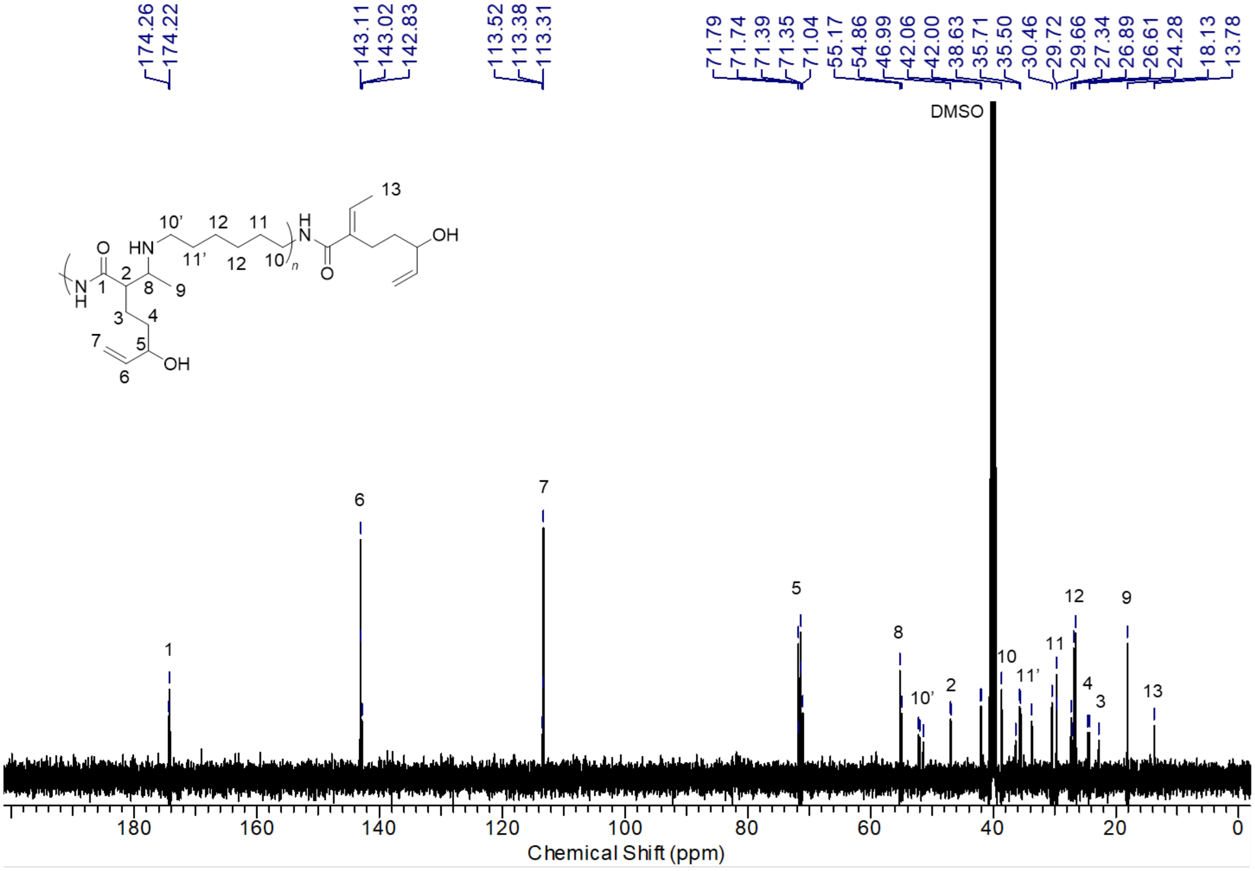

Fig. S27. ^{13}C NMR (125 MHz, DMSO-d_6) of poly(**1**/1,6-hexanediamine).

2D NMR of Poly(amidoamine)

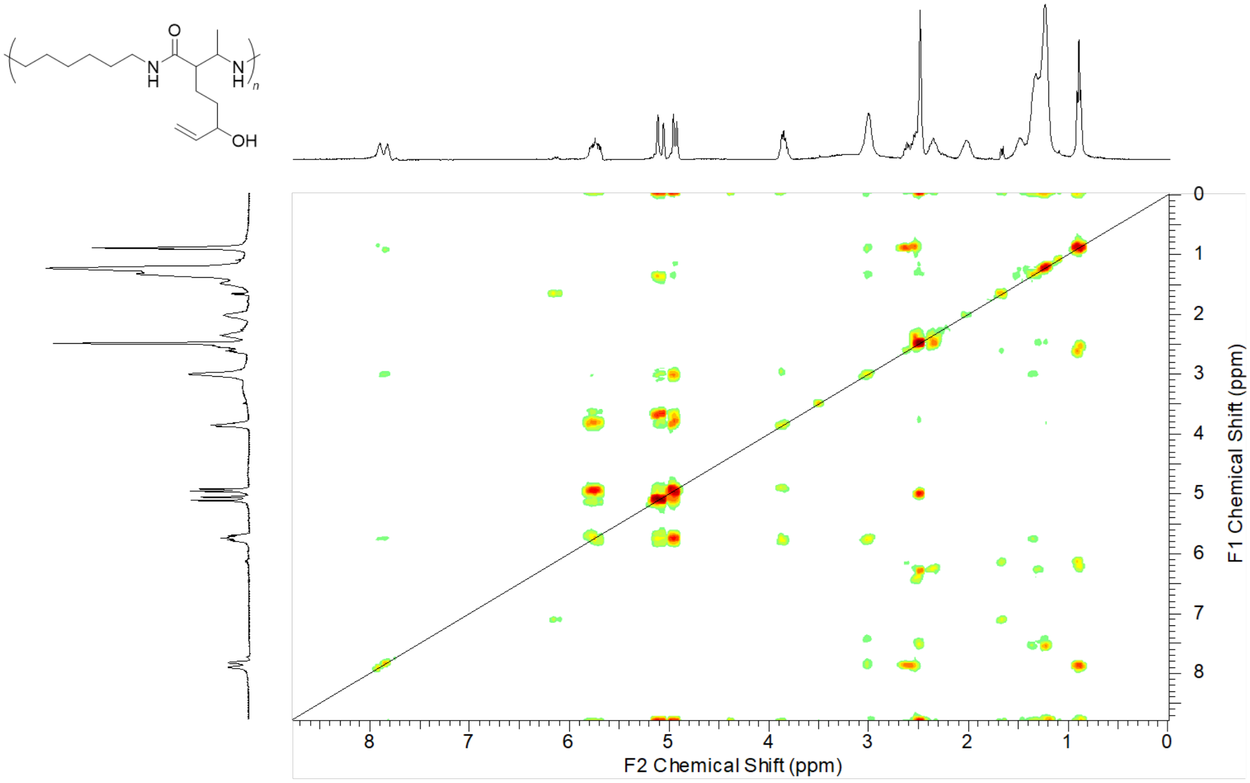

Fig. S28. COSY NMR (500 MHz, DMSO-d_6) of poly(**1**/1,6-hexanediamine).

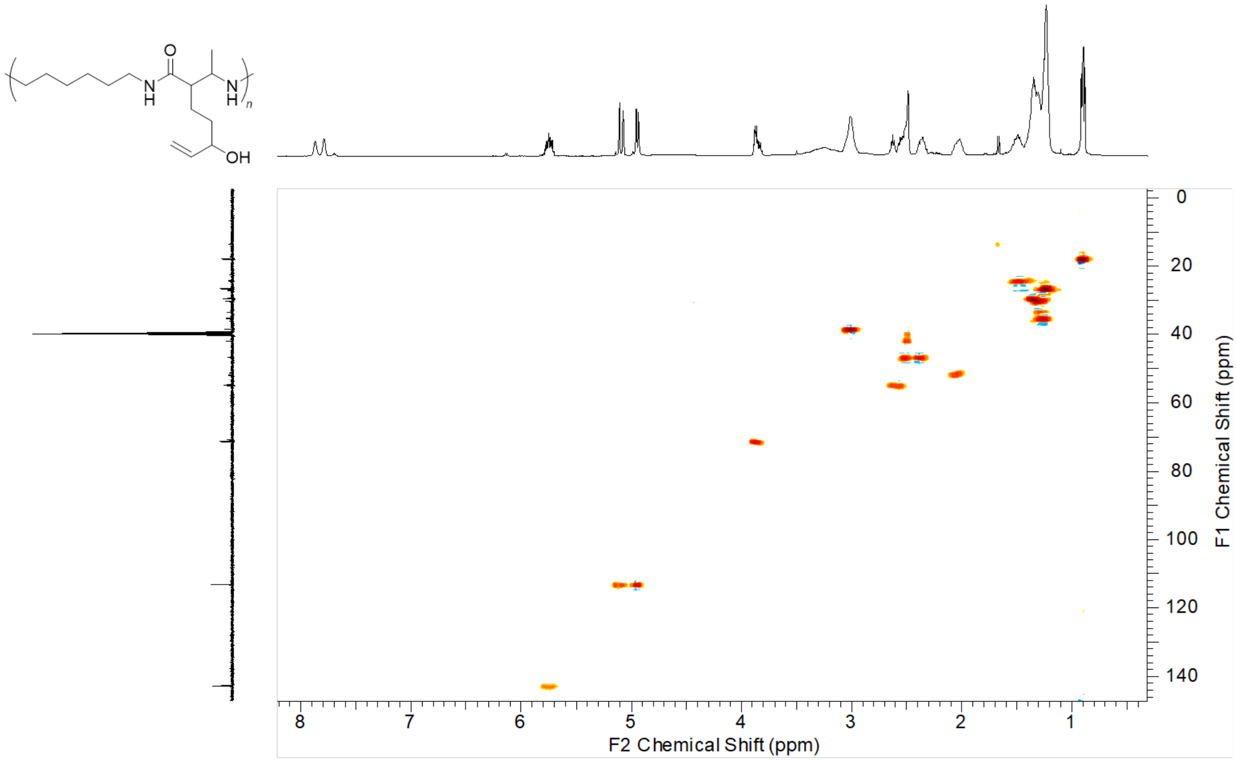

Fig. S29. HSQC (500 MHz, 125 MHz, DMSO-d_6) of poly(**1**/1,6-hexanediamine).

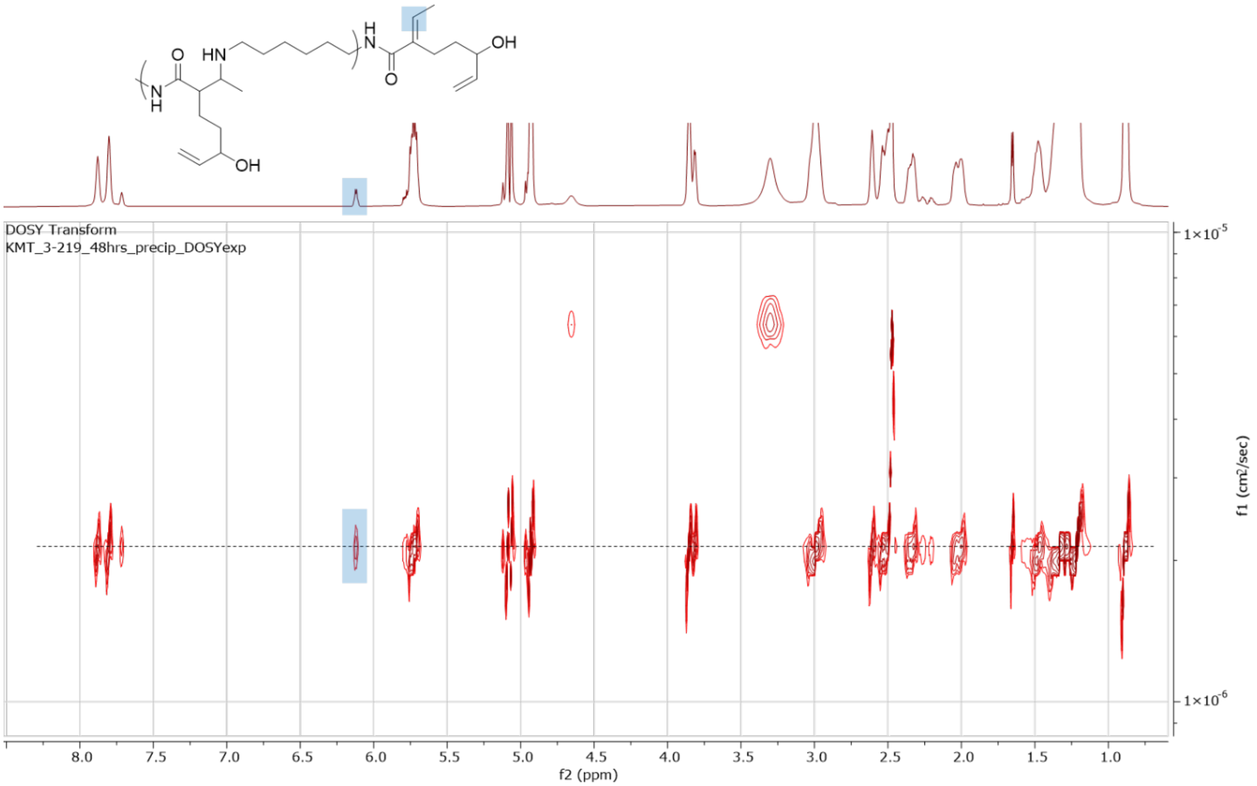

Fig. S30. Diffusion Order Spectroscopy (DOSY) NMR (750 MHz, DMSO-d_6) of poly(**1**/1,6-hexanediamine). DOSY analysis was used to address the claim that the tiglamide moiety is chemically incorporated into the polymer as opposed to being a separate lower molecular weight species. Confirmation of incorporation and lack of reactivity of the tiglamide species suggest this is a plausible termination mechanism during polymerization.

Screening Conditions of Poly(amidoamines)

Table S2. Screening of polymerization conditions.

Entry	Time (hours)	Temperature (°C)	Solvent[a]	Catalyst[b]	M_n (kDa)[c]	*Đ*[c]	T_g (°C)[d]	Yield (%)
0*	24	100	-	-	4.09 ± 1.2	1.9 ± 0.4	20.3 ± 5	86 ± 5
1	1	100	-	-	4.32	1.5	21.9	66
2	2	100	-	-	5.17	1.4	21.2	71
3	3	100	-	-	5.34	1.4	22.3	66
4	6	100	-	-	5.31	1.4	20.9	76
5	12	100	-	-	5.49	1.4	20.8	65
6	24	100	-	-	5.64	1.5	24.0	88
7	24	25	-	-	0.46	7.2	1.9	79
8	24	50	-	-	2.81	2.2	11.4	78
9	24	75	-	-	2.99	2.1	12.4	79
10	24	100	-	-	2.94	2.4	13.6	75
11	24	125	-	-	3.09	4.0	13.7	76
12	24	150	-	-	2.28	2.9	7.0	57
13	24	175	-	-	-	-	-	-
14	24	25	CH_2Cl_2	-	2.76	1.9	11.1	61
15	24	25	PhMe	-	2.88	1.8	9.9	53
16	24	25	MeCN	-	2.72	1.9	12.0	52
17	24	25	Hexanes	-	3.34	1.6	12.1	67
18	24	25	MeOH	-	2.39	1.8	11.9	28
19	24	25	DMF	-	1.16	3.4	15.2	26
20	24	100	-	$ZnCl_2$	3.26	2.5	24.0	84
21	24	100	-	$MgCl_2$	1.95	3.1	24.3	78
22	24	100	-	$Sn(Oct)_2$	2.49	3.6	21.4	70
23	24	100	-	DBU	1.96	2.9	8.5	67
24	24	100	-	TBD	2.90	2.1	14.5	71

Polymerizations conducted in a 1:1 molar ratio. *Representative sample under standardized conditions. [a]Solvent screening conducted at a concentration of 1M with respect to lactone **1**. [b]Catalyst screening conducted at 10 mol % catalyst loading. [c]Molecular weights and dispersity recorded using *N*,*N*-Dimethylformamide with 10 mM LiBr as a solvent at 40°C relative to polystyrene standards. [d]Determined by DSC using the second heat cycle with a heating rate of 10°C/minute.

GPCs of Condition Screening for Poly(amidoamines)

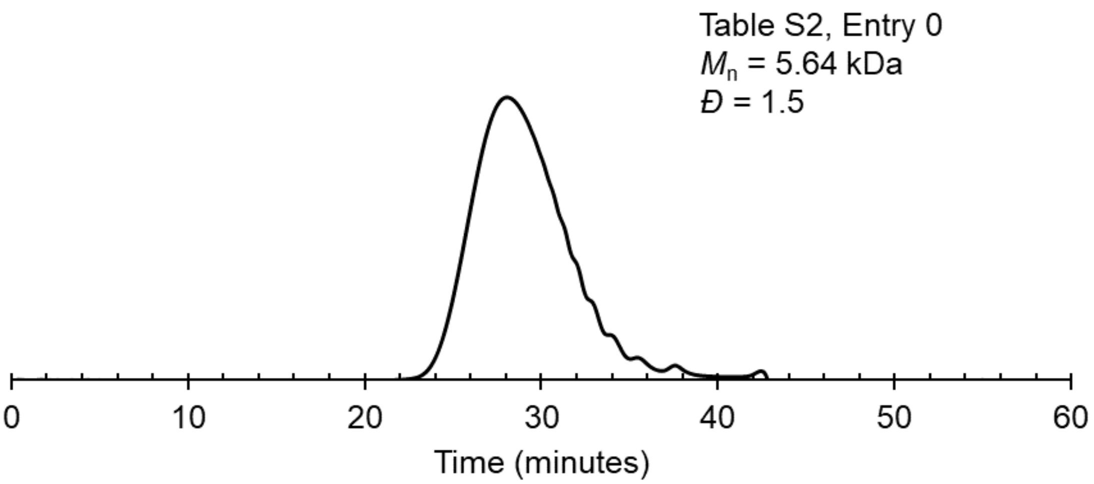

Fig. S31. GPC of poly(**1**/1,6-hexanediamine) (Table S2, Entry 0).

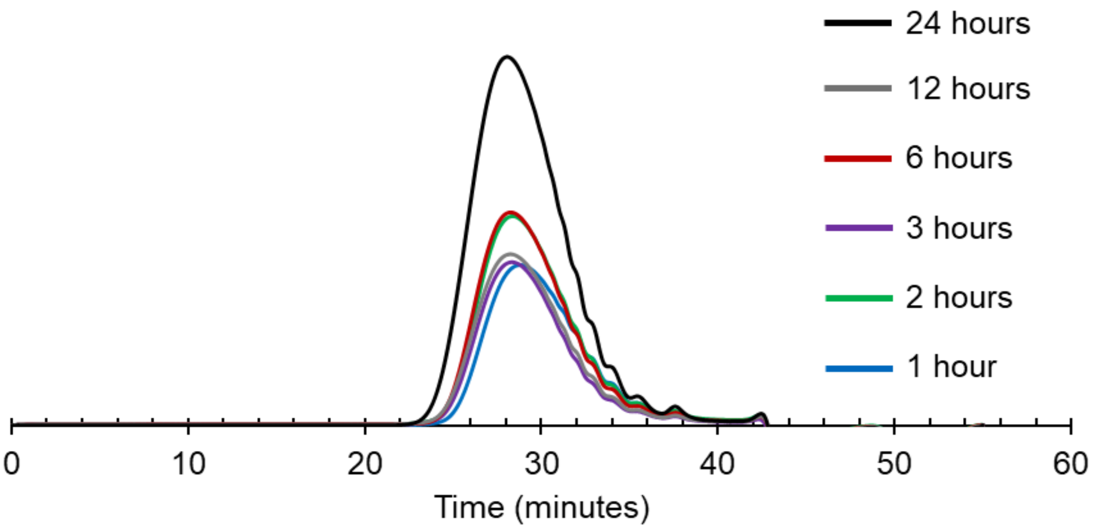

Fig. S32. GPC of the time screen for poly(**1**/1,6-hexanediamine) polymerization (Table S2, Entries 1–6).

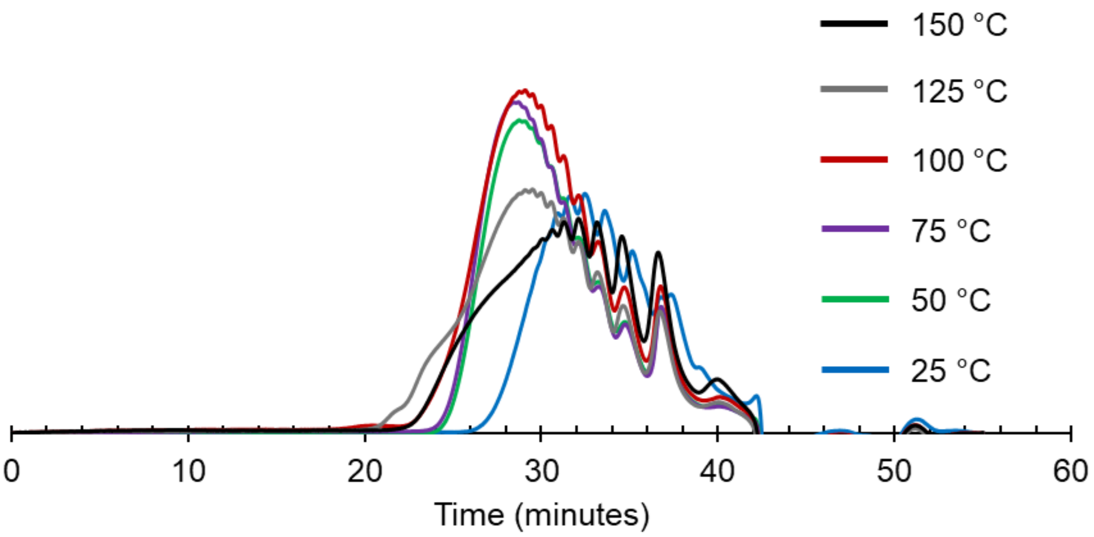

Fig. S33. GPC of the temperature screen for poly(**1**/1,6-hexanediamine) polymerization (Table S2, Entries 7–12).

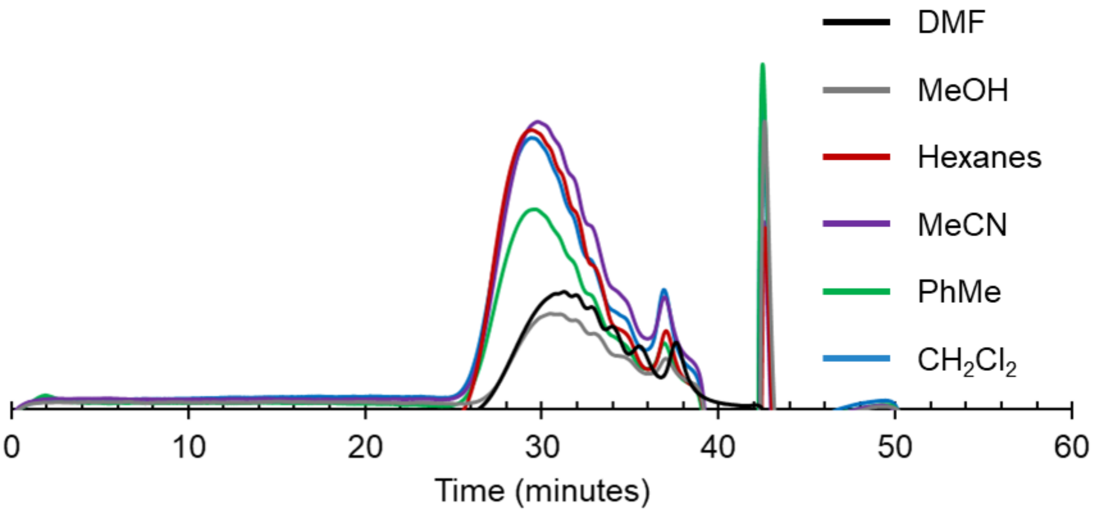

Fig. S34. GPC of the solvent screen for poly(**1**/1,6-hexanediamine) polymerization (Table S2, Entries 14–19).

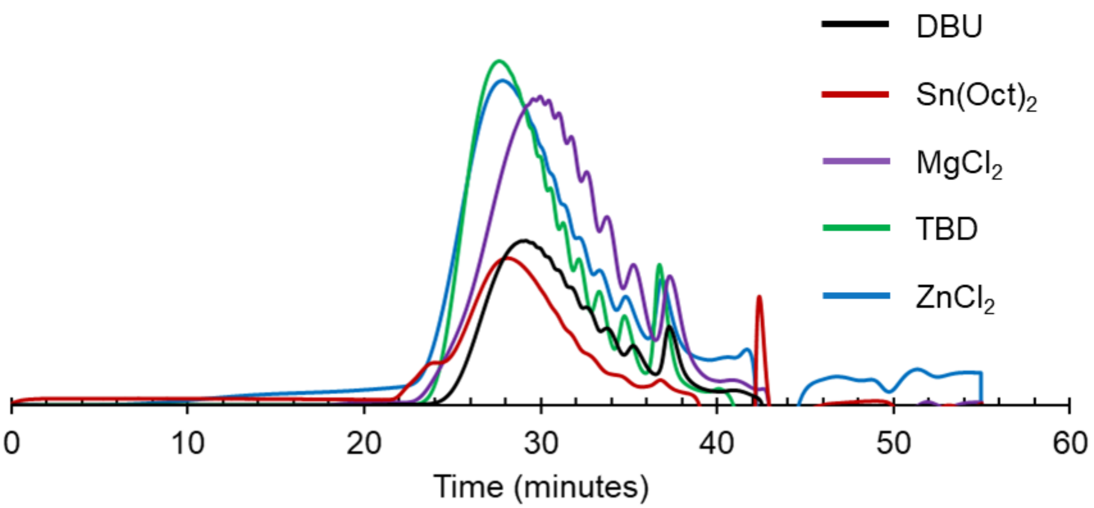

Fig. S35. GPC of the catalyst screen for poly(**1**/1,6-hexanediamine) polymerization (Table S2, Entries 20–24).

DSCs of Condition Screening for Poly(amidoamines)

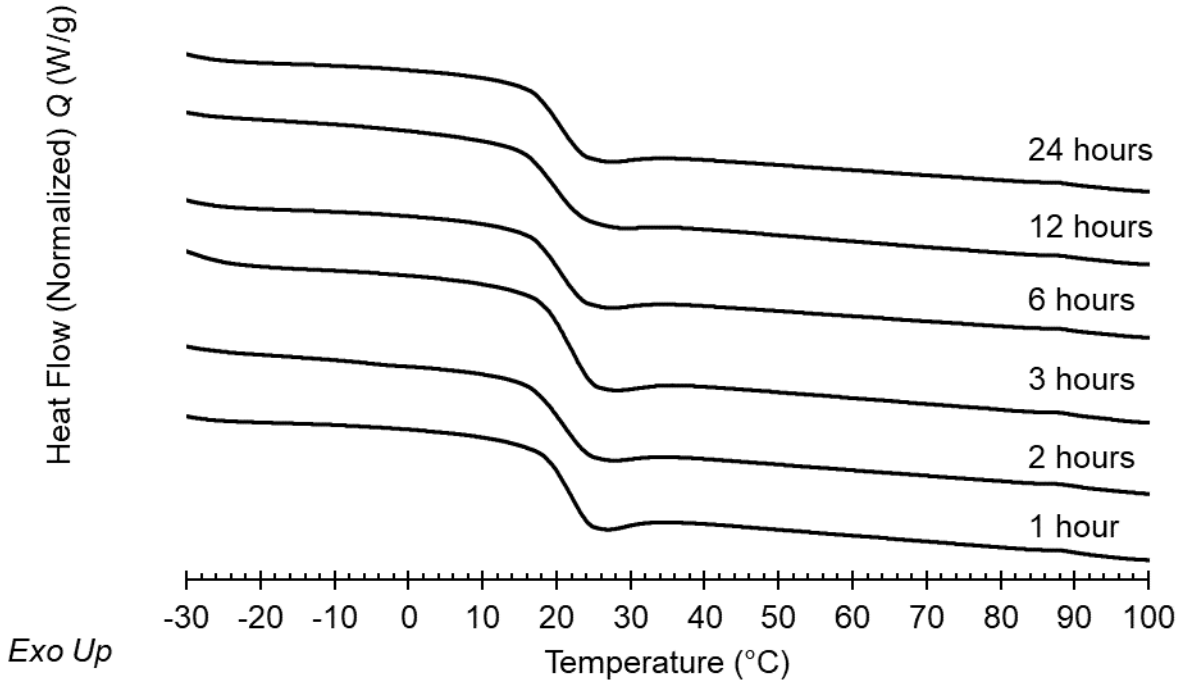

Fig. S36. DSC of the time screen for poly(**1**/1,6-hexanediamine) performed at 10°C/minute (Table S2, Entries 1–6).

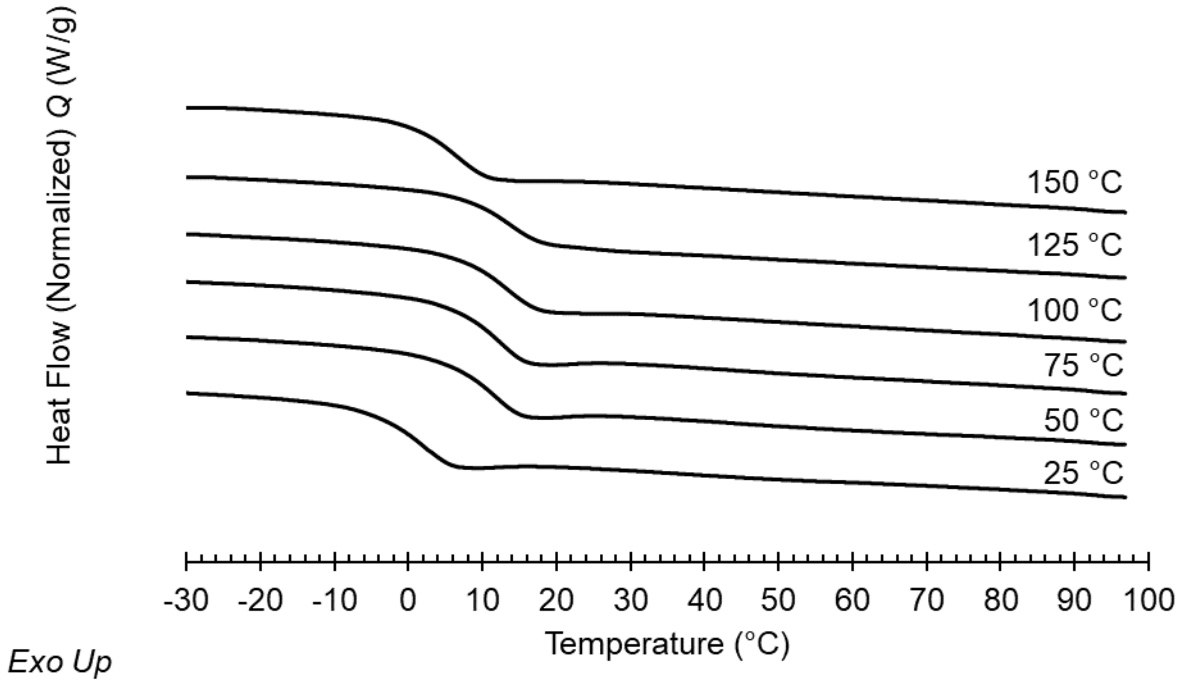

Fig. S37. DSC of the temperature screen for poly(**1**/1,6-hexanediamine) performed at 10°C/minute (Table S2, Entries 7–12).

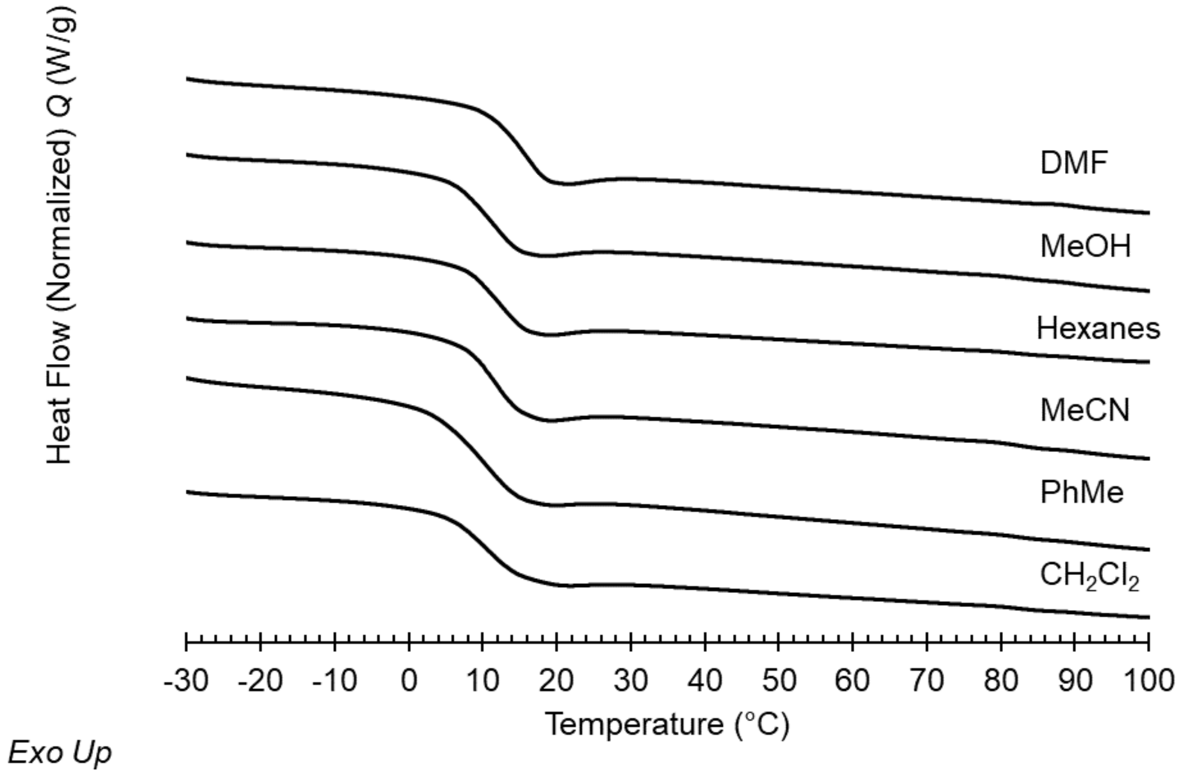

Fig. S38. DSC of the solvent screen for poly(**1**/1,6-hexanediamine) performed at 10°C/minute (Table S2, Entries 14–19).

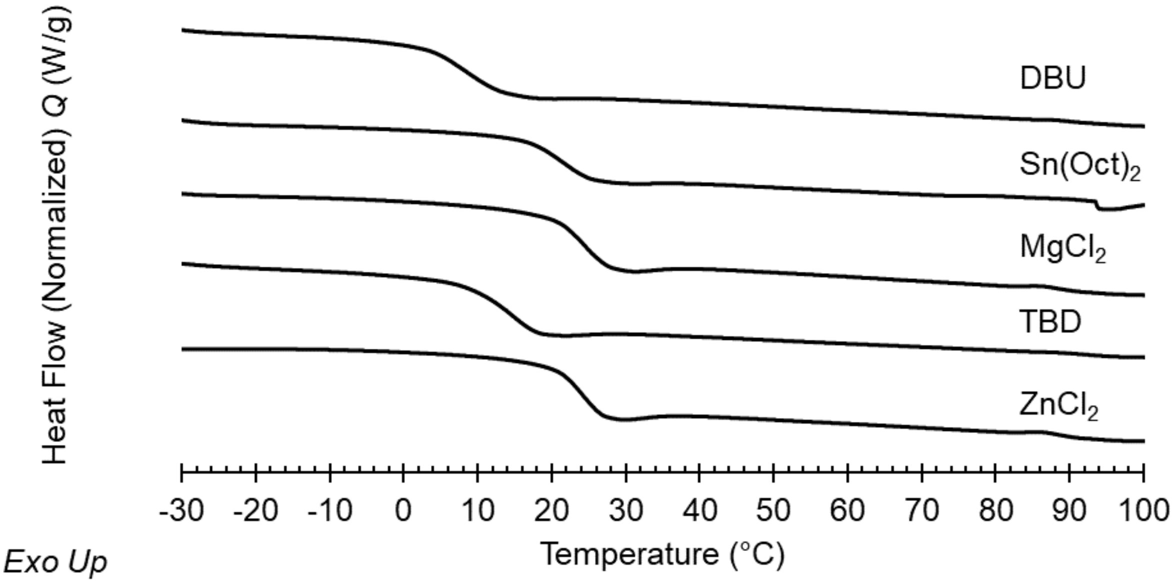

Fig. S39. DSC of the catalyst screen for poly(**1**/1,6-hexanediamine) performed at 10°C/minute (Table S2, Entries 20–24).

Screening Diamine Species for Poly(amidoamines)

Table S3. Screening of diamine species.

Entry	Difunctional Comonomer	M_n (kDa)[a]	$Đ$[a]	T_g (°C)[b]	Yield (%)
0*	1,6-Hexanediamine	5.64	1.5	24.0	88
1[c]	Jeffamine D230	1.50	3.3	-29.5	89
2	Bis(Hexamethylene)triamine	3.01	2.1	1.7	71
3	N,N'-Bis(3-aminopropyl)ethylenediamine	0.30	2.3	5.8	77
4	Diethylaminetriamine	0.60	2.3	18.6	87
5	1,5-Pentanediamine	3.54	1.9	26.5	85
6	*m*-Xylylenediamine	2.53	1.7	32.9	80
7[d]	1,2-Ethylenediamine	1.36	2.7	36.7	50
8	*p*-Xylylenediamine	2.29	2.0	51.8	83
9	Isophoronediamine	1.99	1.9	84.3	22
10	*trans*-1,4-Cyclohexanediamine	2.44	2.0	94.8	77

Polymerizations conducted in a 1:1 molar ratio. Temperature of reaction was 100°C. Duration of reaction was 24 hours. *Representative sample under standardized conditions. [a]Molecular weights and dispersity recorded using *N*,*N*-Dimethylformamide with 10 mM LiBr as a solvent at 40°C relative to polystyrene standards. [b]Determined by DSC using the second heat cycle with a heating rate of 10°C/minute. [c]Polymer soluble in Et_2O during workup, requiring the crude material to be analyzed. [d]Reaction performed at 70°C to remain below the boiling point of amine species. Information regarding the comonomer TREN was included later with matrix investigations.

^{1}H NMR of Diamine Screening for Poly(amidoamines)

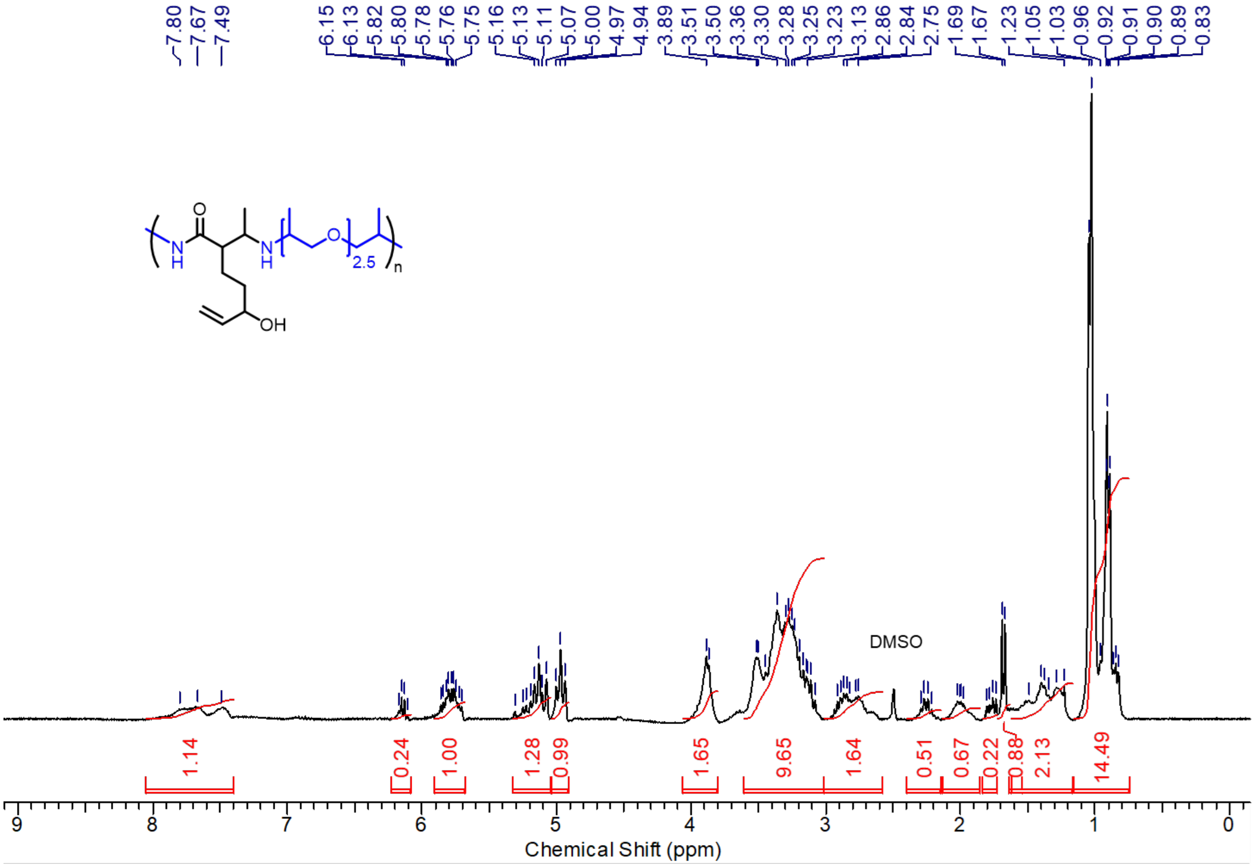

Fig. S40. ^{1}H NMR (300 MHz, DMSO-d_6) of **1**/Jeffamine 230 copolymer (Table S3, Entry 1).

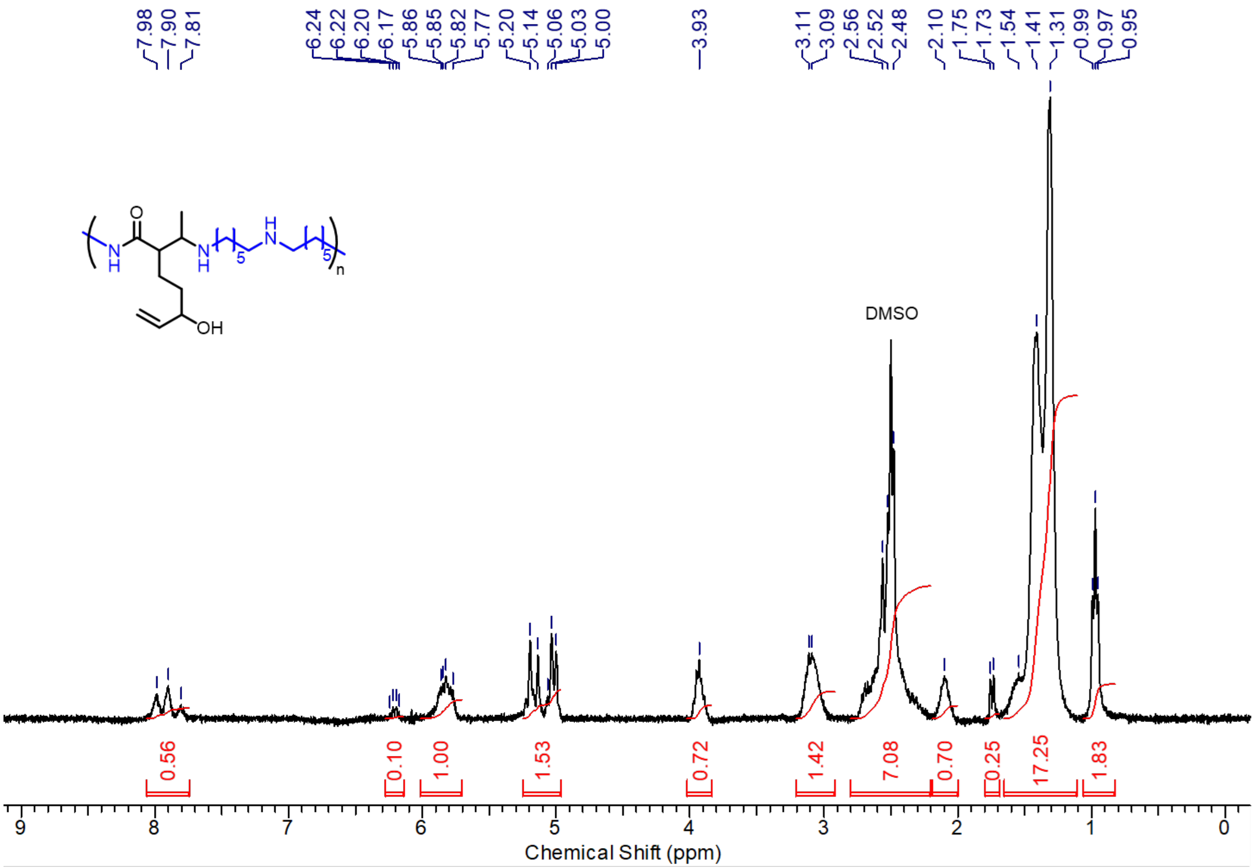

Fig. S41. ^{1}H NMR (300 MHz, DMSO-d_6) of **1**/Bis(Hexamethylene)triamine copolymer (Table S3, Entry 2).

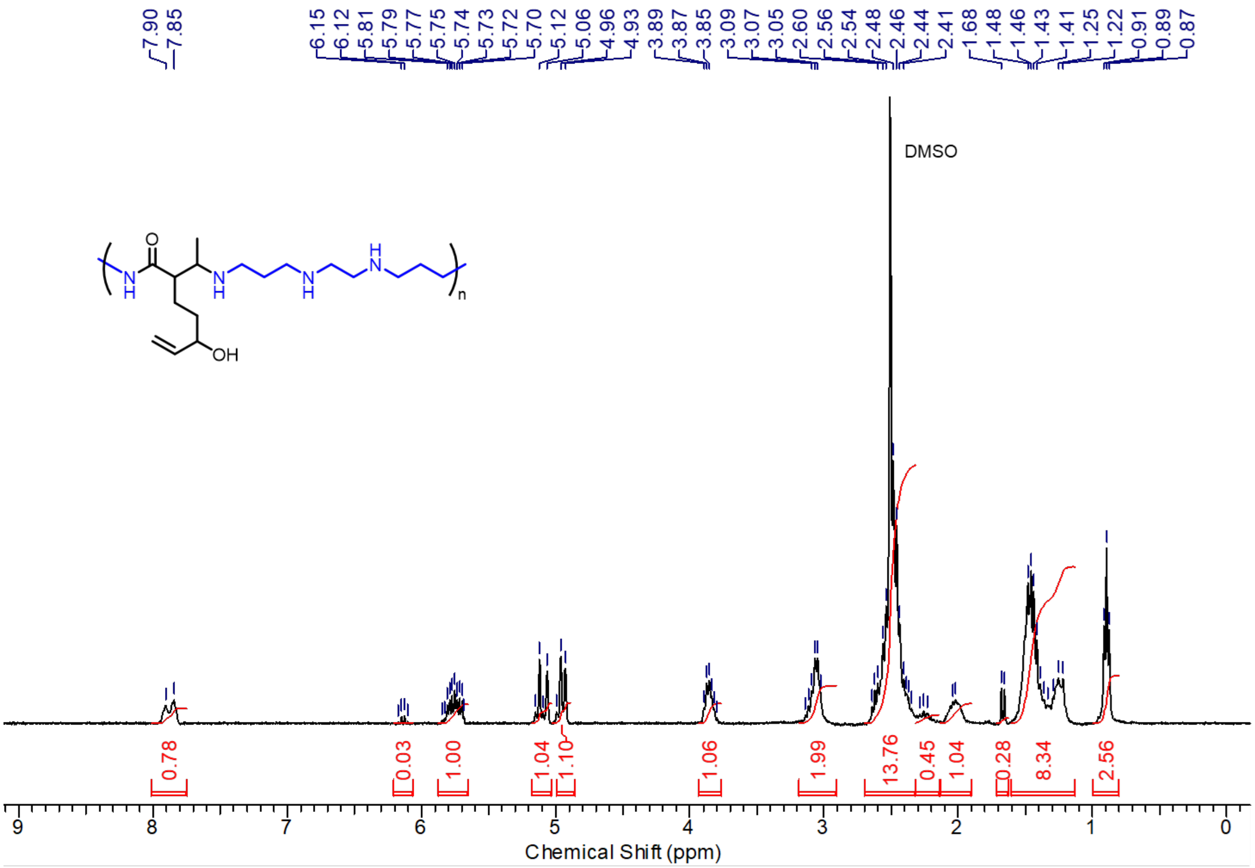

Fig. S42. ^{1}H NMR (300 MHz, DMSO-d_6) of **1**/N,N'-Bis(3-aminopropyl)ethylenediamine (Table S3, Entry 3).

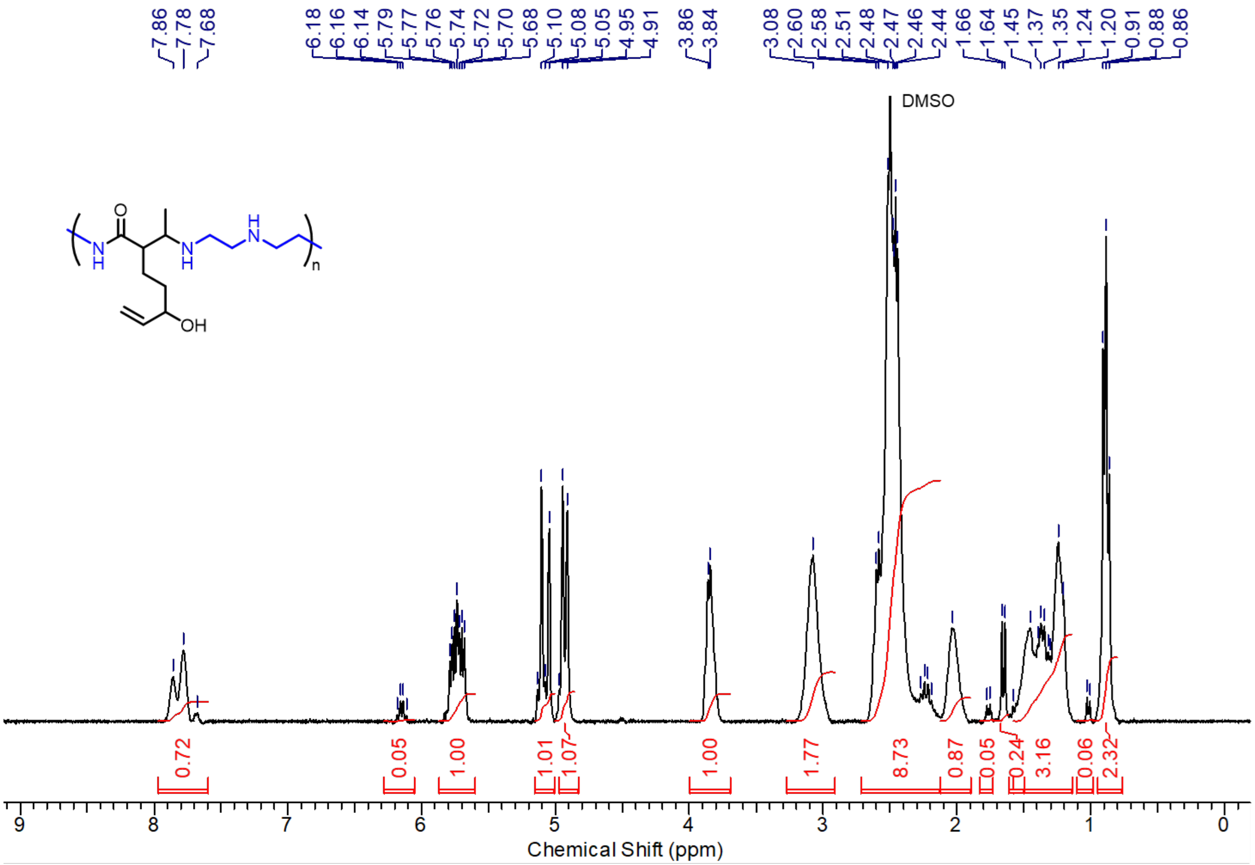

Fig. S43. ^{1}H NMR (300 MHz, DMSO-d_6) of **1**/Diethylaminetriamine copolymer (Table S3, Entry 4).

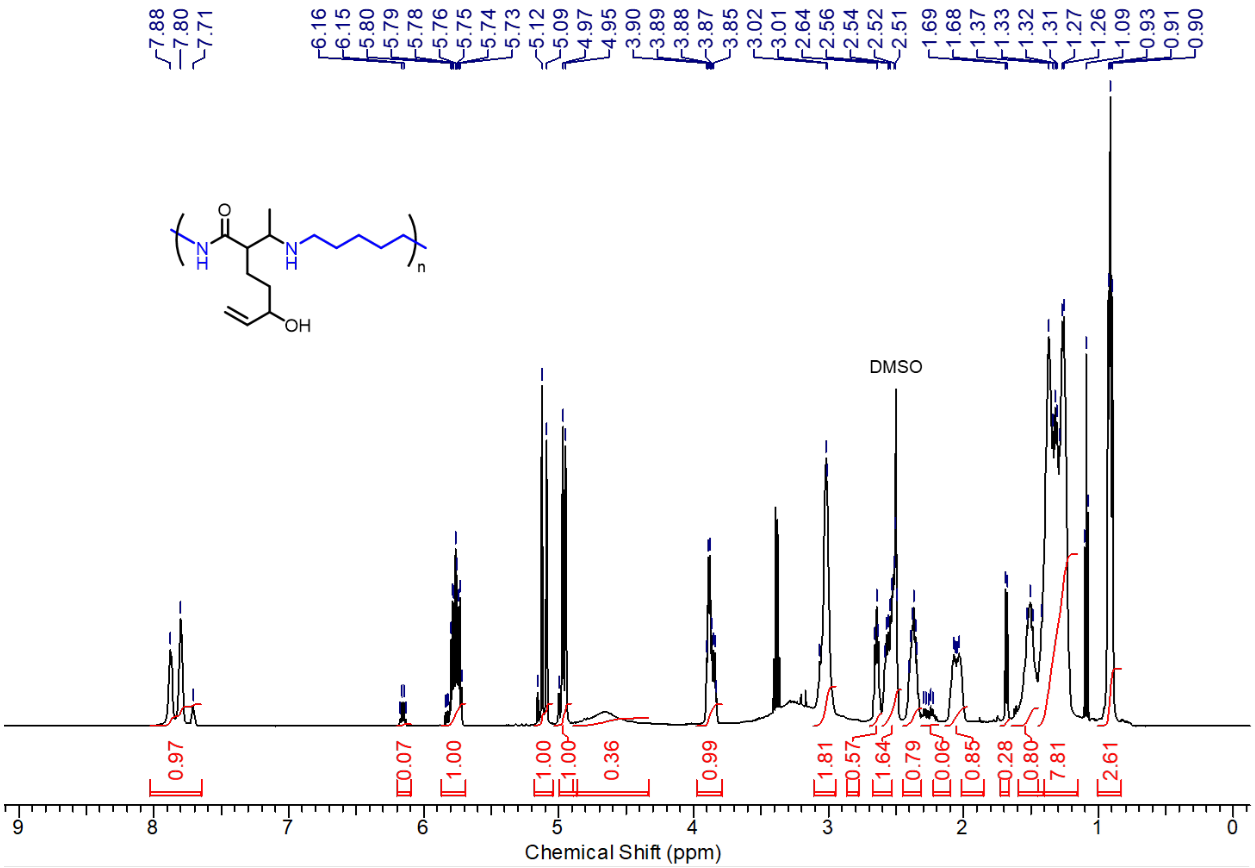

Fig. S44. ^{1}H NMR (500 MHz, DMSO-d_6) of **1**/1,5-Pentanediamine copolymer (Table S3, Entry 5).

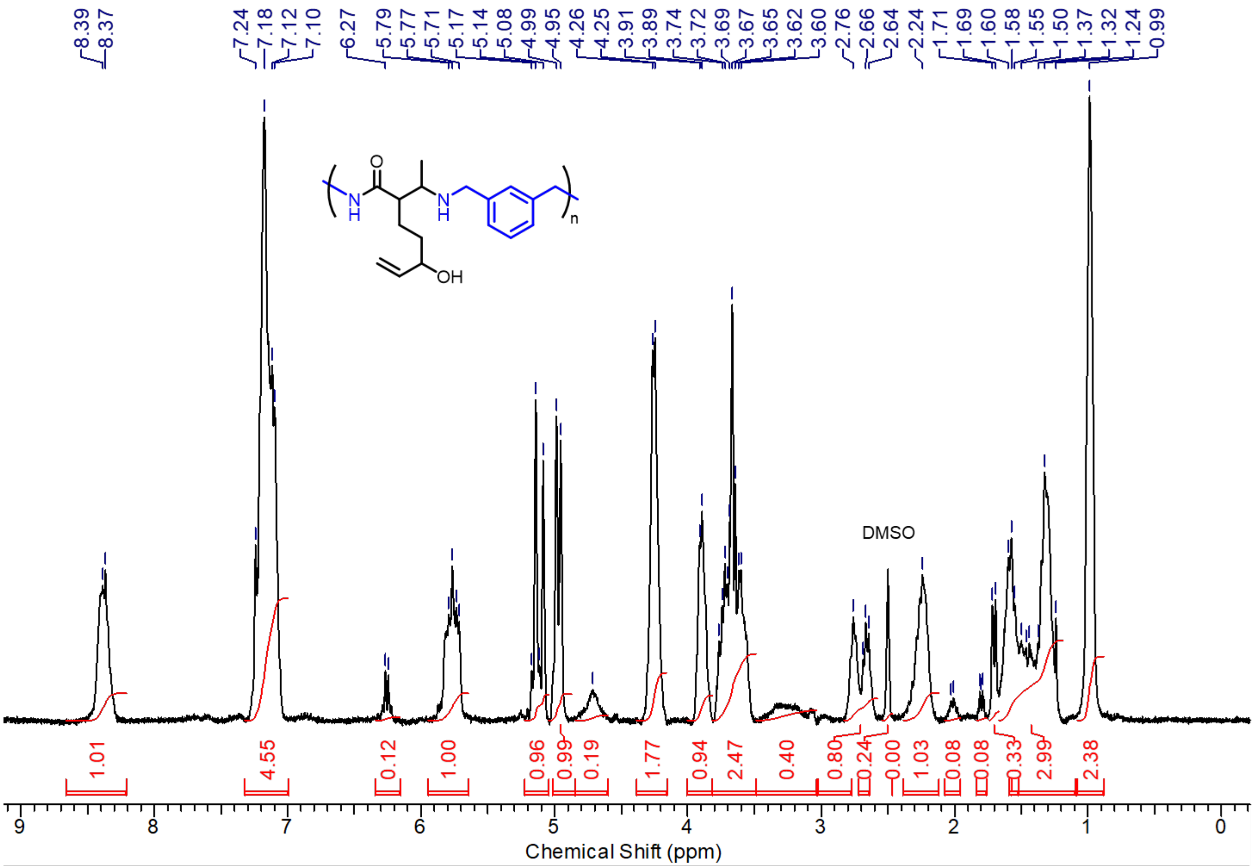

Fig. S45. ^{1}H NMR (300 MHz, DMSO-d_6) of **1**/*m*-Xylylenediamine copolymer (Table S3, Entry 6).

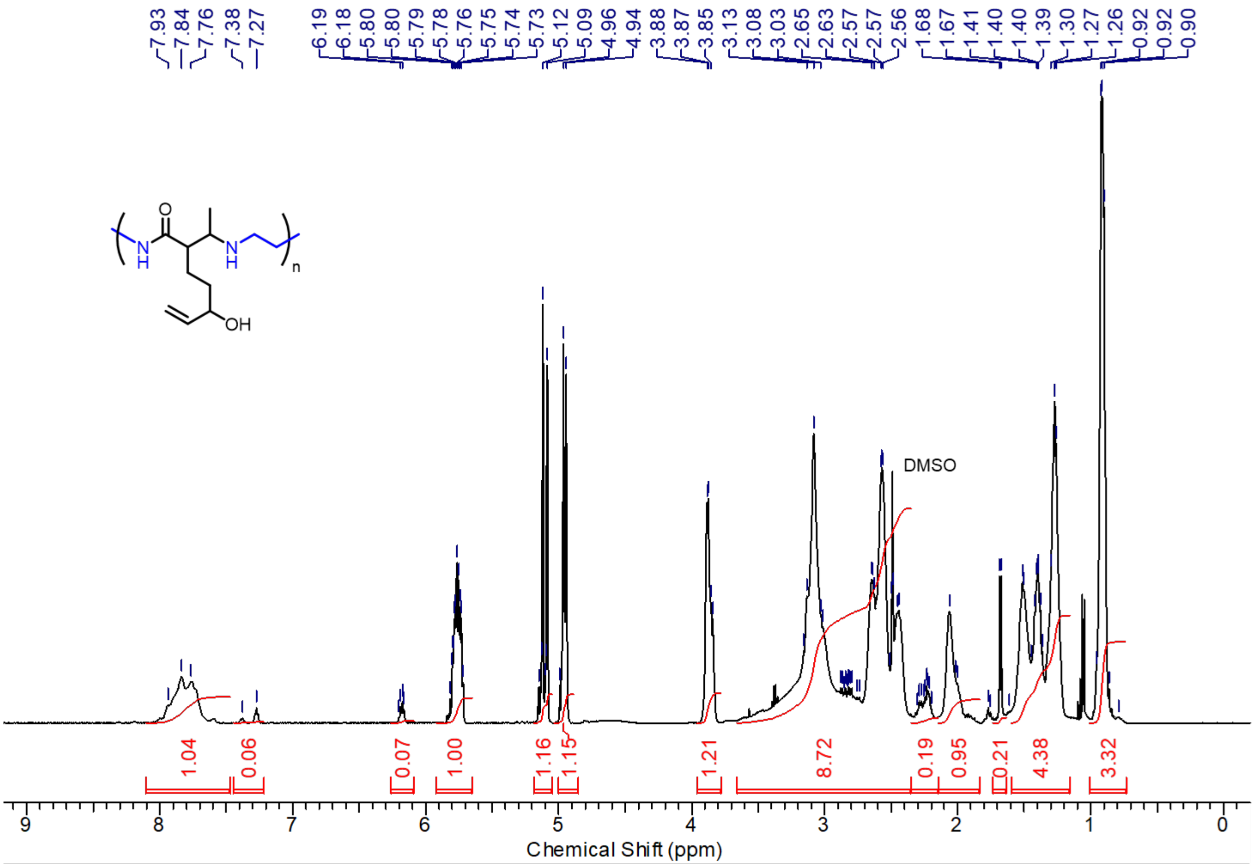

Fig. S46. ^{1}H NMR (500 MHz, DMSO-d_6) of **1**/1,2-Ethylenediamine copolymer (Table S3, Entry 7).

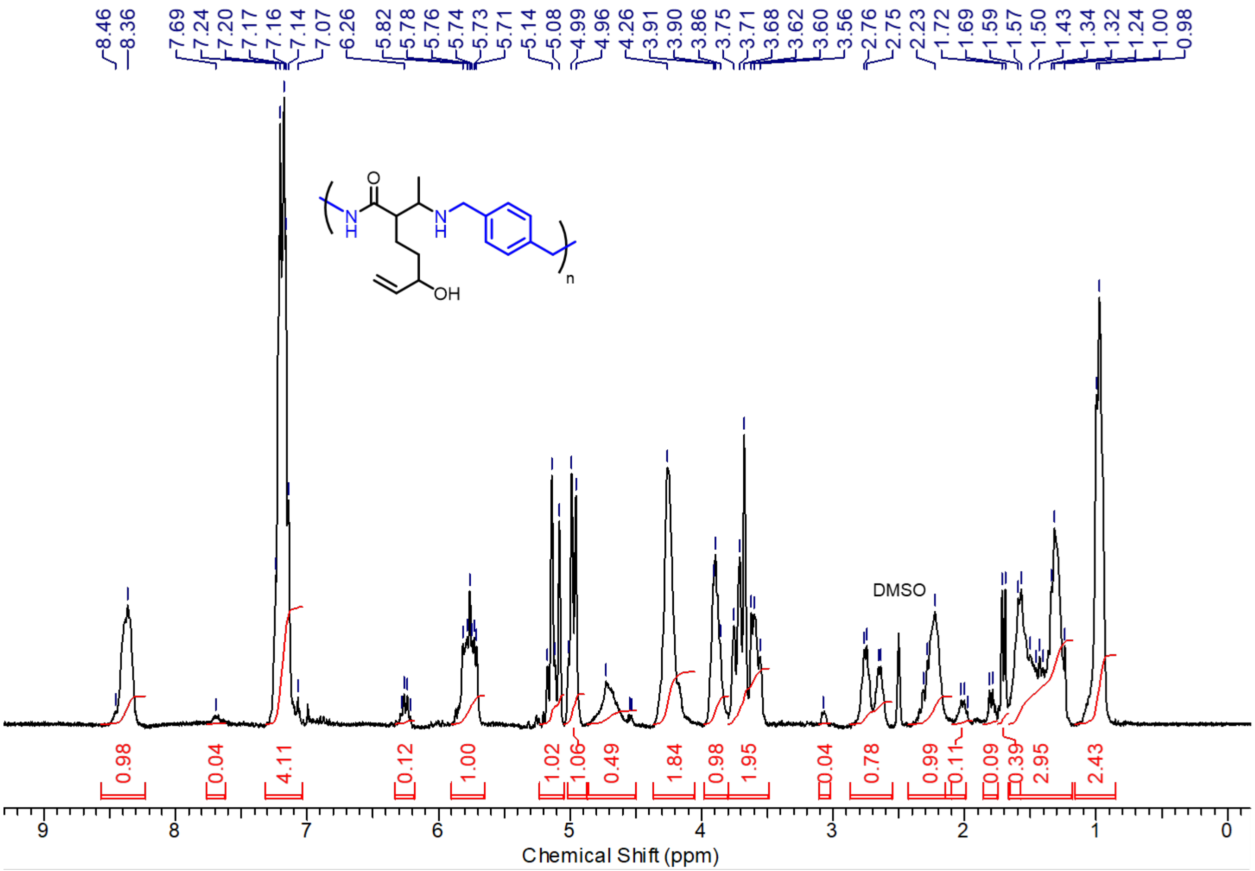

Fig. S47. ^{1}H NMR (300 MHz, DMSO-d_6) of **1**/*p*-Xylylenediamine copolymer (Table S3, Entry 8).

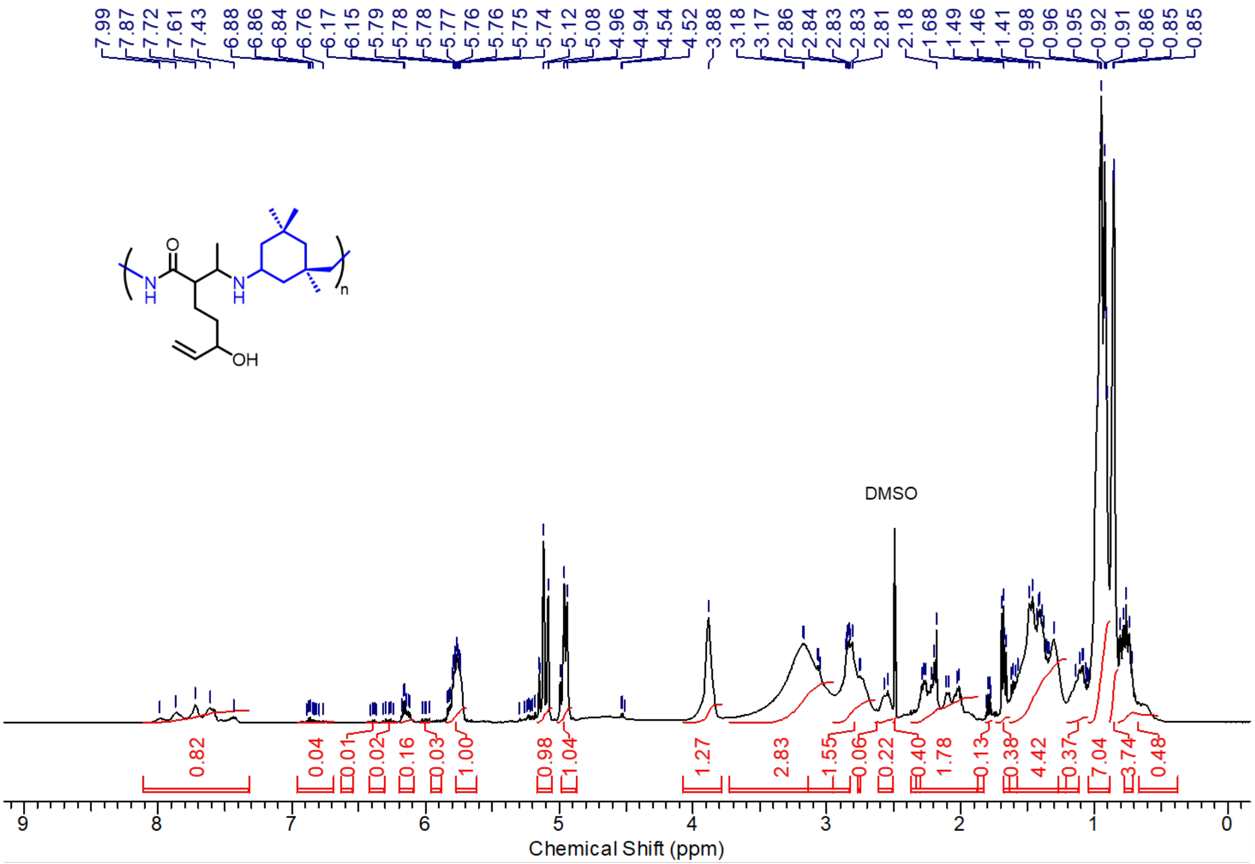

Fig. S48. ^{1}H NMR (500 MHz, DMSO-d_6) of **1**/Isophoronediamine copolymer (Table S3, Entry 9).

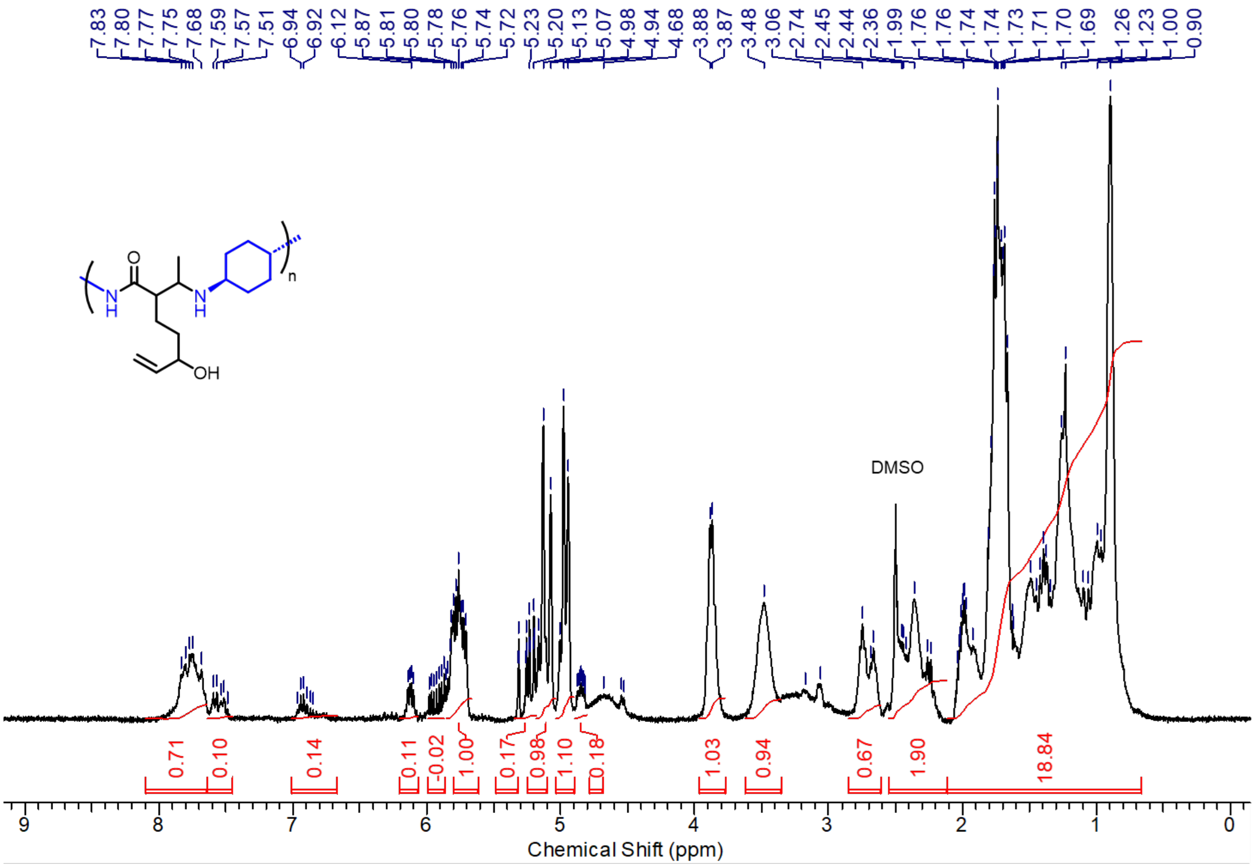

Fig. S49. ^{1}H NMR (300 MHz, DMSO-d_6) of **1**/*trans*-1,4-Cyclohexanediamine copolymer (Table S3, Entry 10).

GPCs of Diamine Screening for Poly(amidoamines)

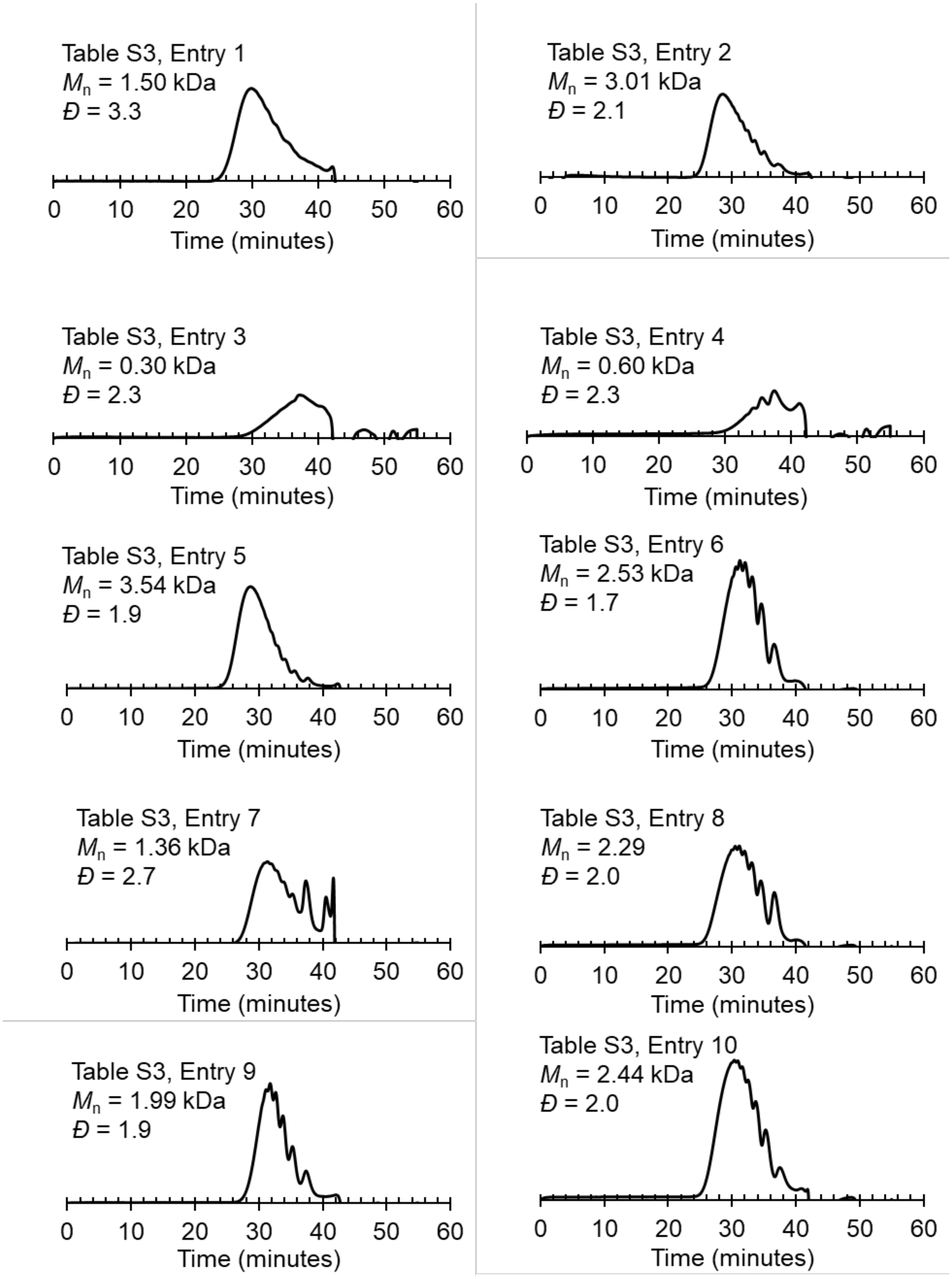

Fig. S50. GPCs of diamine screen for poly(amidoamines) from Table S3.

DSCs of Diamine Screening for Poly(amidoamines)

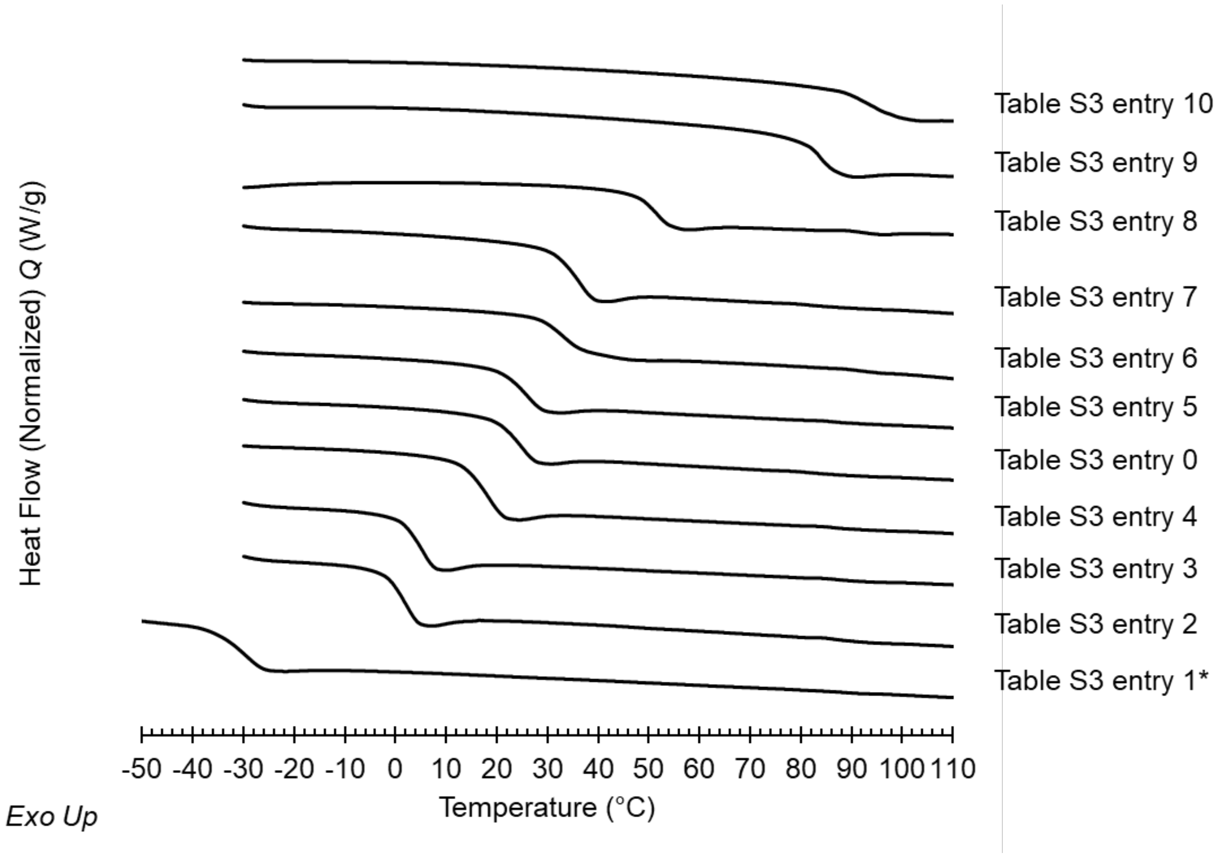

Fig. S51. DSCs of diamine screen for poly(amidoamines) performed at 10°C/minute from Table S3. *Lower bound of analysis window expanded to -60°C to accommodate lower T_g.

Mass Spectrometry of Poly(amidoamines)

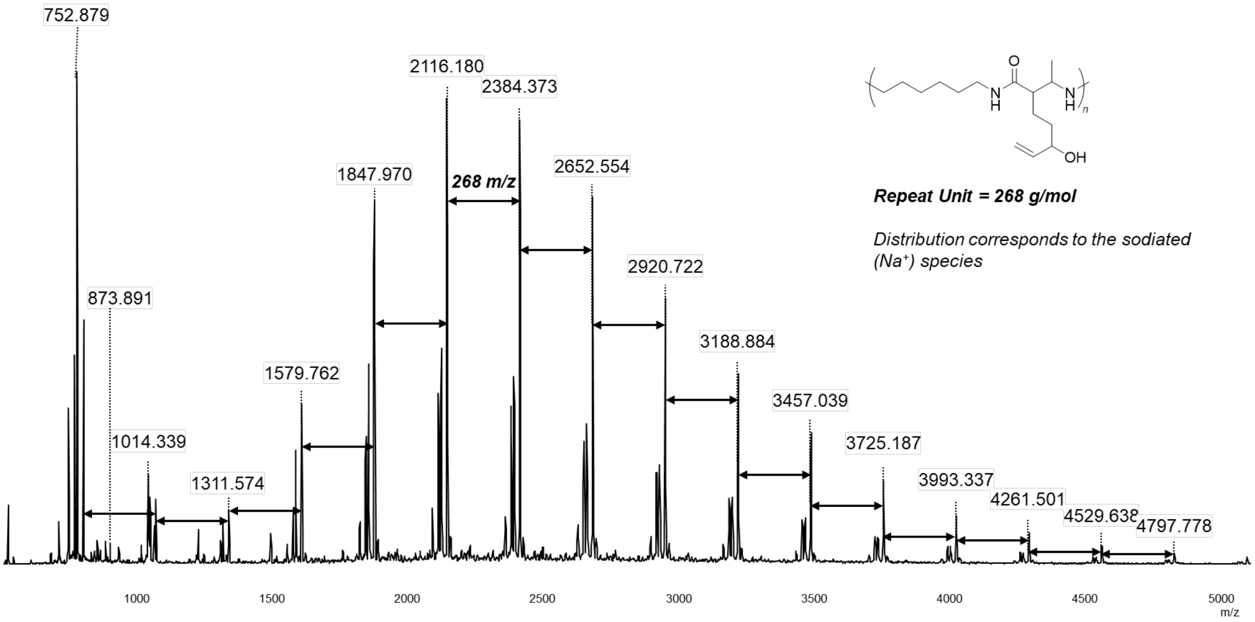

Fig. S52. Matrix-Assisted Laser Desorption/Ionization Mass Spectrometry (MALDI-MS) of poly(**1**/1,6-hexanediamine) (Table S3, Entry 0).

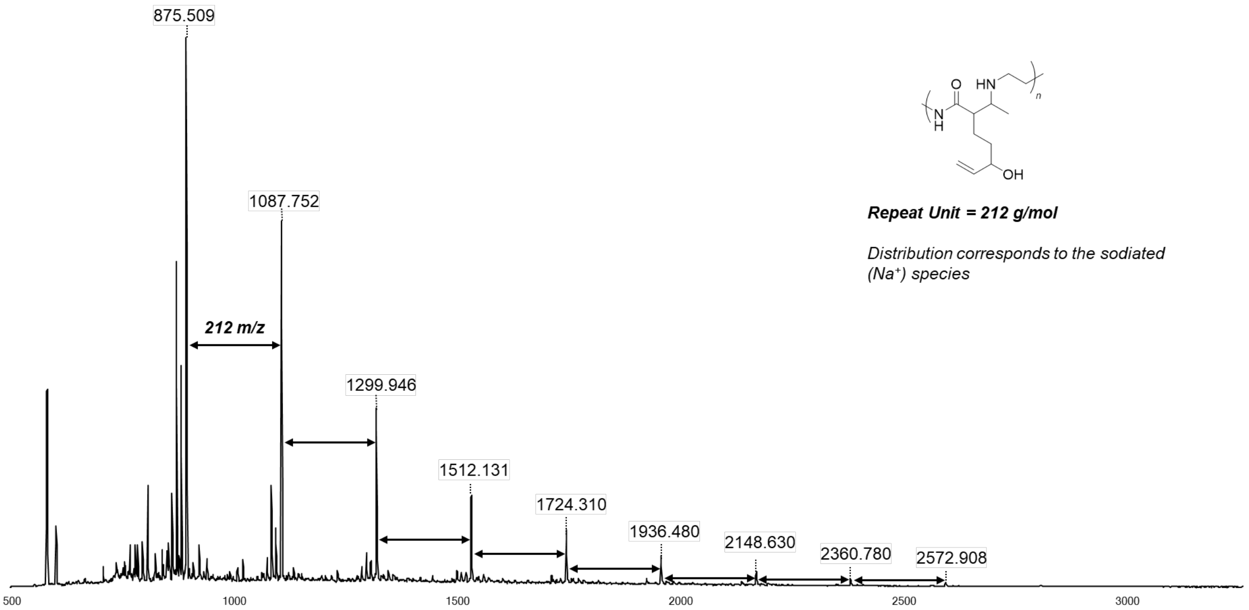

Fig. S53. MALDI-MS of poly(**1**/ethylenediamine) (Table S3, Entry 7).

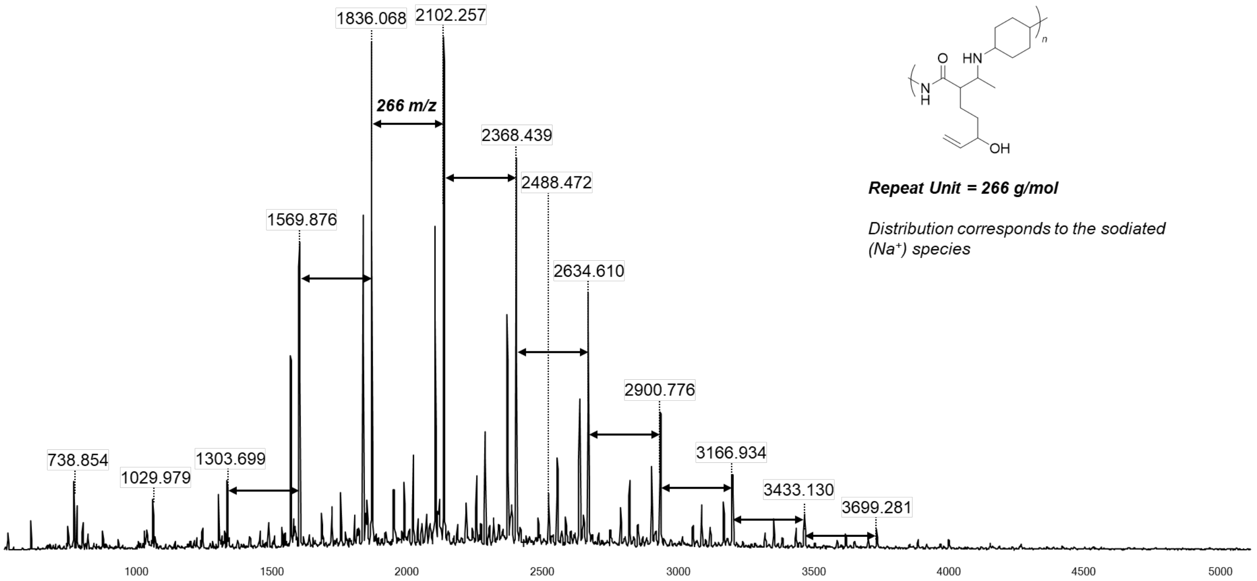

Fig. S54. MALDI-MS of poly(**1**/trans-1,4-diaminocyclohexane) (Table S3, Entry 10).

ATR-FTIR of Poly(amidoamine) and Matrices

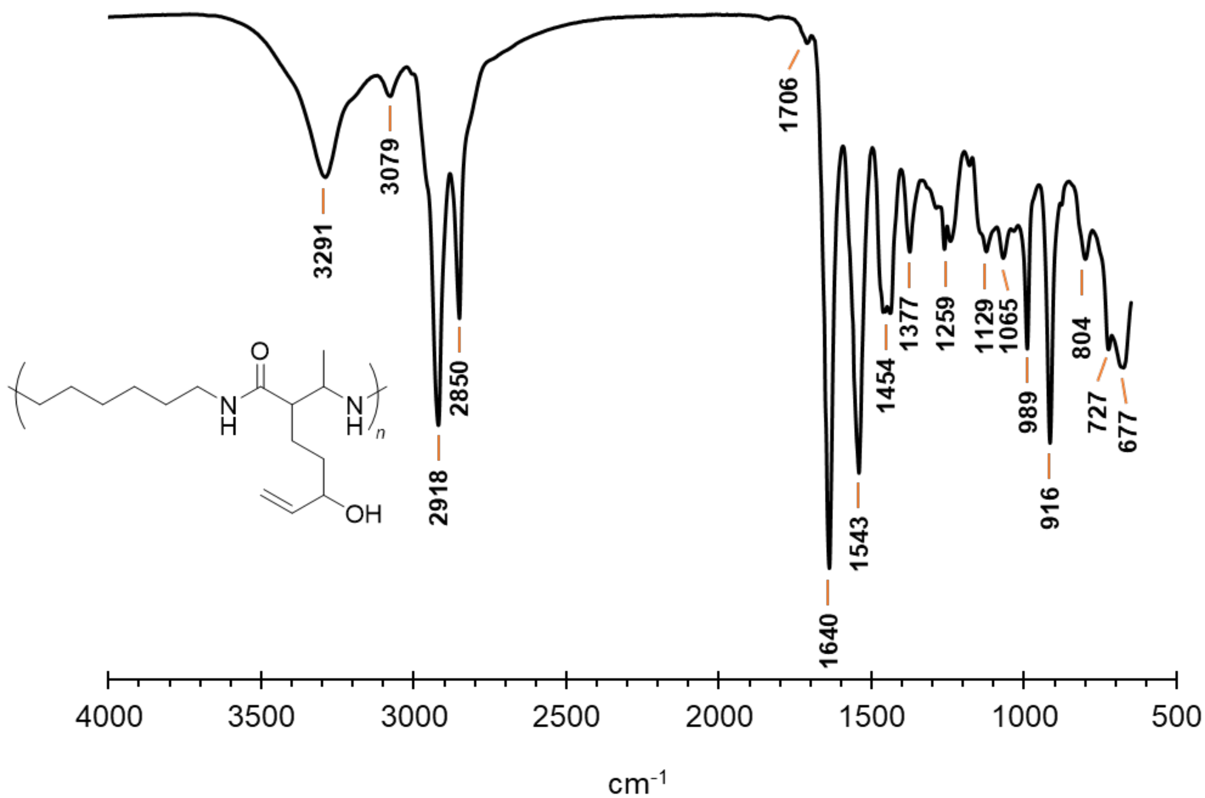

Fig. S55. ATR-FTIR of poly(**1**/1,6-hexanediamine).

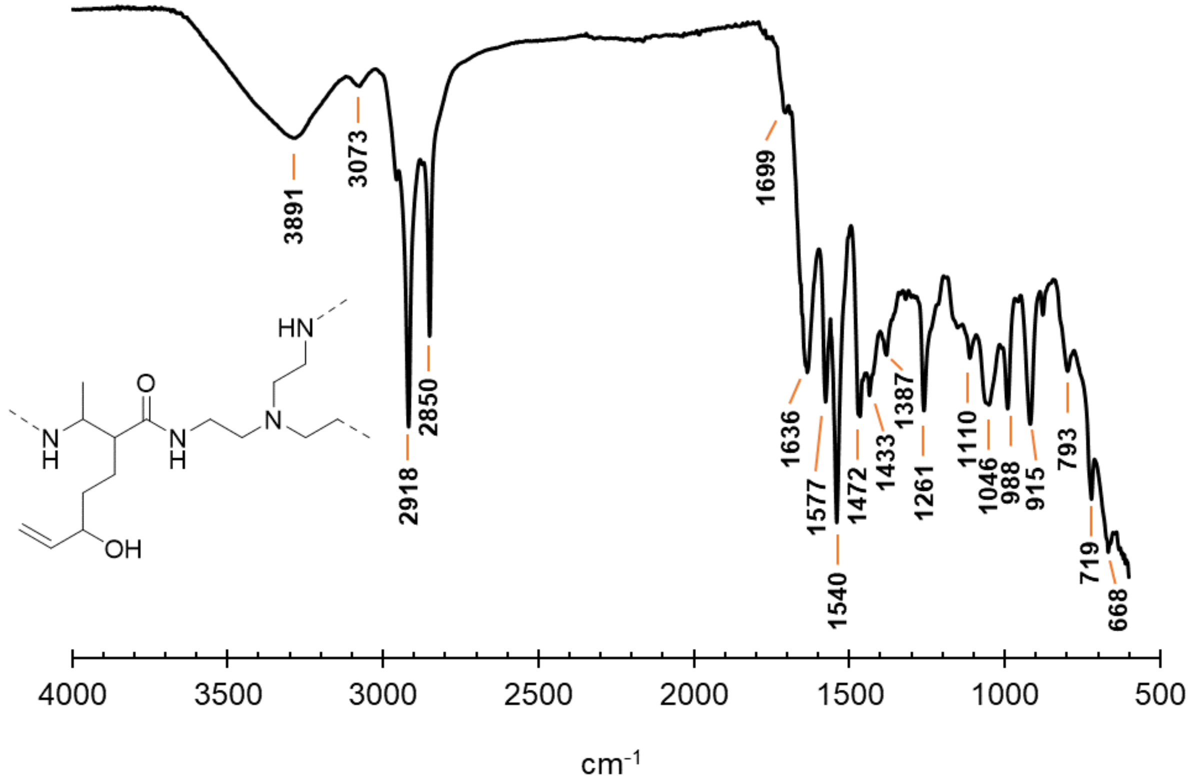

Fig. S56. ATR-FTIR of **1**/TREN matrix.

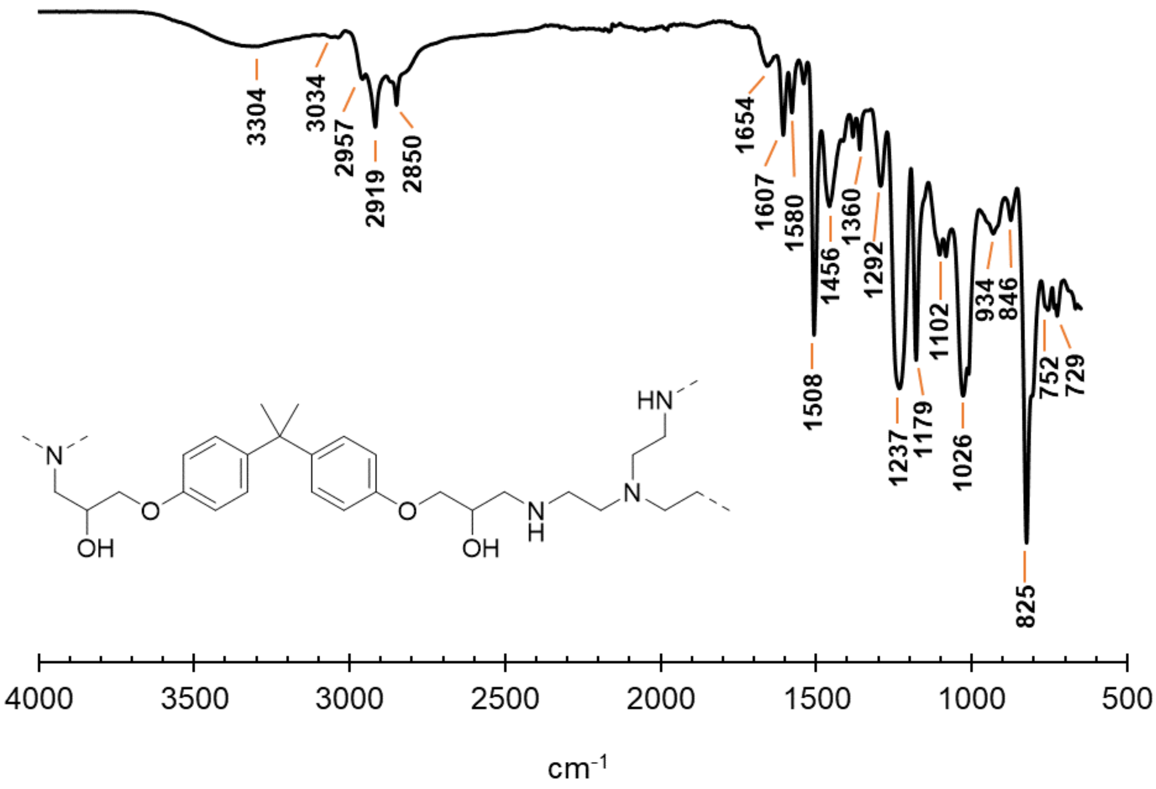

Fig. S57. ATR-FTIR of DGEBA/TREN matrix.

Rheometry

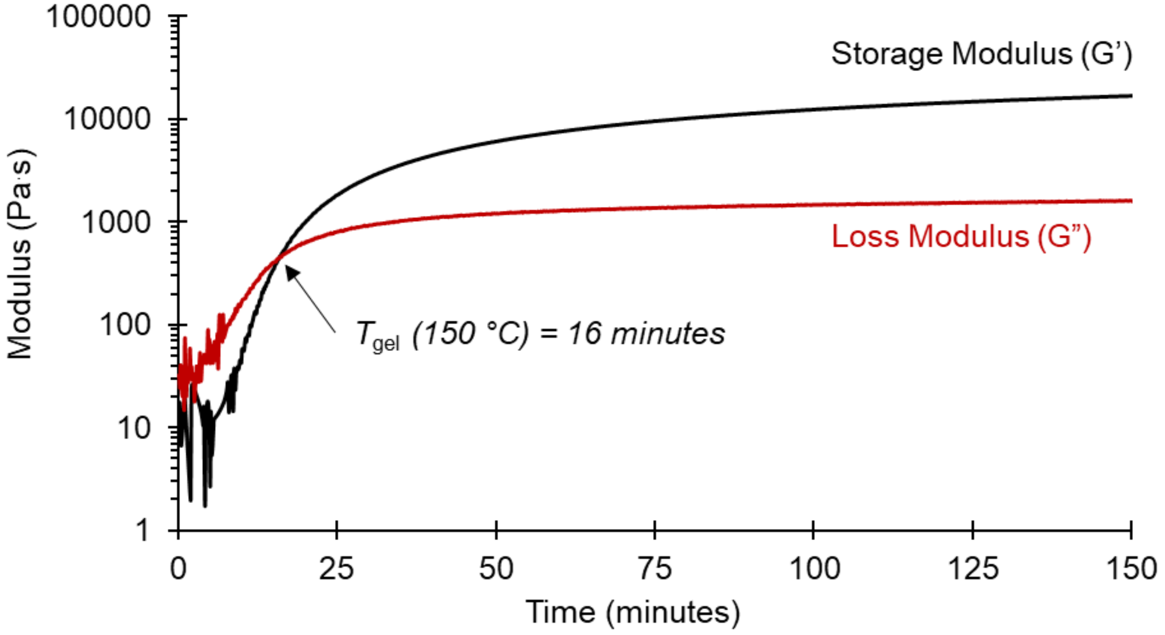

Fig. S58. SAOS of **1**/TREN. Experimental conditions: 1.0% strain, 10 rads/s, 150°C.

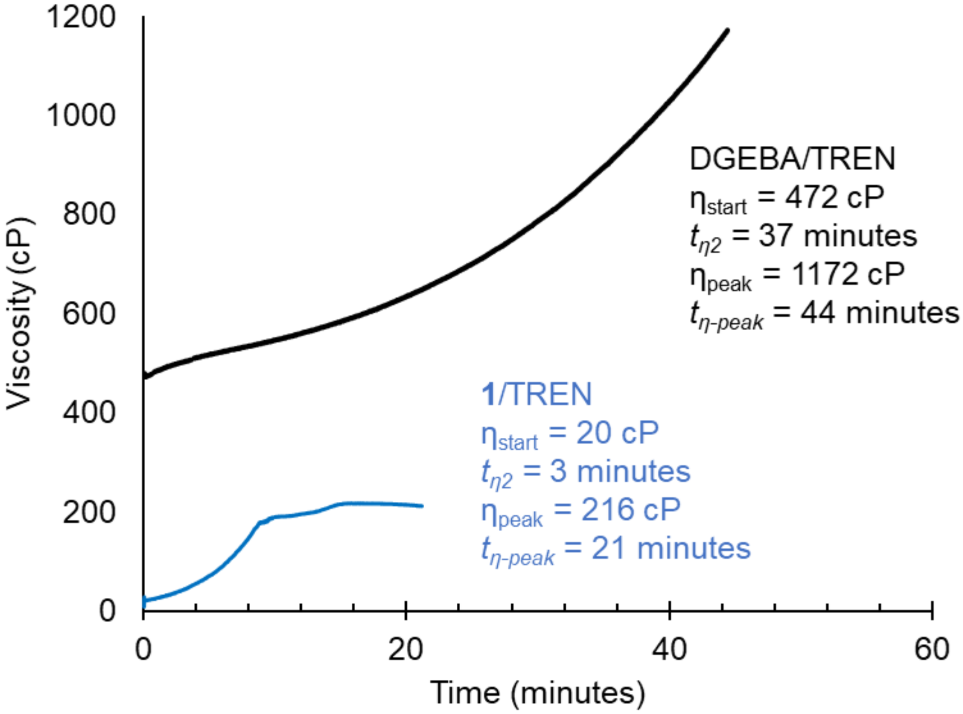

Fig. S59. Viscosity vs time of resins. Data acquired using an IKA Rotavisc Lo-Vi viscometer equipped with a 2.1 mL low volume water cooled reservoir and corresponding spindle. Data point at $t_{\eta 2}$ represents the time point at which the viscosity has doubled since t_0. The data point at η_{peak} represents the viscosity at which the resin cured to a point that the spindle rotates freely.

Environmental Exposure

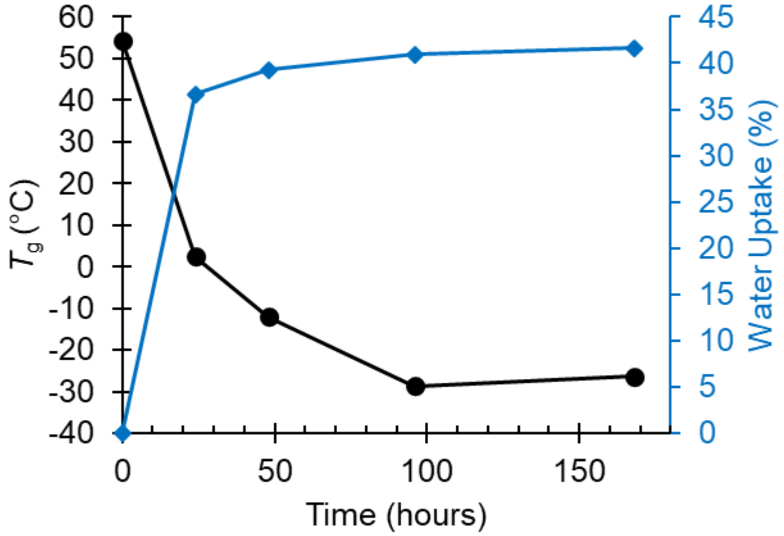

Fig. S60. Elevated temperature and humidity exposure. Samples stored in an environmental chamber held at a constant 40 °C and 93% relative humidity in accordance with standard IEC-60068-2-78. T_g (black) and water uptake (blue) acquired immediately following removal from chamber.

Sol-Gel Analysis of 1/TREN Matrix

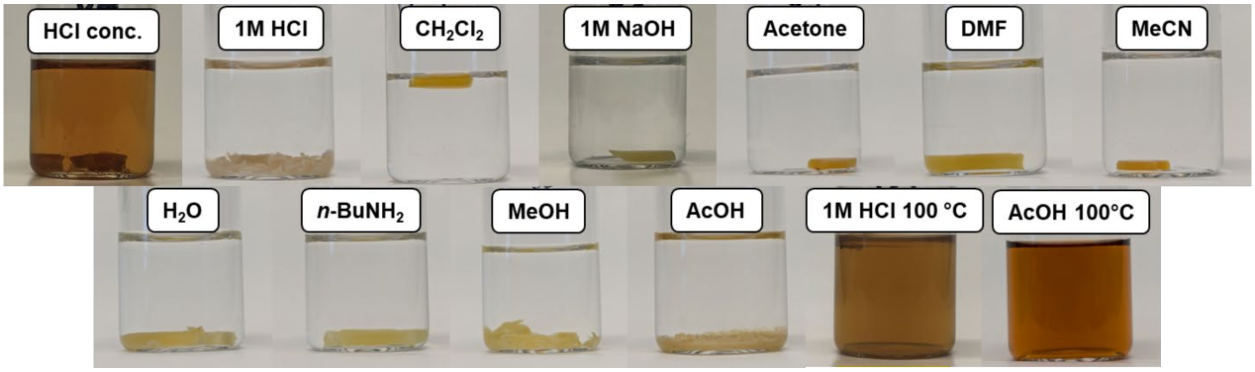

Fig. S61. Sol-gel specimen after 24 hours undisturbed in respective solvent conditions. All sol-gel experiments conducted at 25°C unless otherwise noted.

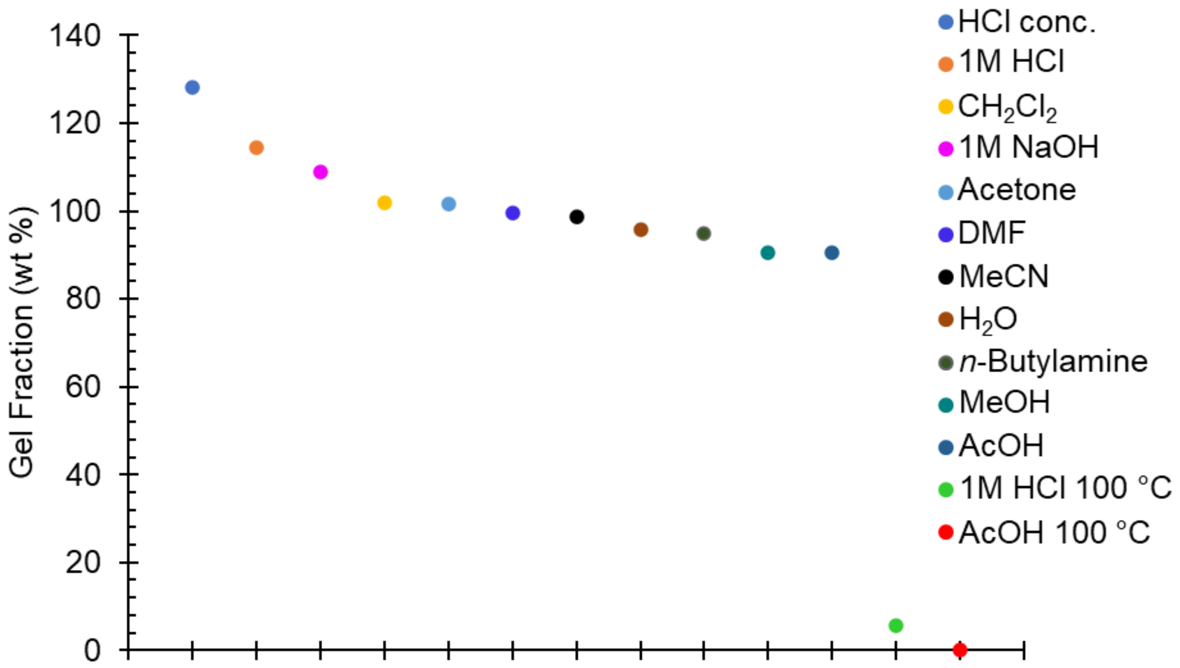

Fig. S62. Sol-gel study gel fraction results. All samples dried in vacuum oven at 60 ºC for 24 hours. Values above 100% are expected to be a result of the formation of ammonium salts with the poly(amidoamine) networks. Experiments performed in triplicate.

Table S4. Gel Fraction results.

Solvent	Mean (%)	St. Dev.
HCl conc.	128.2	4.2
1M HCl	114.5	0.8
CH_2Cl_2	109.1	2.5
1M NaOH	102.0	1.4
Acetone	101.7	3.4
DMF	99.7	2.2
MeCN	98.8	1.6
H_2O	95.7	1.4
n-Butylamine	95.0	1.1
MeOH	90.6	1.5
AcOH	90.5	5.4
1M HCl 100 °C	5.5	0.4
AcOH 100 °C	0.0	-

Sol-gel study gel fraction results. All samples dried in vacuum oven at 60 ºC for 24 hours. Values above 100% are expected to be a result of the formation of ammonium salts with the poly(amidoamine) networks. Experiments performed in triplicate.

Thermoset Digestion and Recovery

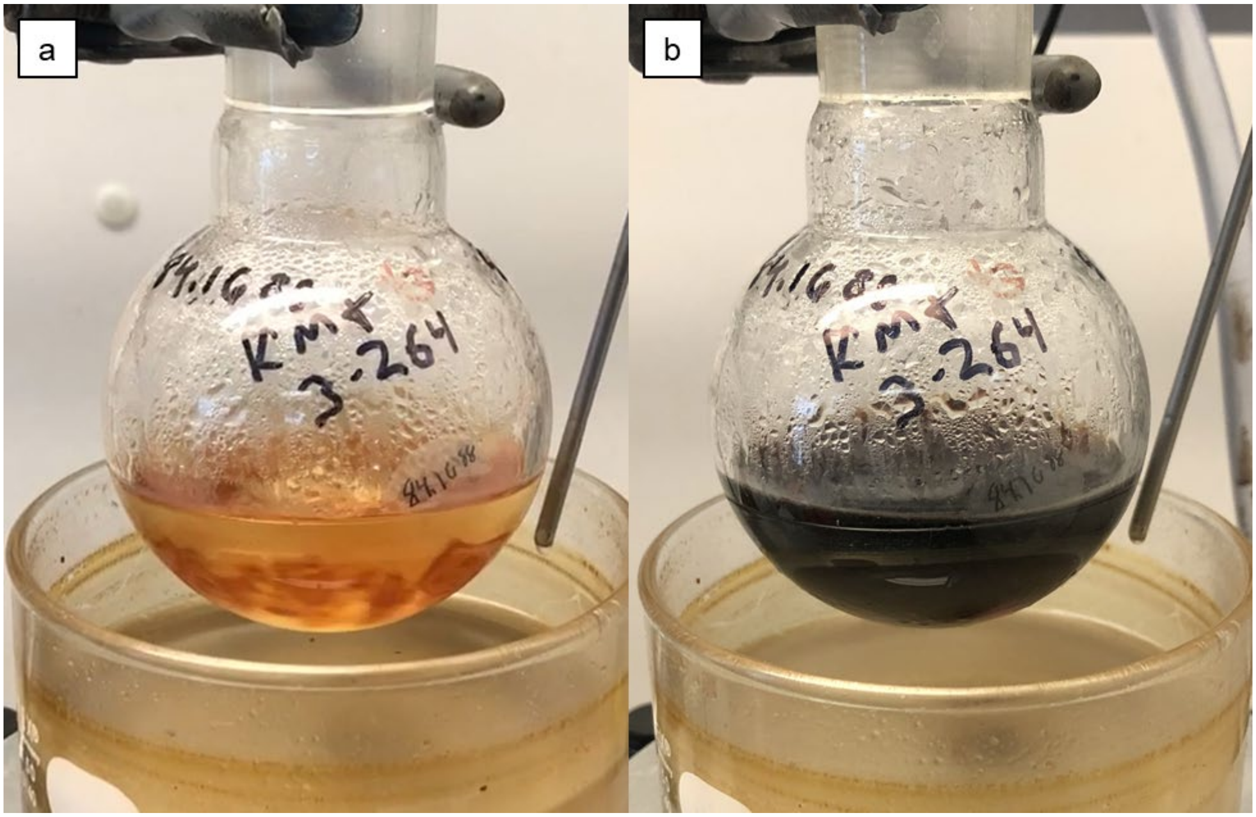

Fig. S63. Digestion of **1**/TREN matrix in AcOH at 100°C. Images taken a) at t = 0 hours where digestion mixture was heterogeneous, and b) t = 4 hours where digestion mixture was homogenous.

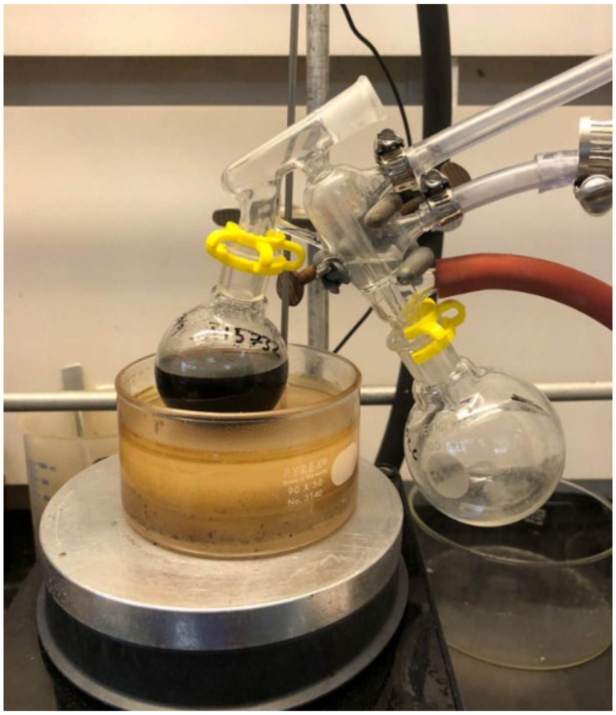

Fig. S64. Vacuum distillation set-up to isolate **1** from digestion mixture.

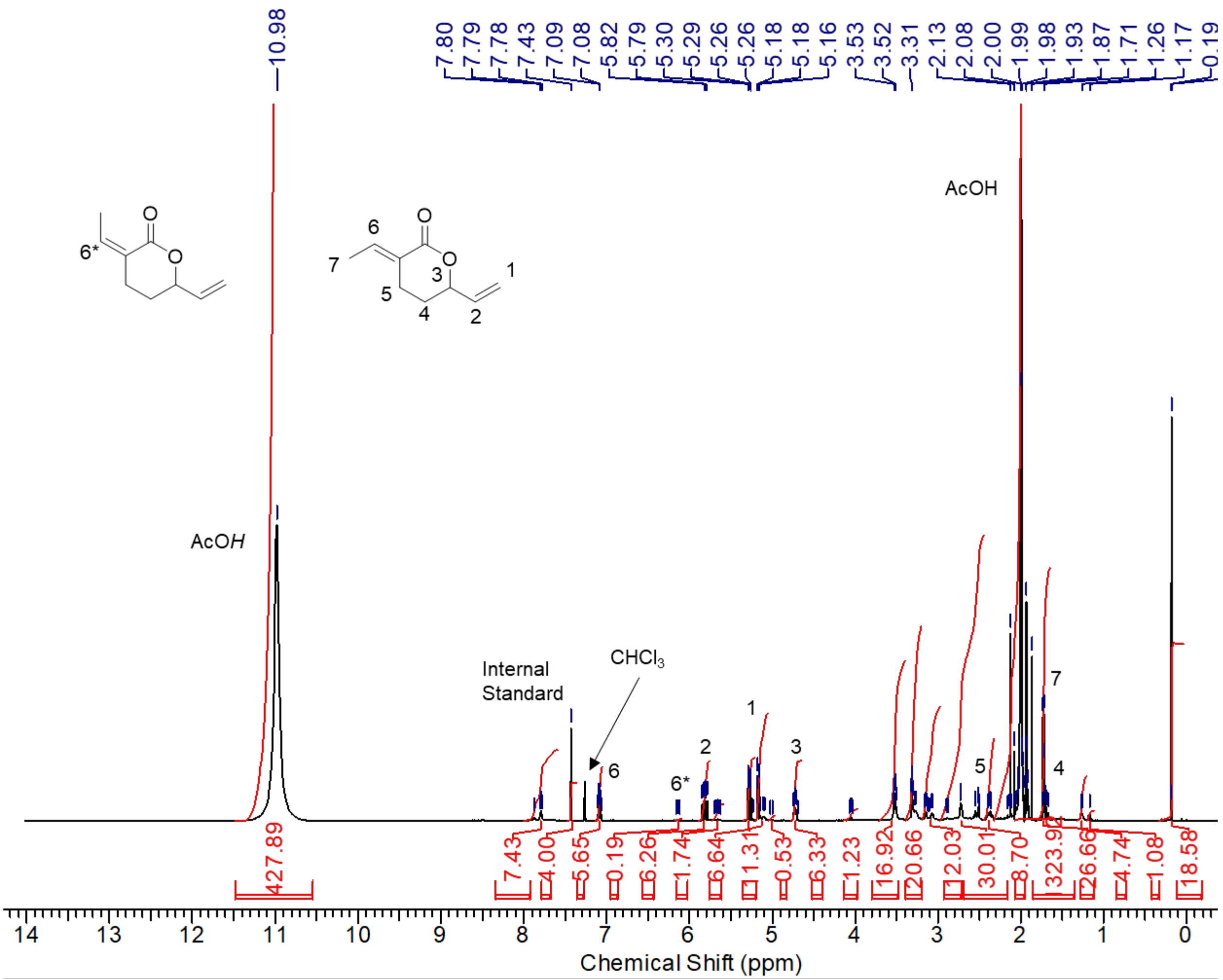

Fig. S65. ^{1}H NMR (500 MHz, $CDCl_3$) of crude digestion mixture. Internal Standard used is 1,4-Bis(trimethylsilyl)benzene. Both *E* and *Z* isomers are observed.(*47*)

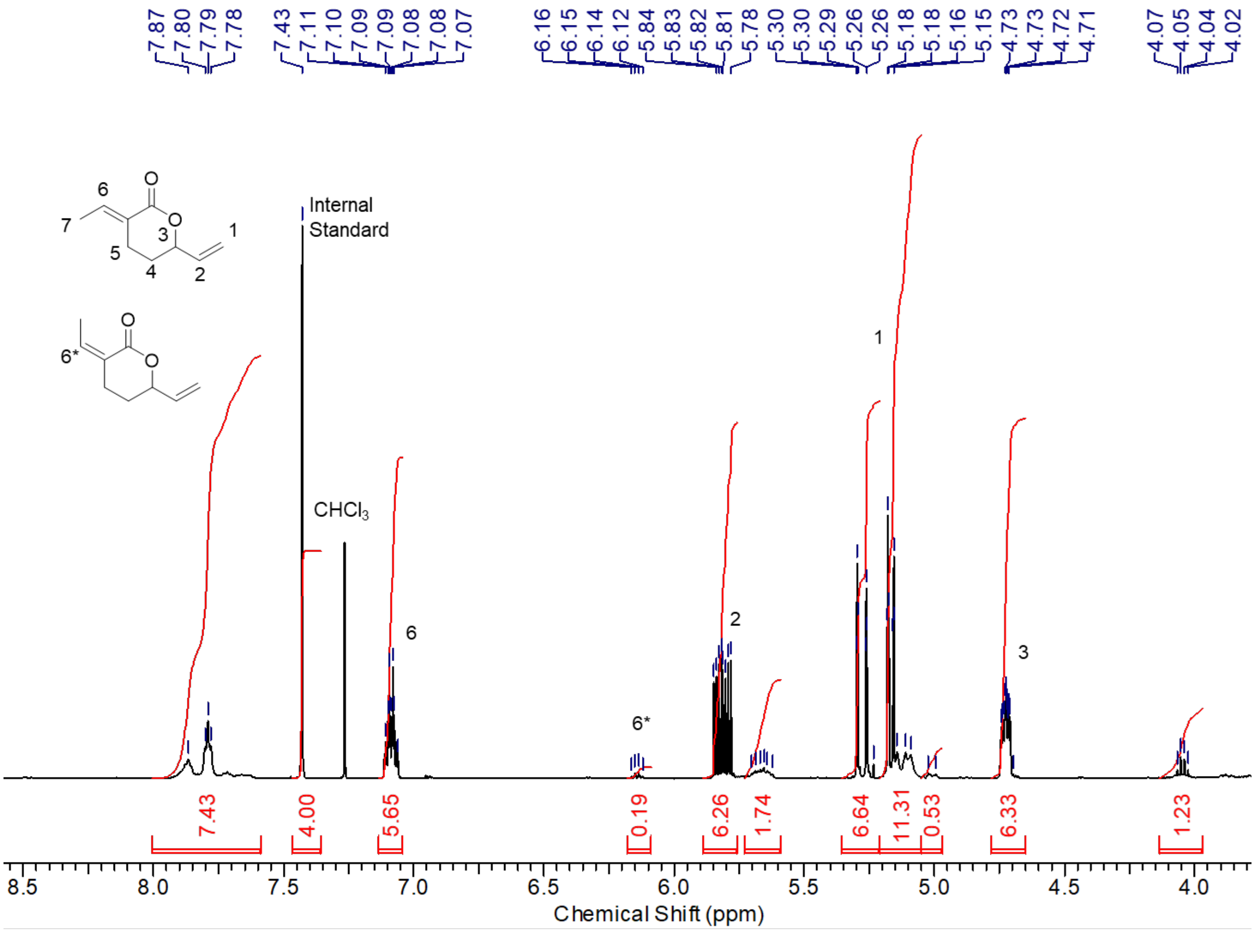

Fig. S66. Enlarged ^{1}H NMR (500 MHz, $CDCl_3$) of crude digestion mixture. Internal standard used is 1,4-bis(trimethylsilyl)benzene. Both *E* and *Z* isomers are observed.(*47*)

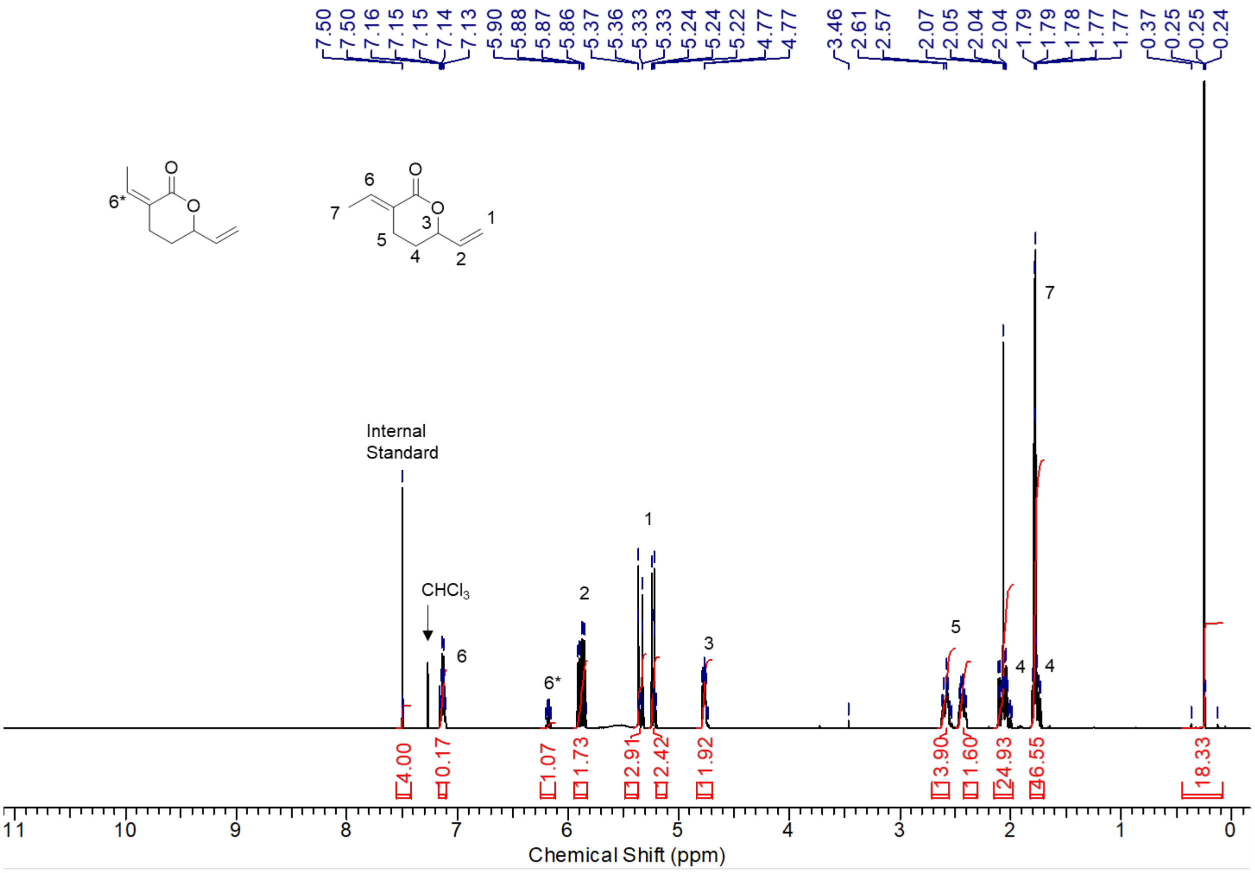

Fig. S67. ^{1}H NMR (500 MHz, $CDCl_3$) of distillate acquired from distillation of digestion mixture. Internal standard used is 1,4-bis(trimethylsilyl)benzene. Both *E* and *Z* isomers are observed.(*47*)

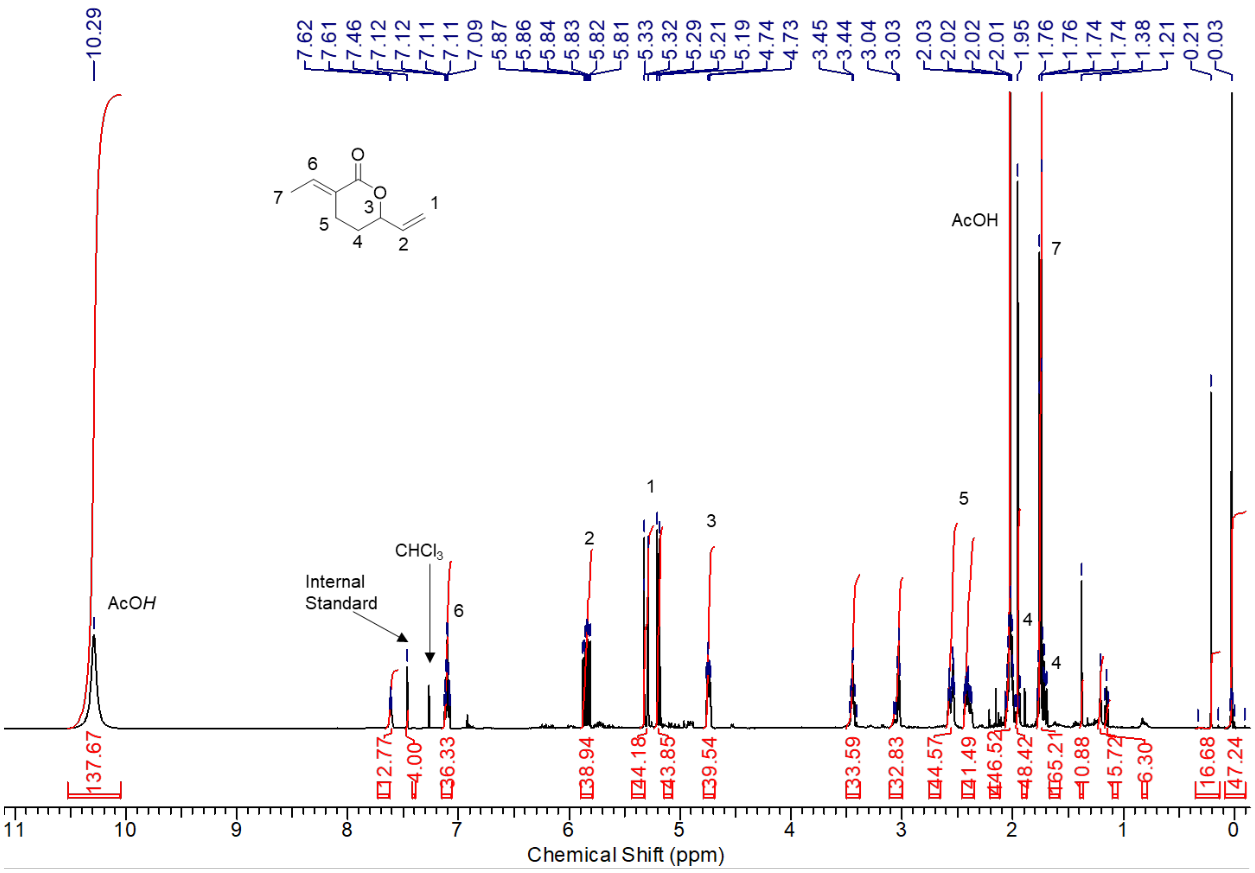

Fig. S68. ^{1}H NMR (500 MHz, $CDCl_3$) of extract acquired by concentration and subsequent precipitation of digestion mixture in Et_2O. The Et_2O layer is separated and concentrated, yielding **1** and various amidoamine species. Internal standard used is 1,4-bis(trimethylsilyl)benzene.

DSC of Matrices

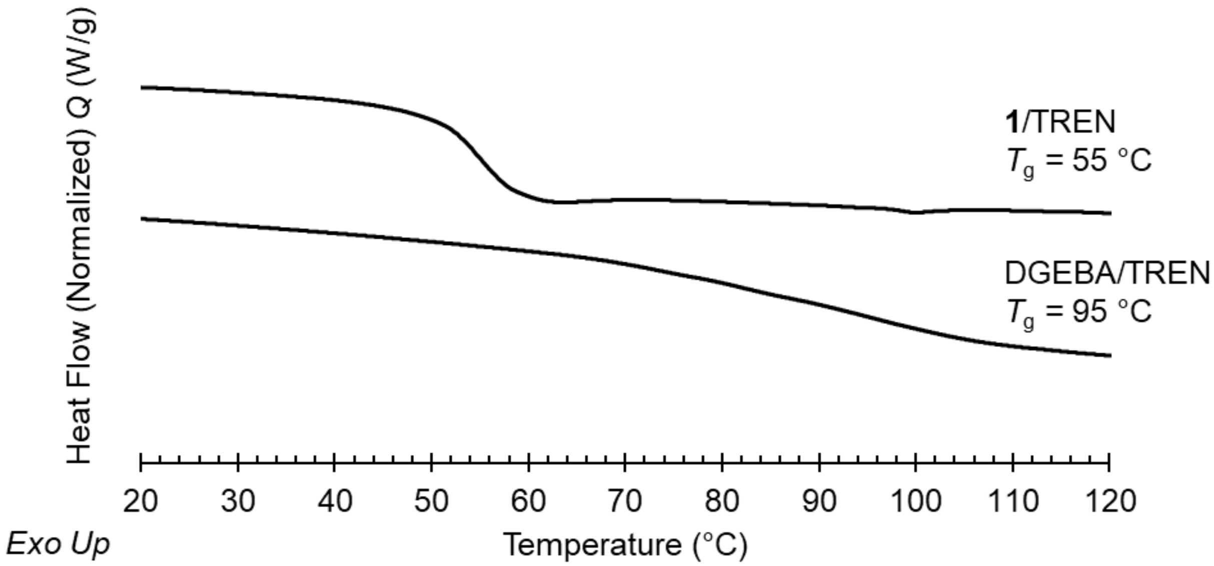

Fig. S69. DSC traces of matrix materials mechanically tested, performed at 10°C/minute.

TGA of Matrices and Composites

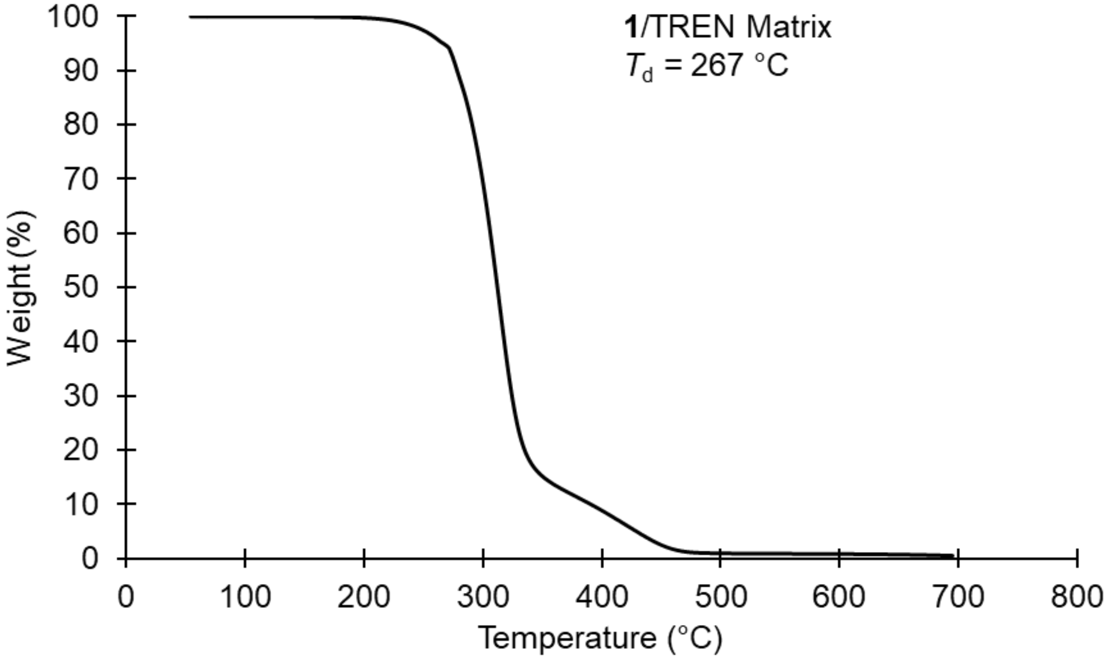

Fig. S70. TGA of **1**/TREN matrix at 10°C/minute.

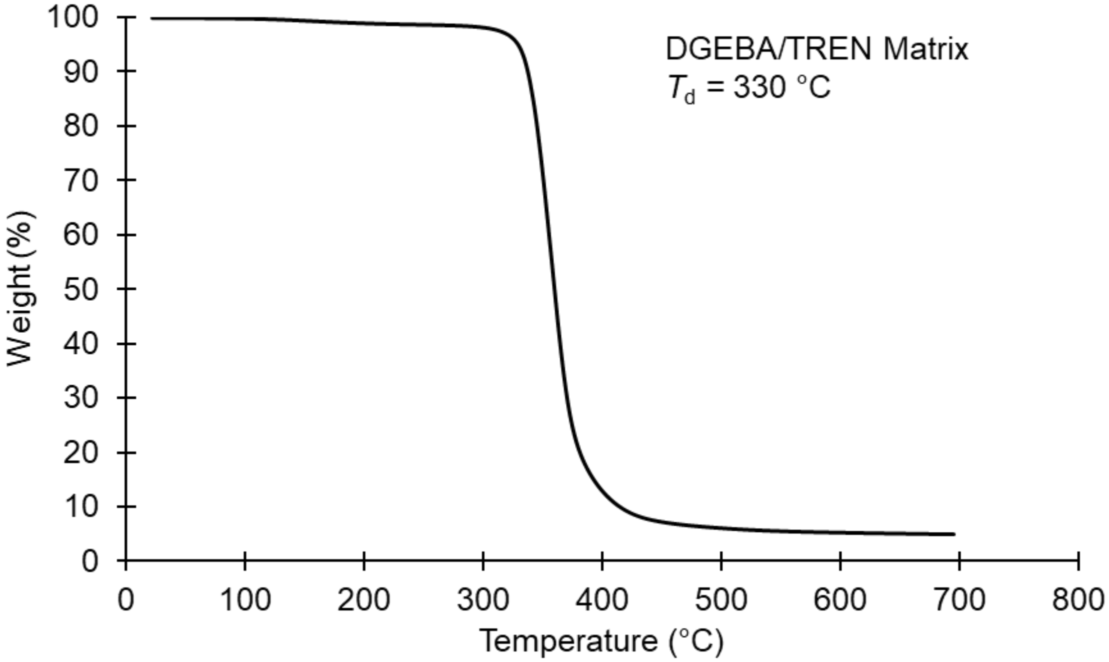

Fig. S71. TGA of DGEBA/TREN matrix at 10°C/minute.

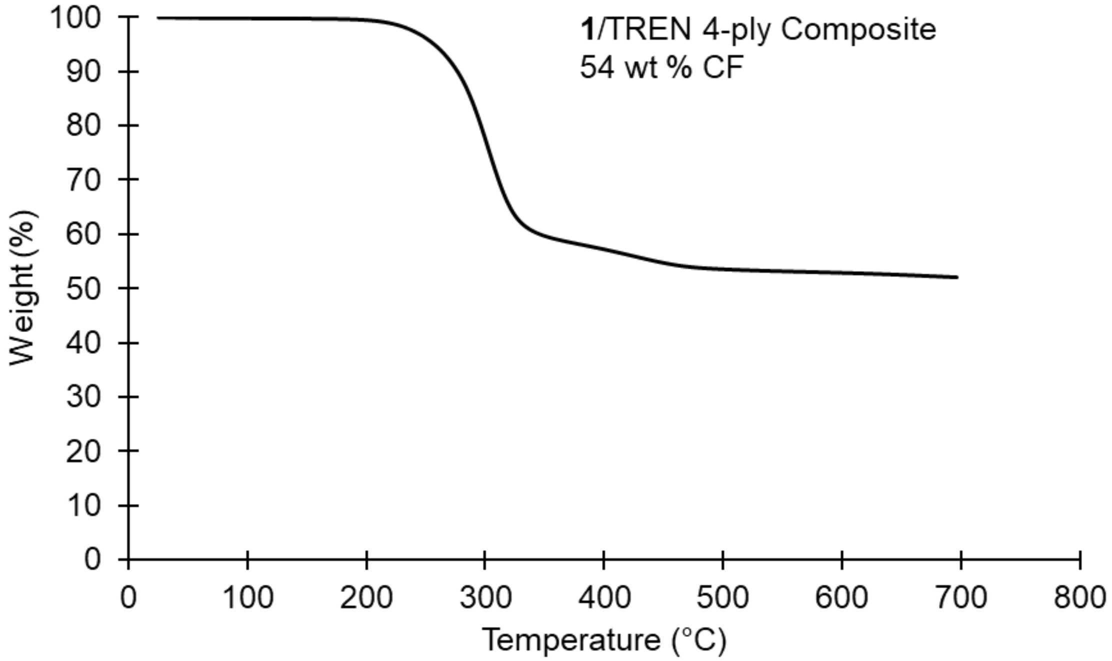

Fig. S72. TGA of **1**/TREN 4-ply composite at 10°C/minute. Carbon fiber weight content measured at 500°C.(*28*)

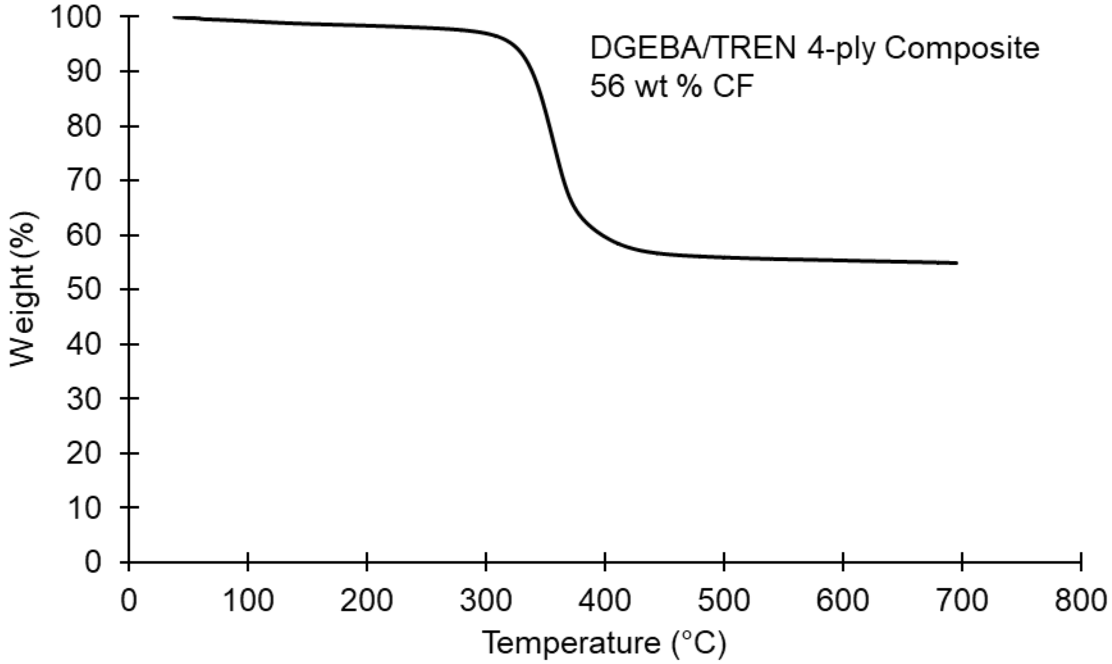

Fig. S73. TGA of DGEBA/TREN 4-ply composite at 10°C/minute. Carbon fiber weight content measured at 500°C.(*28*)

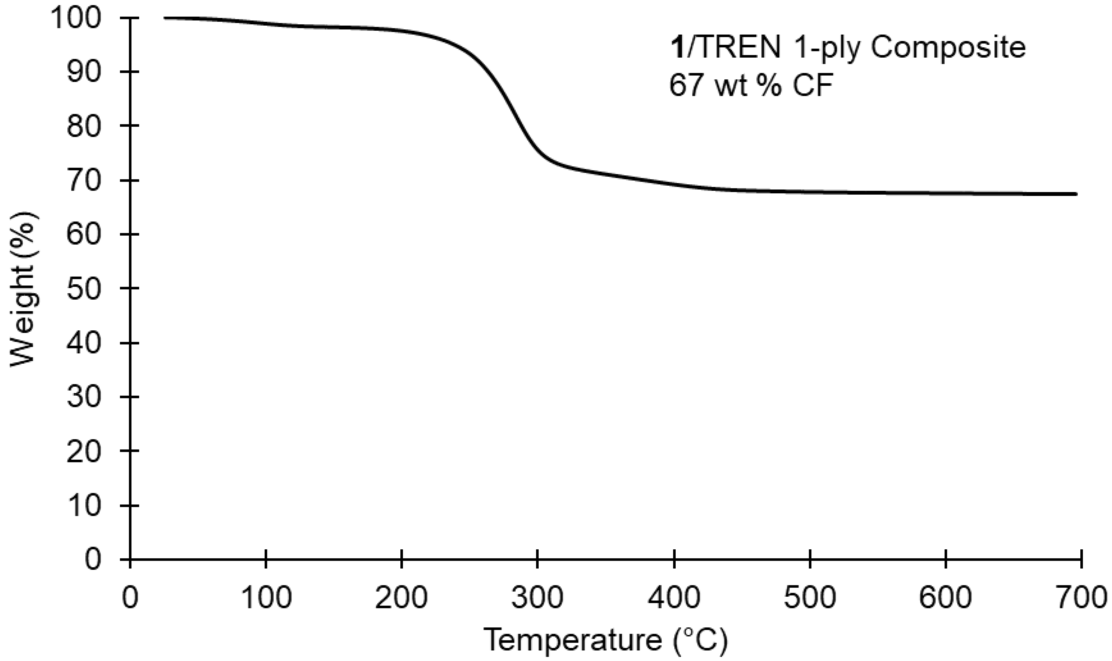

Fig. S74. TGA of **1**/TREN 1-ply composite at 10°C/minute. Carbon fiber weight content measured at 500°C.(*28*)

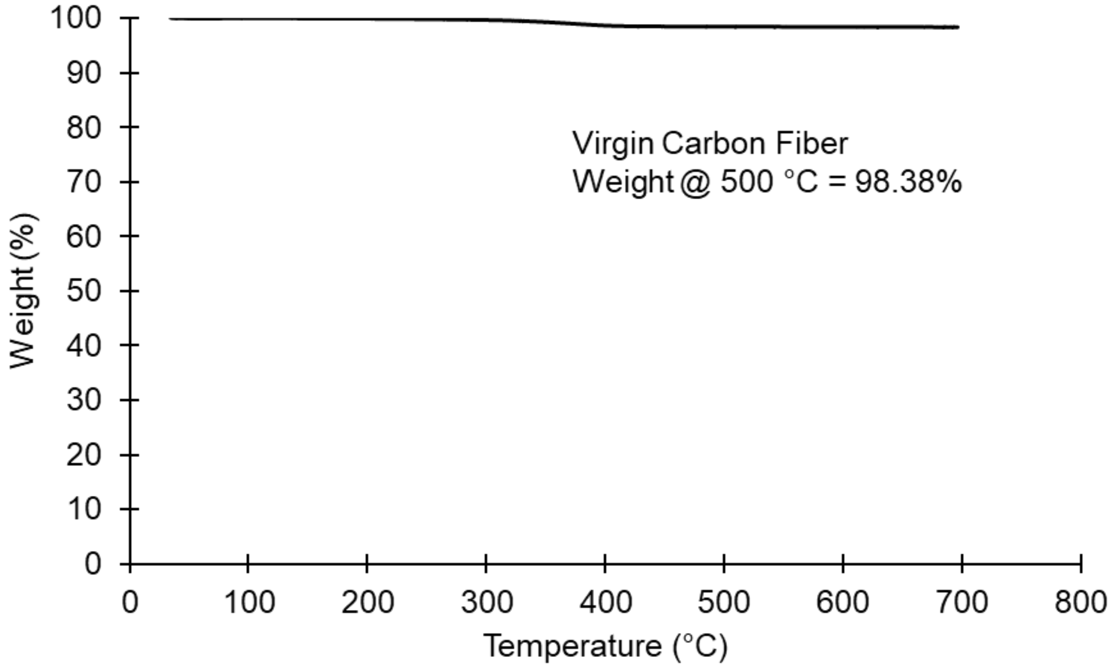

Fig. S75. TGA of virgin plain weave carbon fiber at 10°C/minute. Carbon fiber weight content measured at 500°C.(*28*)

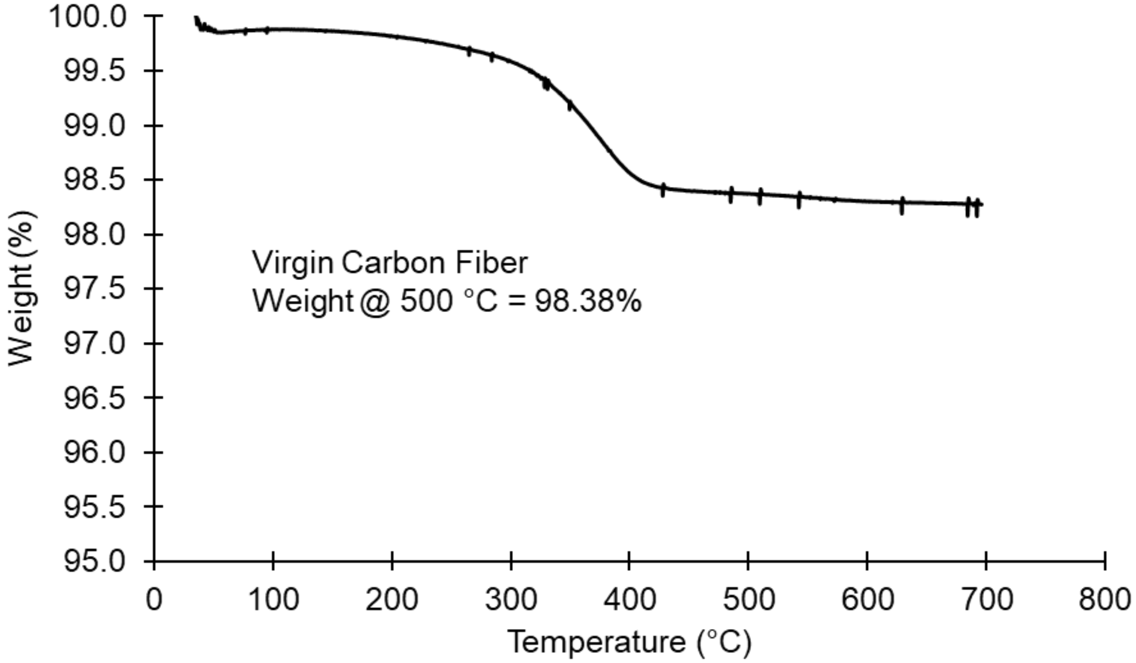

Fig. S76. Magnified TGA of virgin plain weave carbon fiber at 10°C/minute. Carbon fiber weight content measured at 500°C.(*28*)

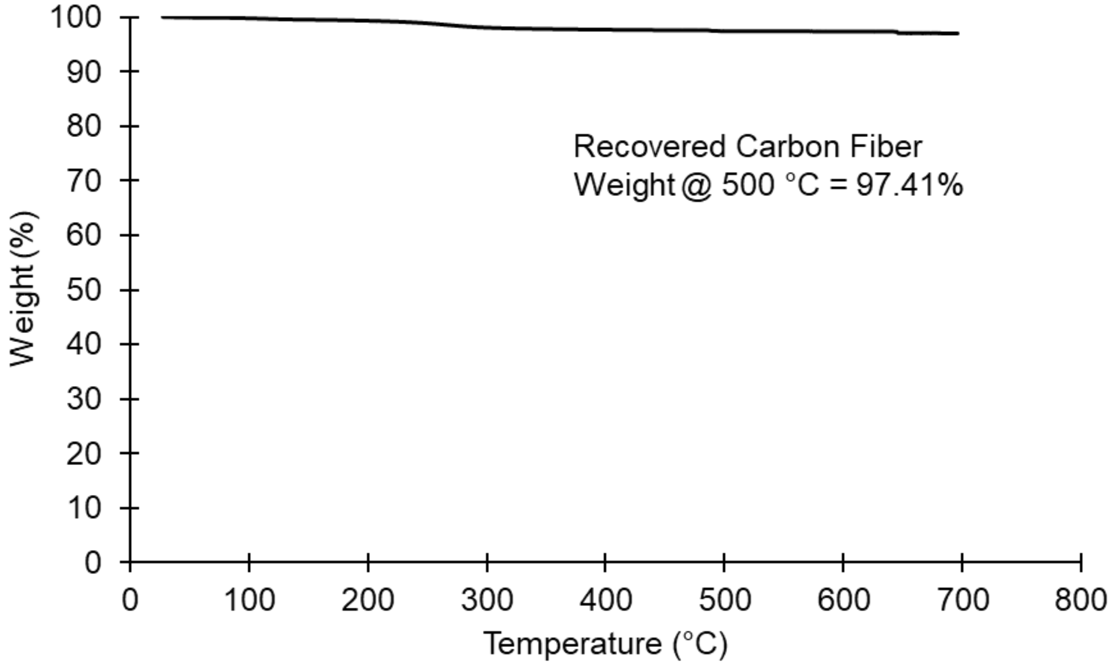

Fig. S77. TGA of recovered plain weave carbon fiber at 10°C/minute. Carbon fiber weight content measured at 500°C.(*28*)

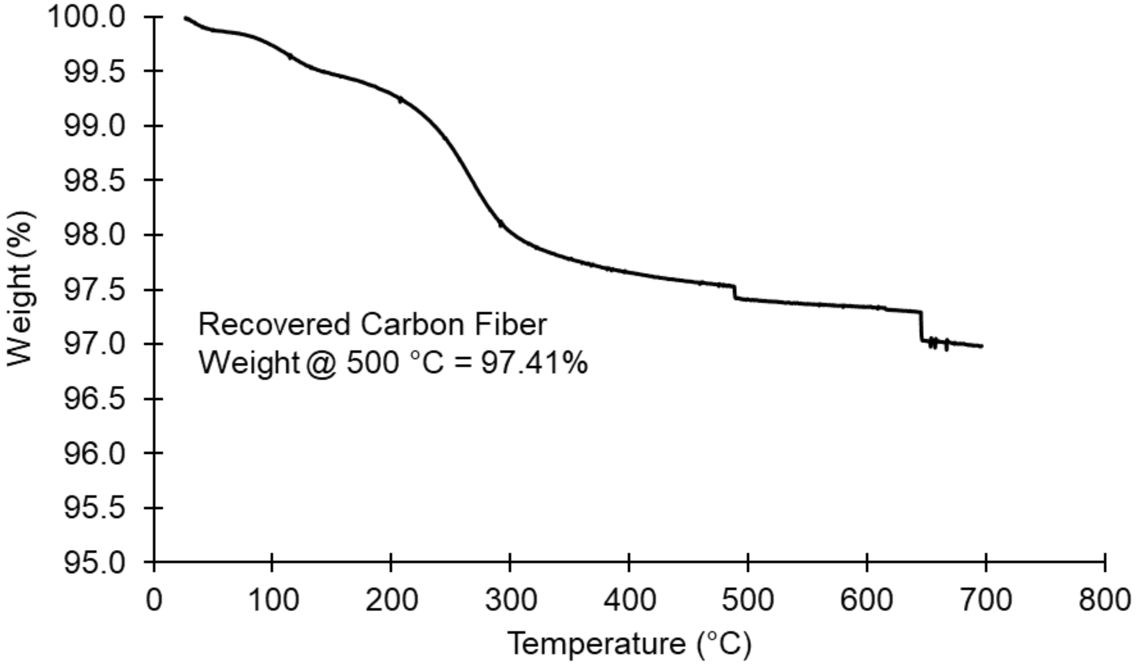

Fig. S78. Magnified TGA of recovered plain weave carbon fiber at 10°C/minute. Carbon fiber weight content measured at 500°C.(*28*)

Mechanical Analyses

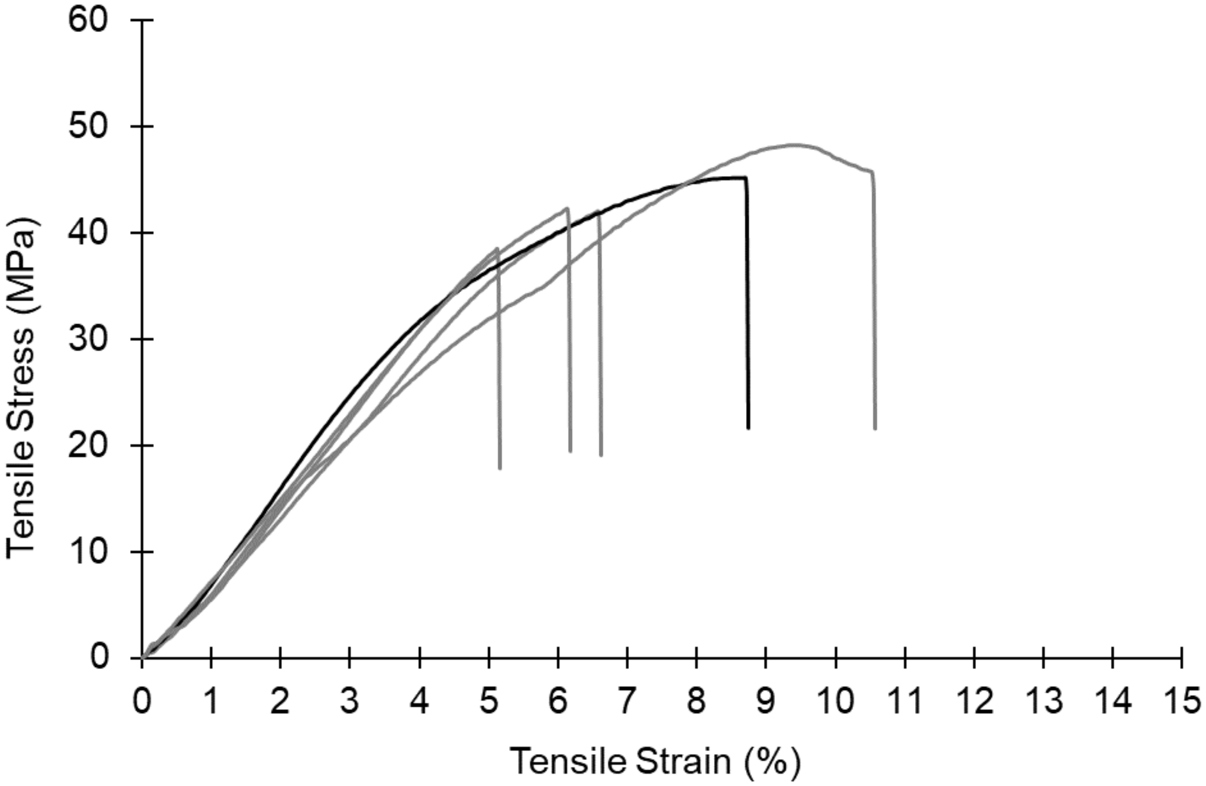

Fig. S79. Uniaxial tensile testing of **1**/TREN matrix. Performed in accordance with ASTM D638.(*54*)

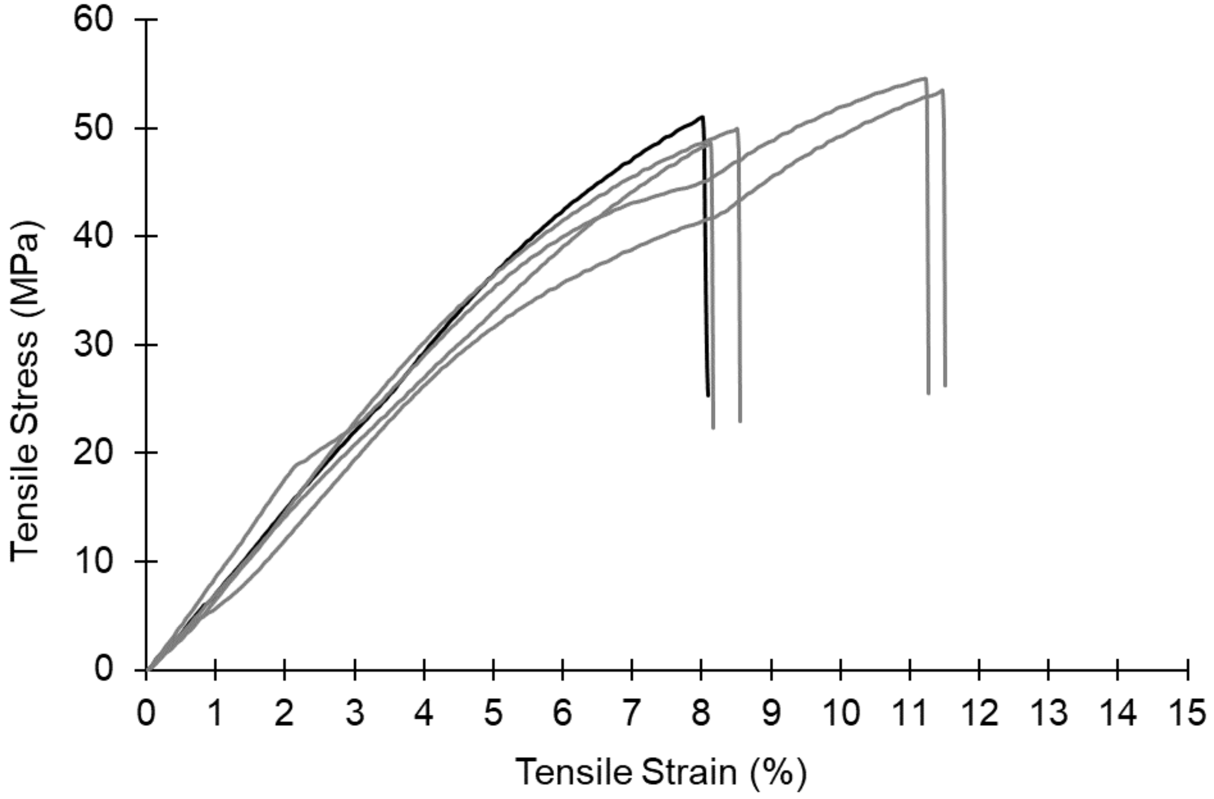

Fig. S80. Uniaxial tensile testing of DGEBA/TREN matrix. Performed in accordance with ASTM D638.(*54*)

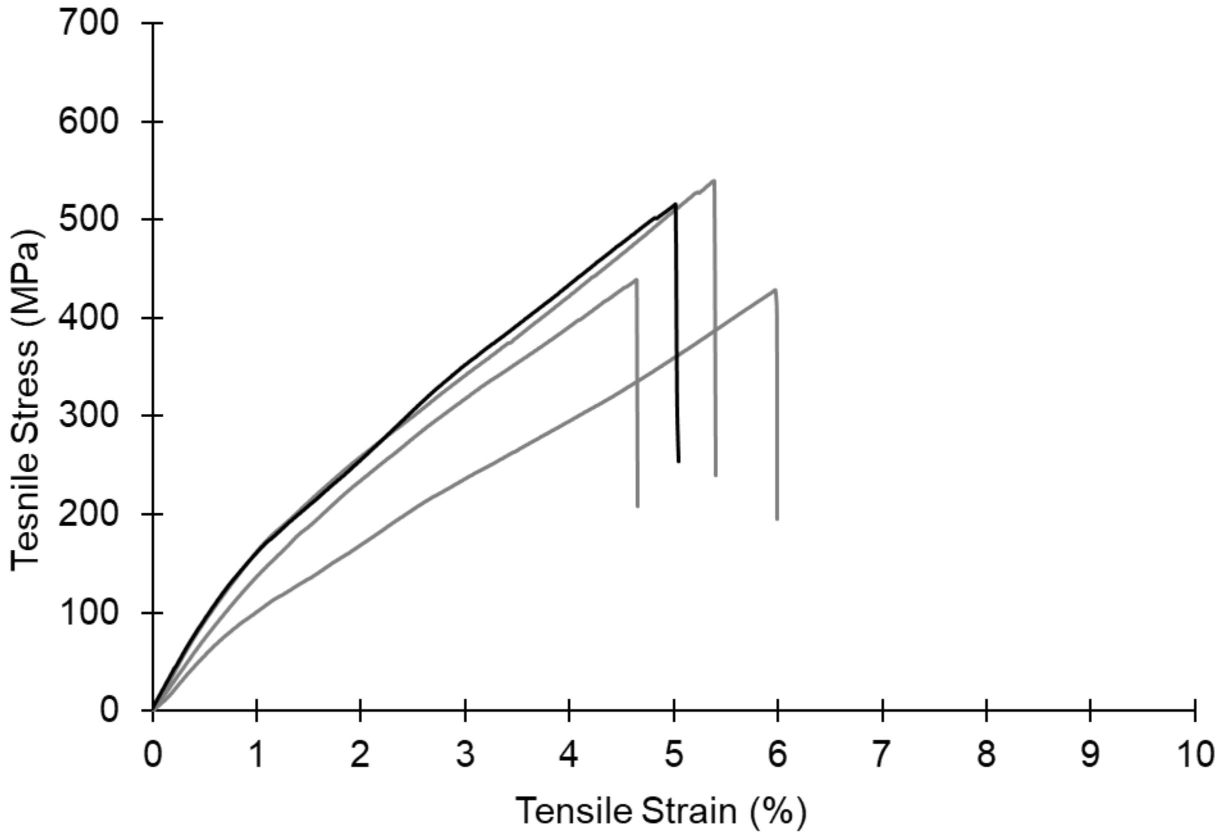

Fig. S81. Uniaxial tensile testing of **1**/TREN 4-ply composite. Performed in accordance with ASTM D3039.(*55*)

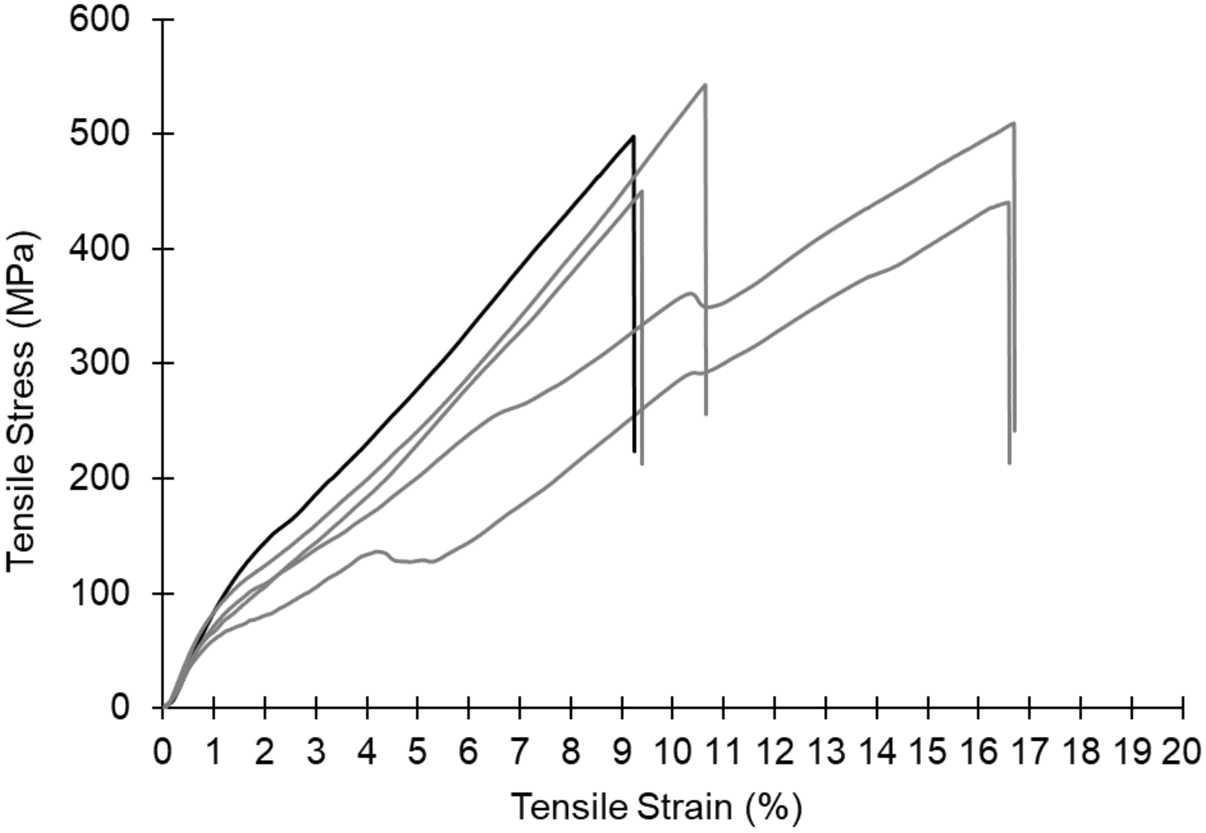

Fig. S82. Uniaxial tensile testing of DGEBA/TREN 4-ply composite. Performed in accordance with ASTM D3039.(*55*)

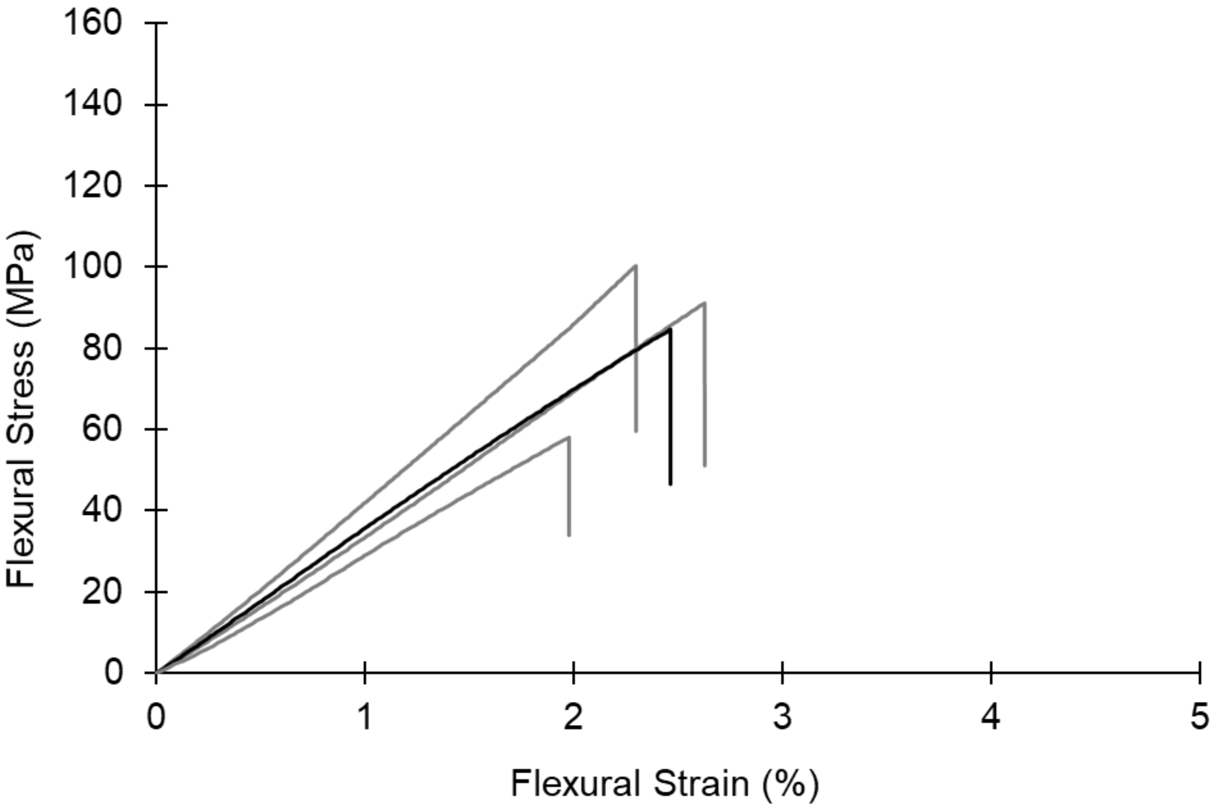

Fig. S83. Flexural testing of **1**/TREN matrix. Performed in accordance with ASTM D790.(*56*)

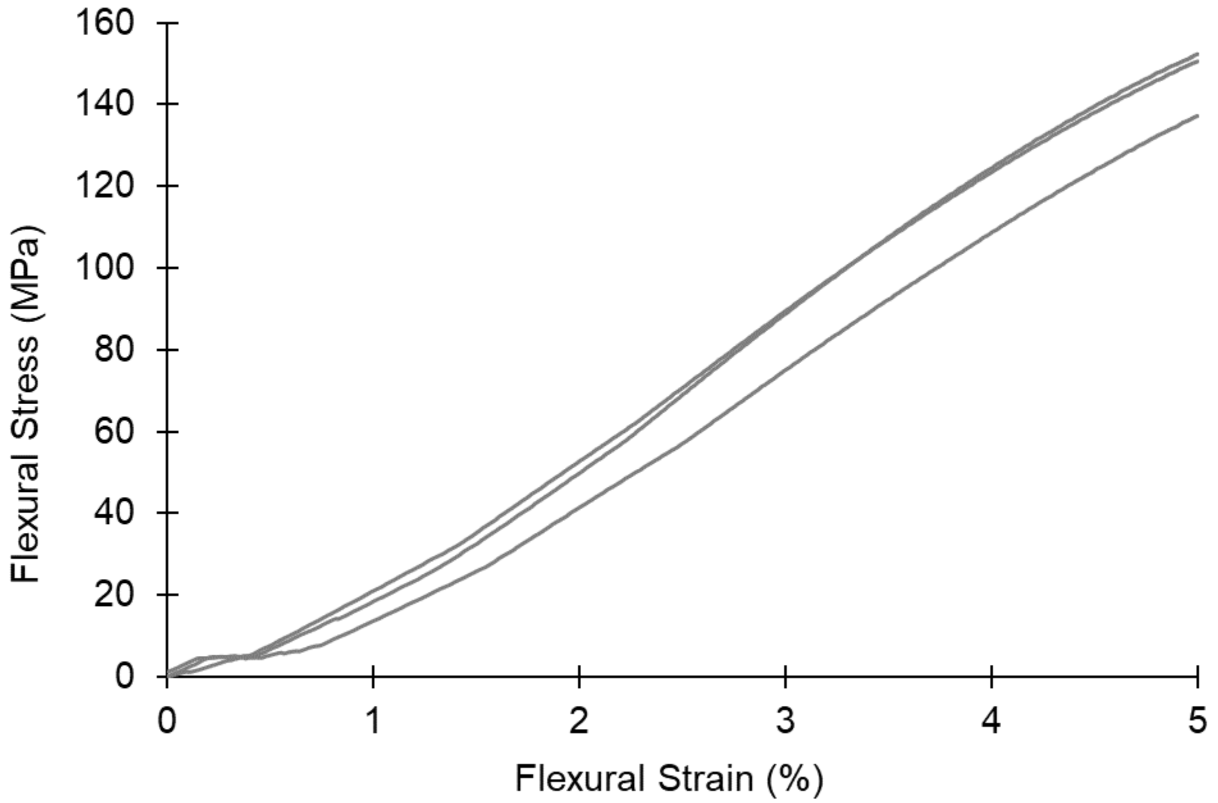

Fig. S84. Flexural testing of DGEBA/TREN matrix. Performed in accordance with ASTM D790.(*56*)

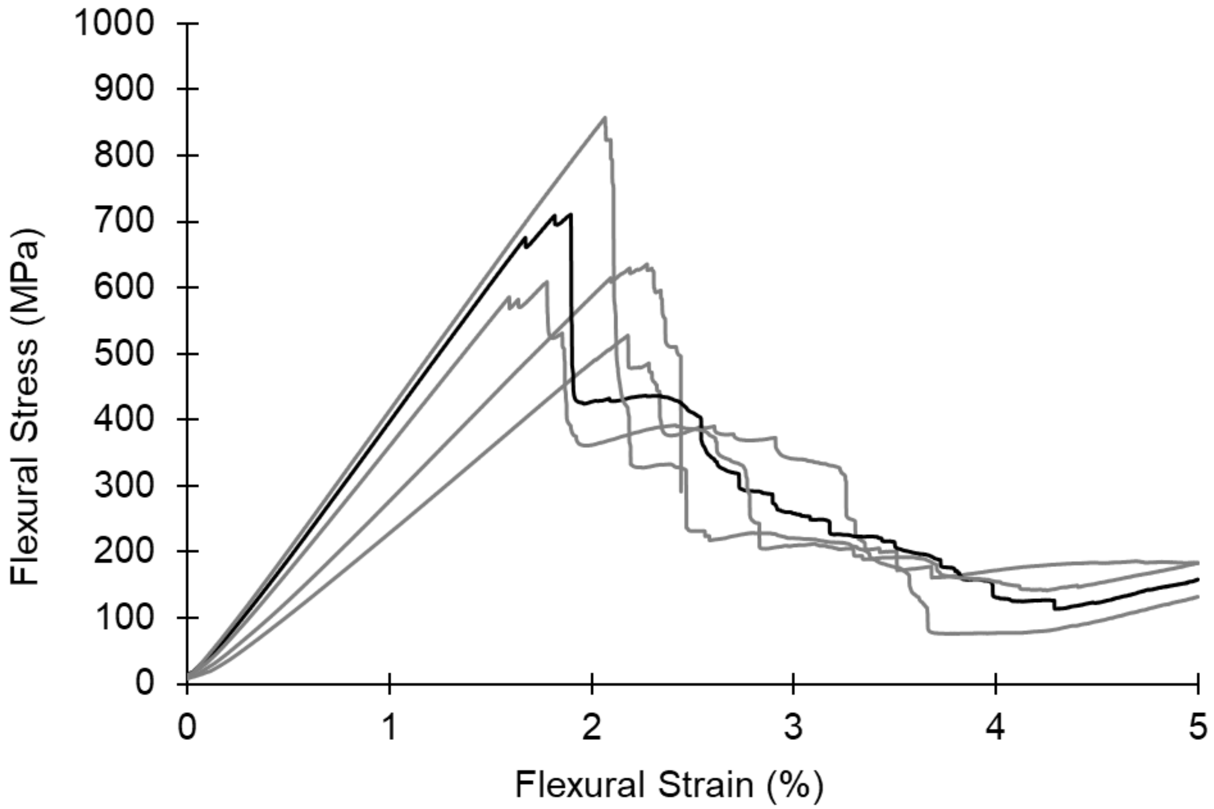

Fig. S85. Flexural testing of **1**/TREN 4-ply composite. Performed in accordance with ASTM D790.(*56*)

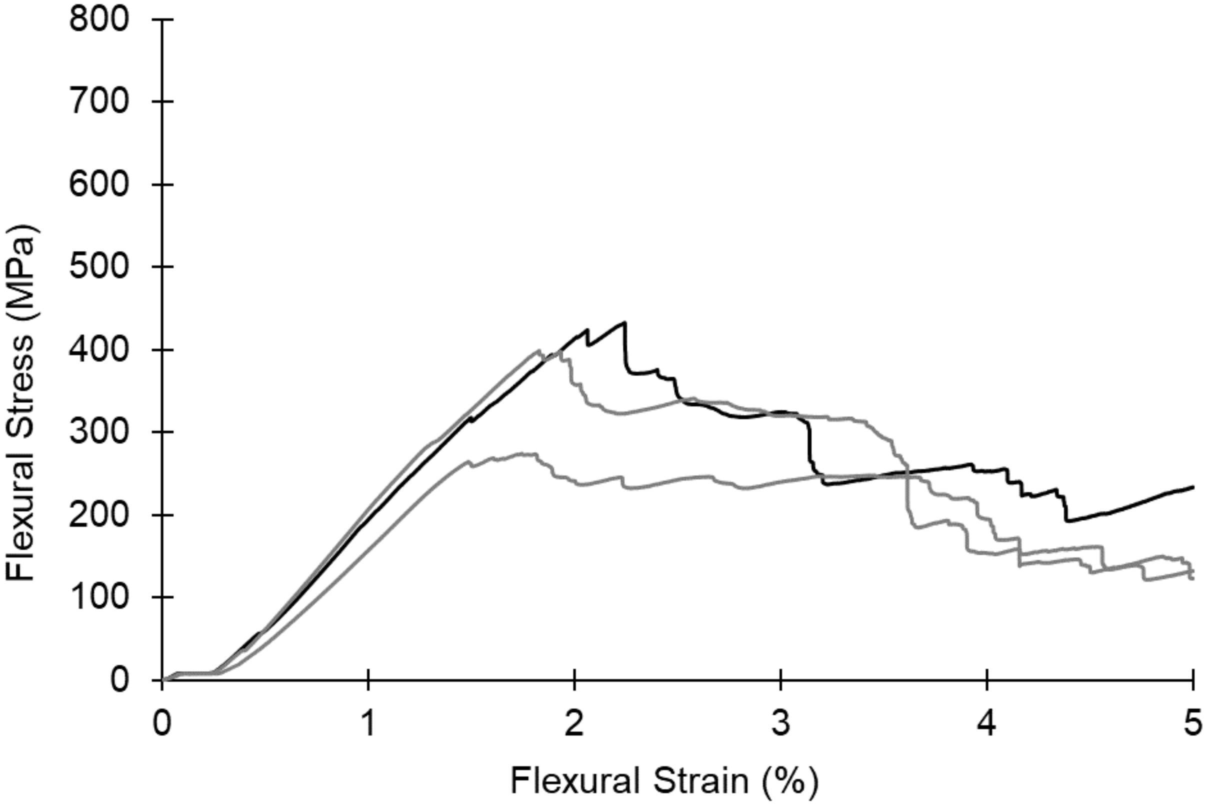

Fig. S86. Flexural testing of DGEBA/TREN 4-ply composite. Performed in accordance with ASTM D790.(*56*)

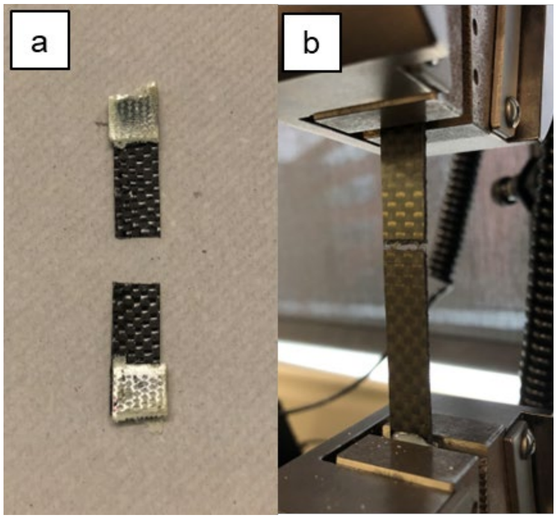

Fig. S87. CFRP tensile specimen showing various methods of failure. a) DGEBA/TREN composite showing lateral, gauge, middle fracture (LGM). b) **1**/TREN composite showing lateral, gauge, middle fracture (LGM). Failure mechanisms assigned according to ASTM D3039 failure codes.(*55*)

SEM Imaging of Composites and Fibers

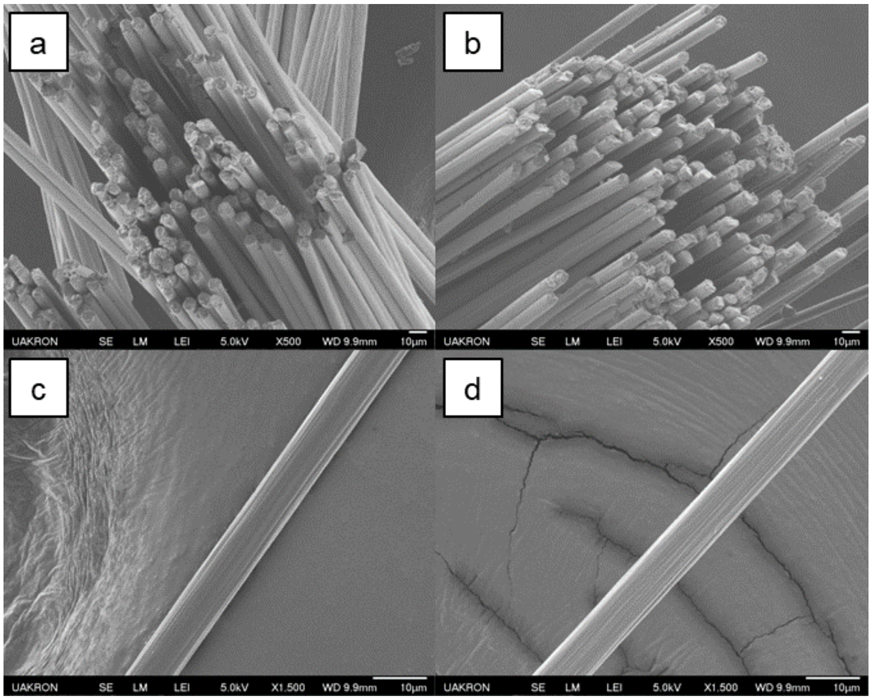

Fig. S88. SEM imaging of virgin plain weave carbon fibers as (a, b) bundles of fibers and (c, d) the side-profile of individual fibers.

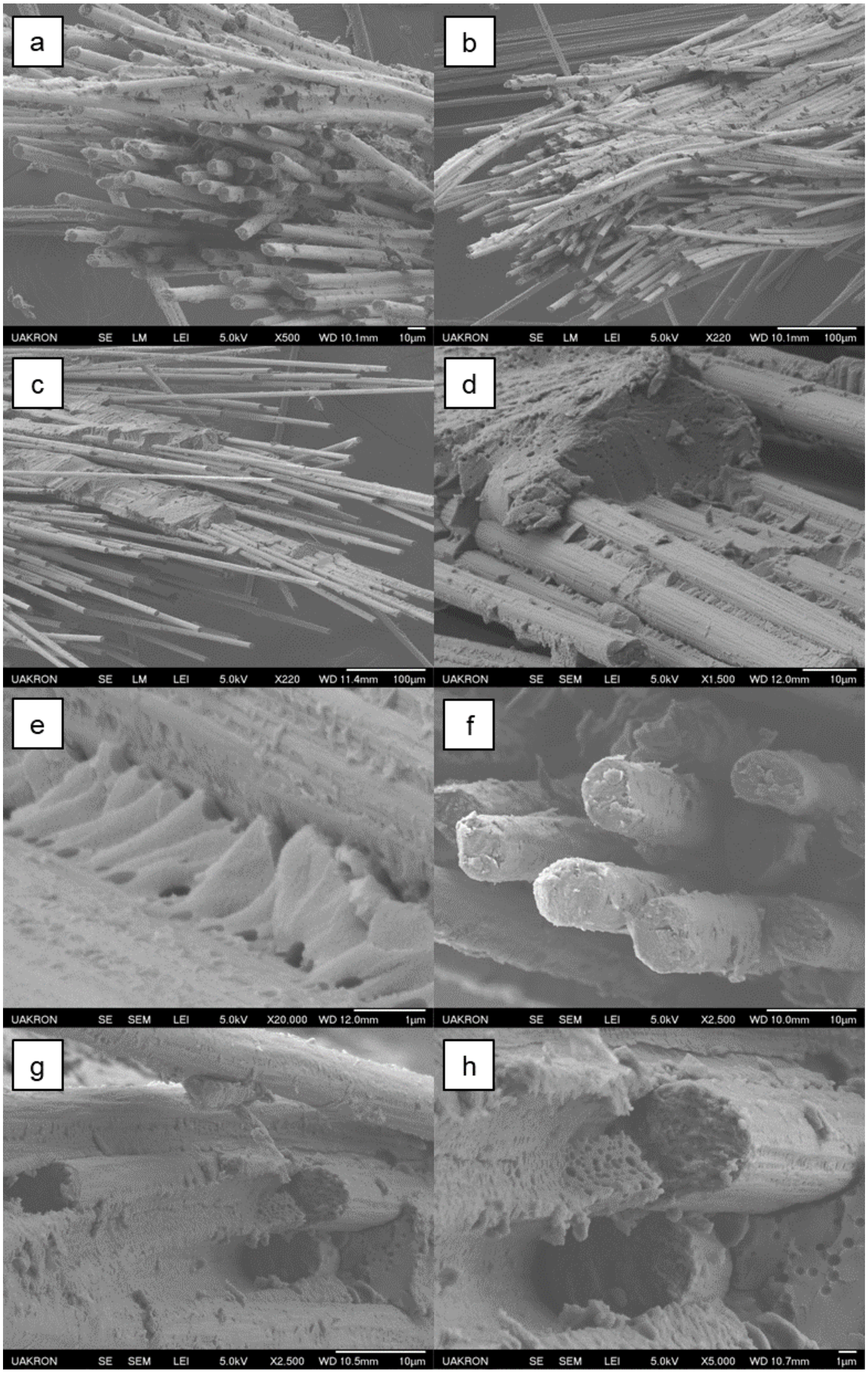

Fig. S89. SEM imaging of **1**/TREN reinforced with plain weave carbon fiber. (a–c) Bundles of fibers from fractured 4-ply composite specimen. (d–h) Close-up showing wetting between fibers and fiber pullout. Inset (a) reused in manuscript Fig 5.

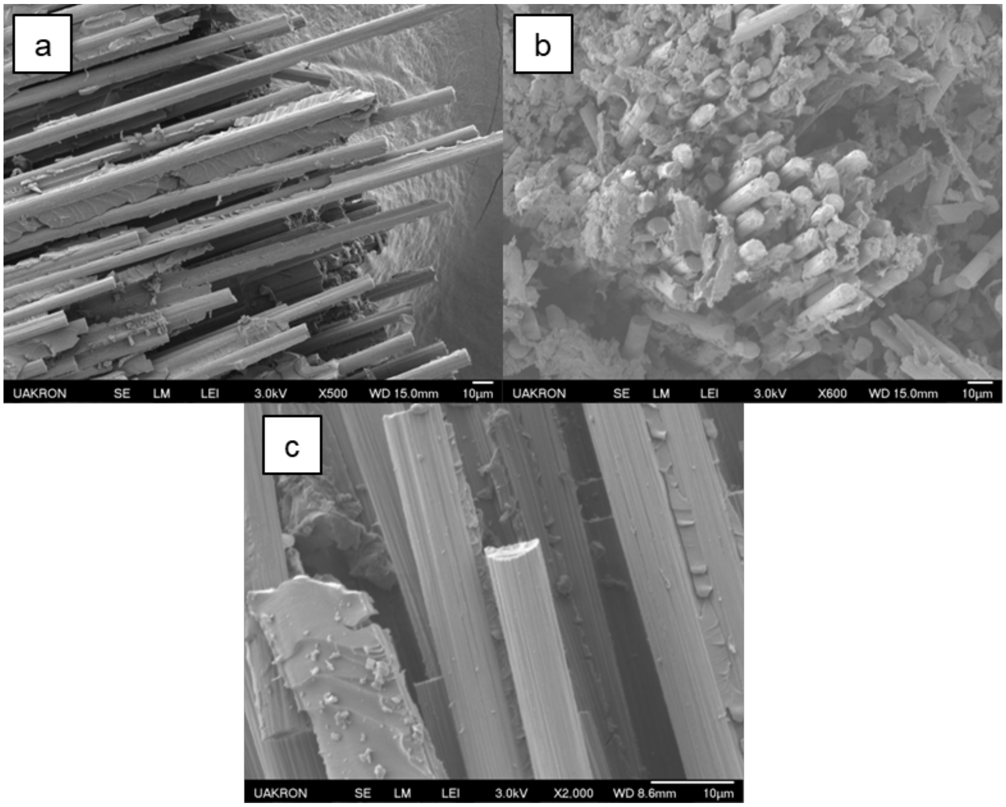

Fig. S90. SEM images of DGEBA/TREN reinforced with plain weave carbon fiber. (a,b) Bundles of fibers from fractured 4-ply composite specimen. (c) Side profile close-up of fibers.

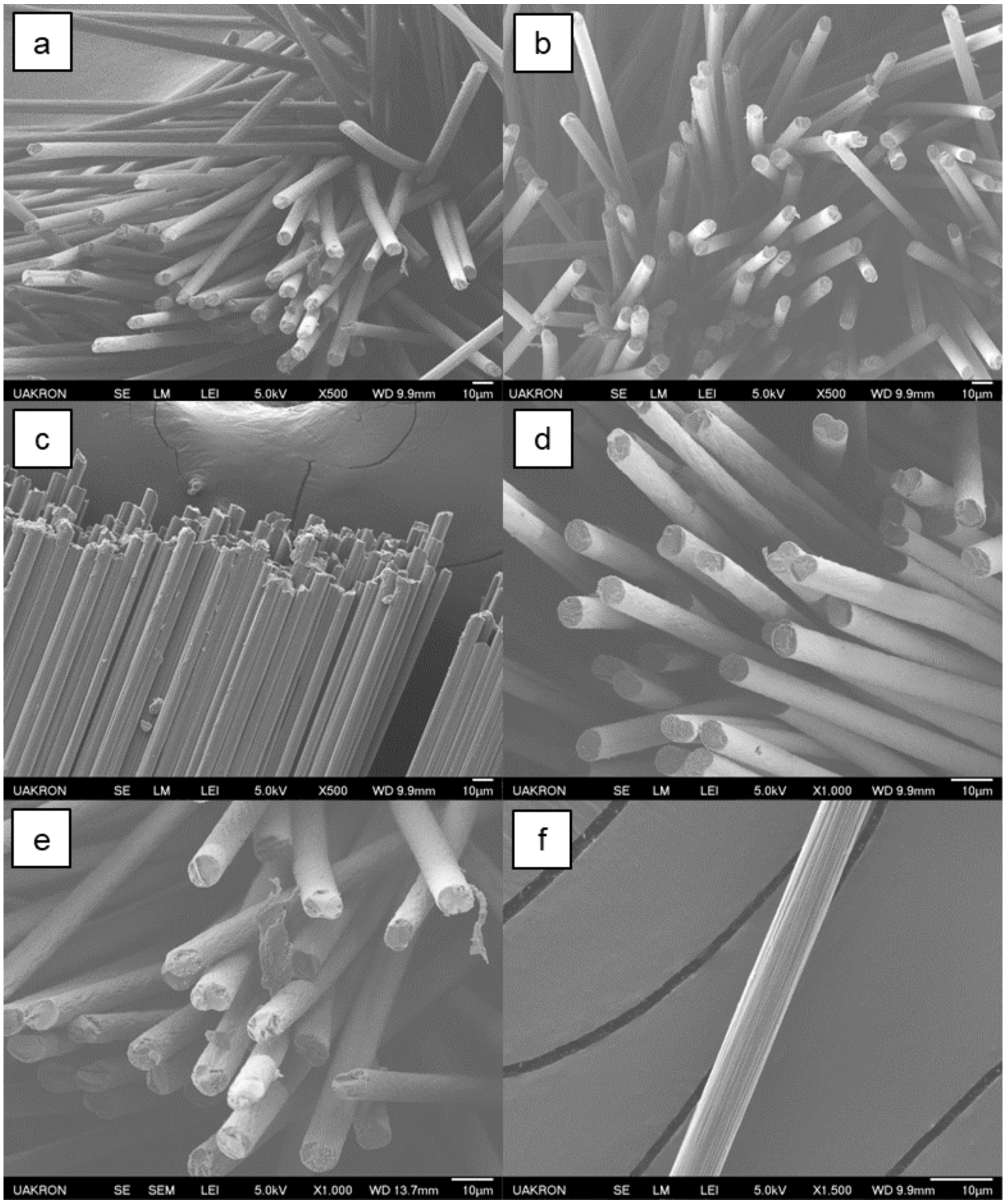

Fig. S91. SEM images of plain weave carbon fiber recovered from **1**/TREN composite via acetic acid digestion. (a–c) Bundles of recovered fibers demonstrating removal of matrix material between fibers. (d,e) Close-up of bundles of fibers. (f) Side profile close-up of an individual recovered fiber. Inset (a) reused in manuscript Fig 5.

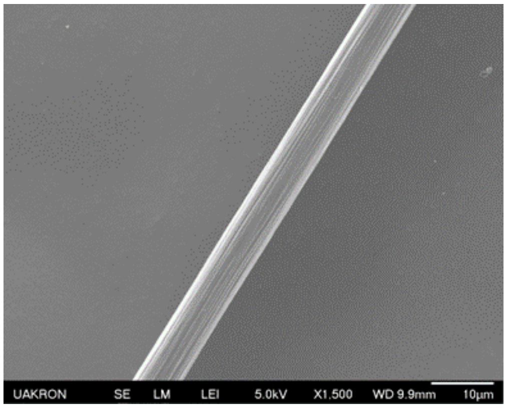

Fig. S92. SEM side profile image of an individual carbon fiber after TGA analysis.

Density Measurements

Table S5. Density measurements of the thermoset networks and CFRPs.

Density (g/cm^3)					
Trial	DGEBA/TREN	**1**/TREN	DGEBA/TREN CFRP	**1**/TREN CFRP	4500 Infusion CFRP
1	1.146	1.097	1.361	1.398	1.346
2	1.127	1.112	1.361	1.402	1.305
3	1.205	1.102	1.363	1.392	1.355
4	1.136	1.115	1.360	1.402	1.349
5	1.173	1.135	1.365	1.403	1.367
6	-	-	1.361	1.402	1.342
Mean	1.157	1.112	1.362	1.400	1.344
St.Dev.	0.028	0.013	0.001	0.004	0.019

Tiglamide formation vs reaction temperature

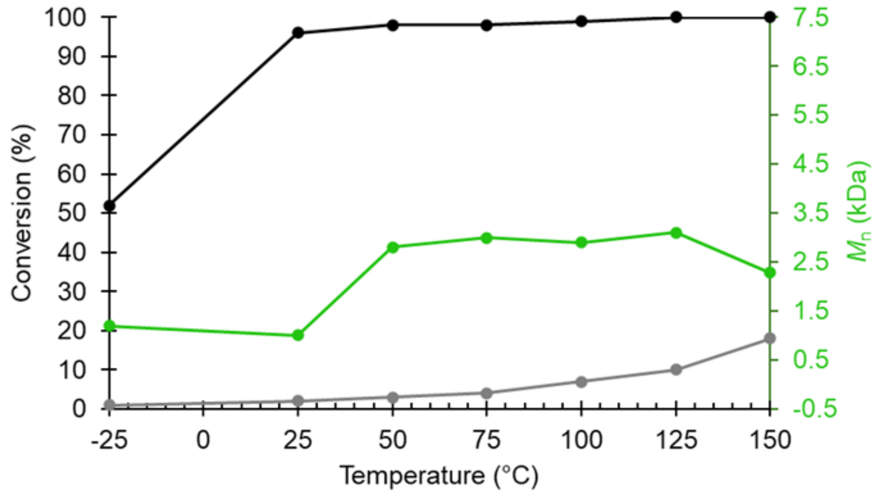

Fig. S93. Consumption of Lactone **1**, formation of tiglamide, and molecular weights of poly(**1**-Hexanediamine) for 24 hours, neat polymerization conditions. Data acquired by ^{1}H NMR (DMSO, 500 MHz).

Recovery and recycling spectra

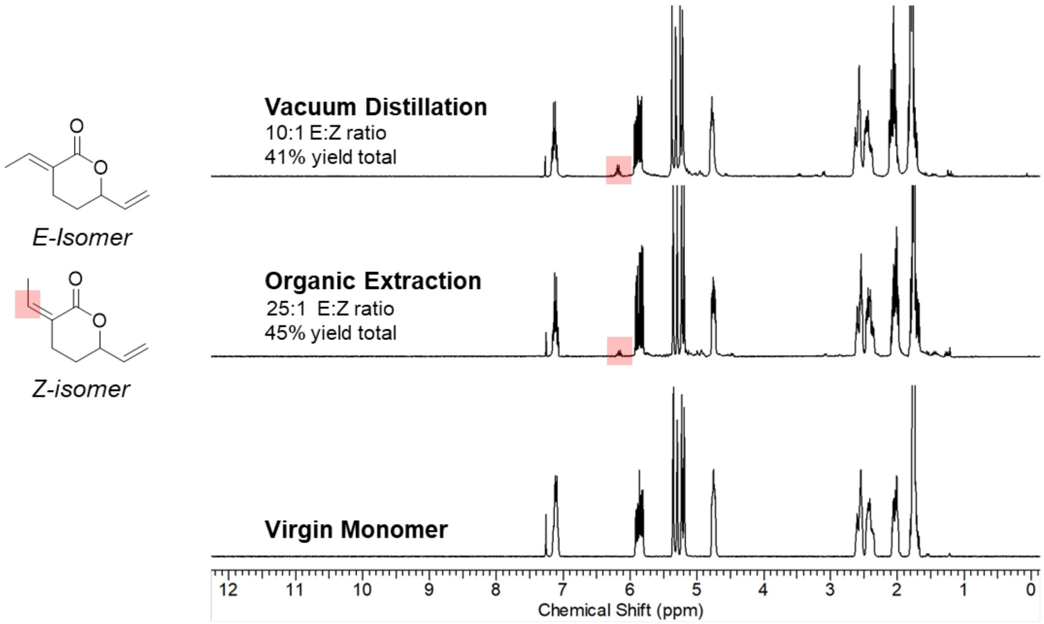

Fig. S94. ^{1}H NMR ($CDCl_3$, 500 MHz) of Lactone **1** recovered by vacuum distillation (top) and organic extraction using a 20:80 ethyl acetate:hexanes organic phase (middle) following digestion. The beta proton of the *Z*-isomer of Lactone **1** is highlighted in red and quantified.

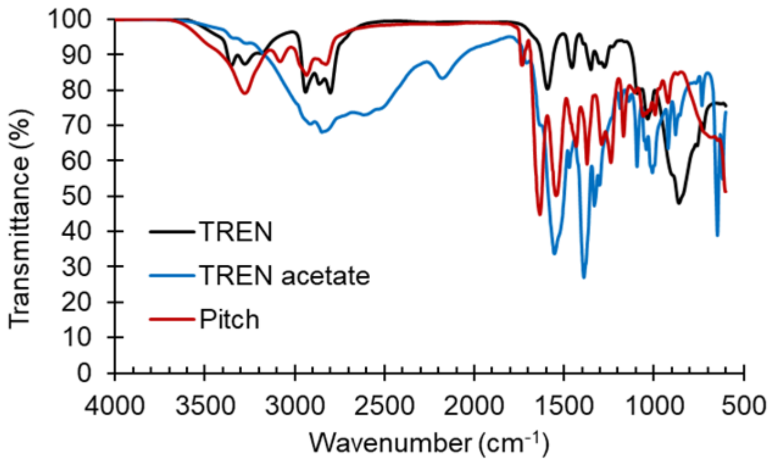

A)

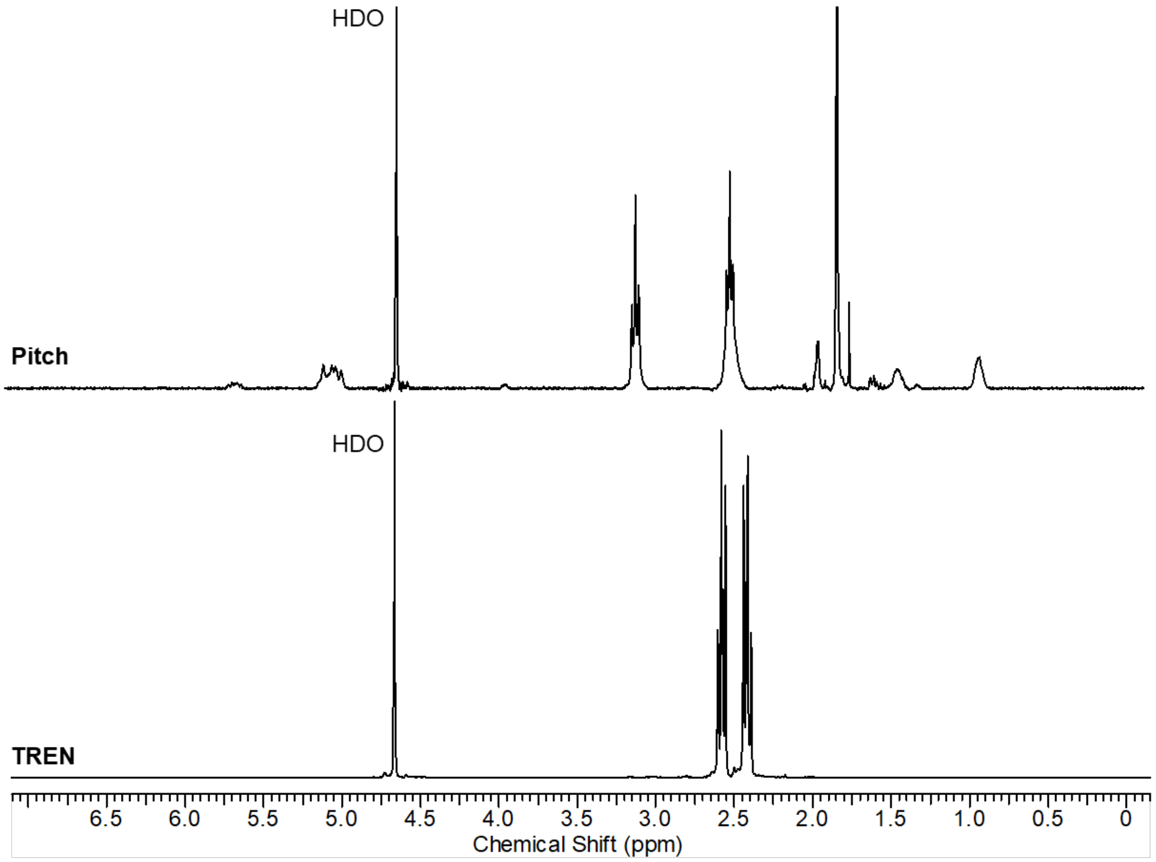

B)

Fig. S95. A) FTIR Comparing the undistilled pitch from vacuum distillation (red) with virgin TREN (black) and the ammonium acetate salt from mixing TREN + AcOH (blue). B) ^{1}H NMR (D_2O, 300 MHz) comparing TREN and the pitch following vacuum distillation of the digested media containing tris-acetylated TREN.

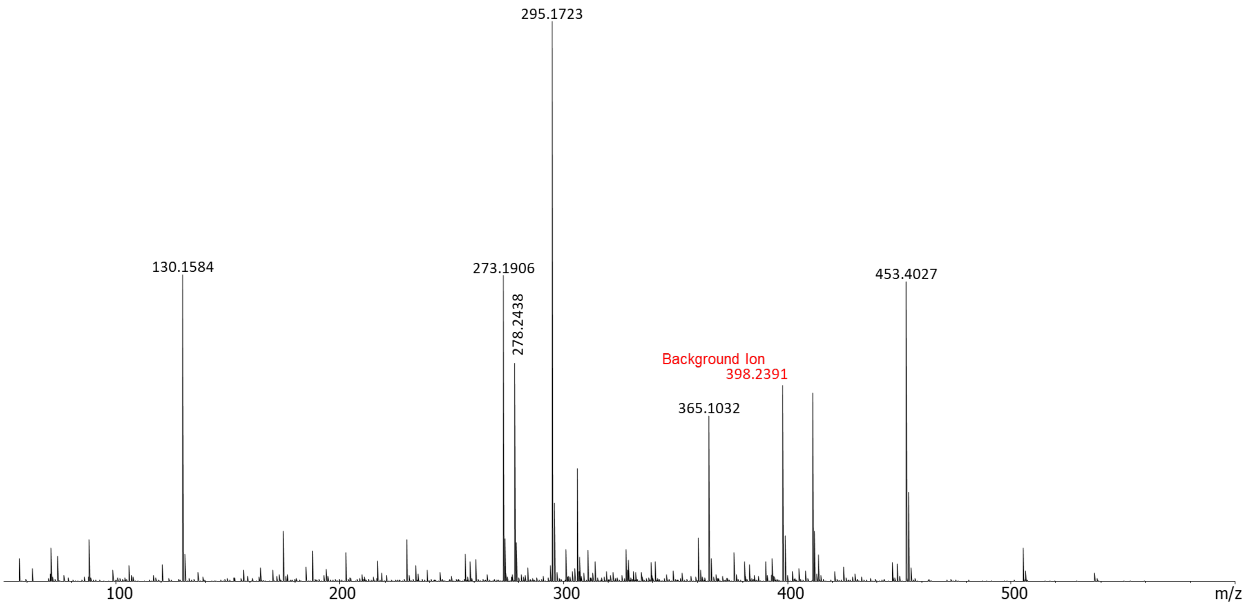

Fig. S96. ESI-MS of the pitch following vacuum distillation indicating tris-acetylated TREN+H (m/z = 273.1927) and tris-acetylated TREN+Na (m/z = 295.1746).

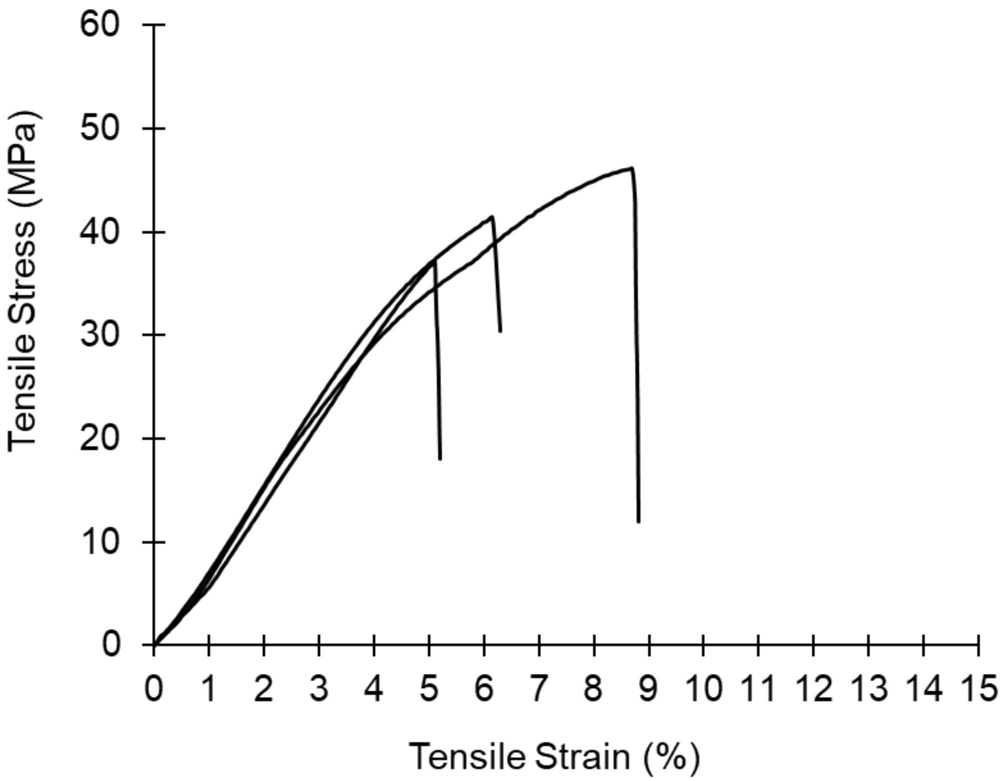

Fig. S97. Uniaxial tensile testing of recycled **1** / recycled TREN matrix. Performed in accordance with ASTM D638.(*54*)

Dynamic mechanical analysis

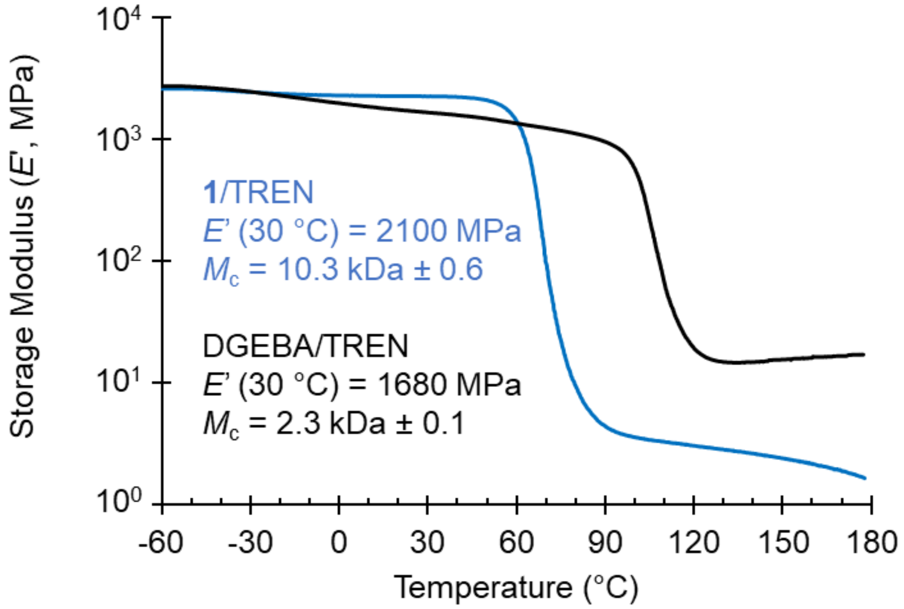

Fig. S98. Dynamic mechanical analysis performed using a TA Q800 with a gas cooling accessory supplied with LN_2. Temperature ramp conducted at 3°C/min beginning with a 5 min soak time at a starting temperature of -60°C and final temperature of 180 °C. Procedure used a frequency of 1 Hz and amplitude of 20 μm.

Recycled CFRP characterization

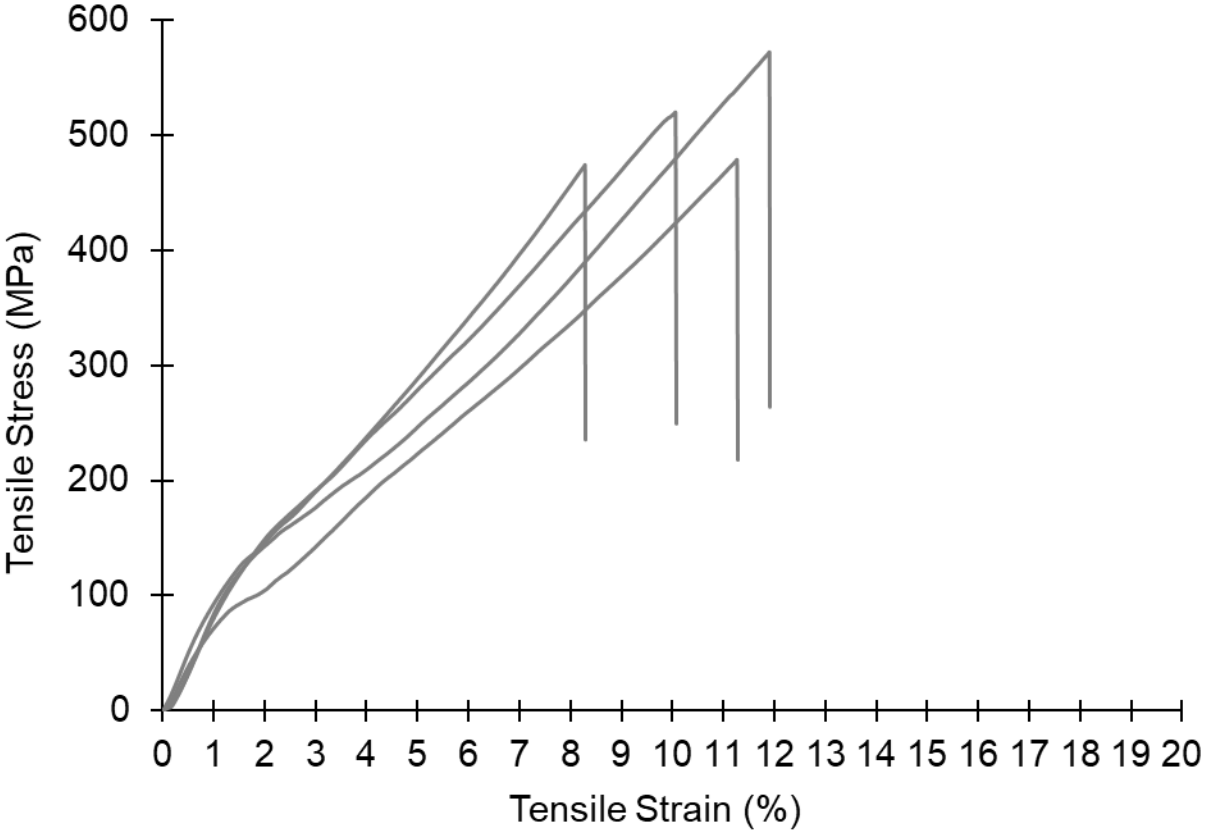

Fig. S99. Uniaxial tensile testing of 4500 Infusion 4-ply composite. Performed in accordance with ASTM D3039.(*55*)

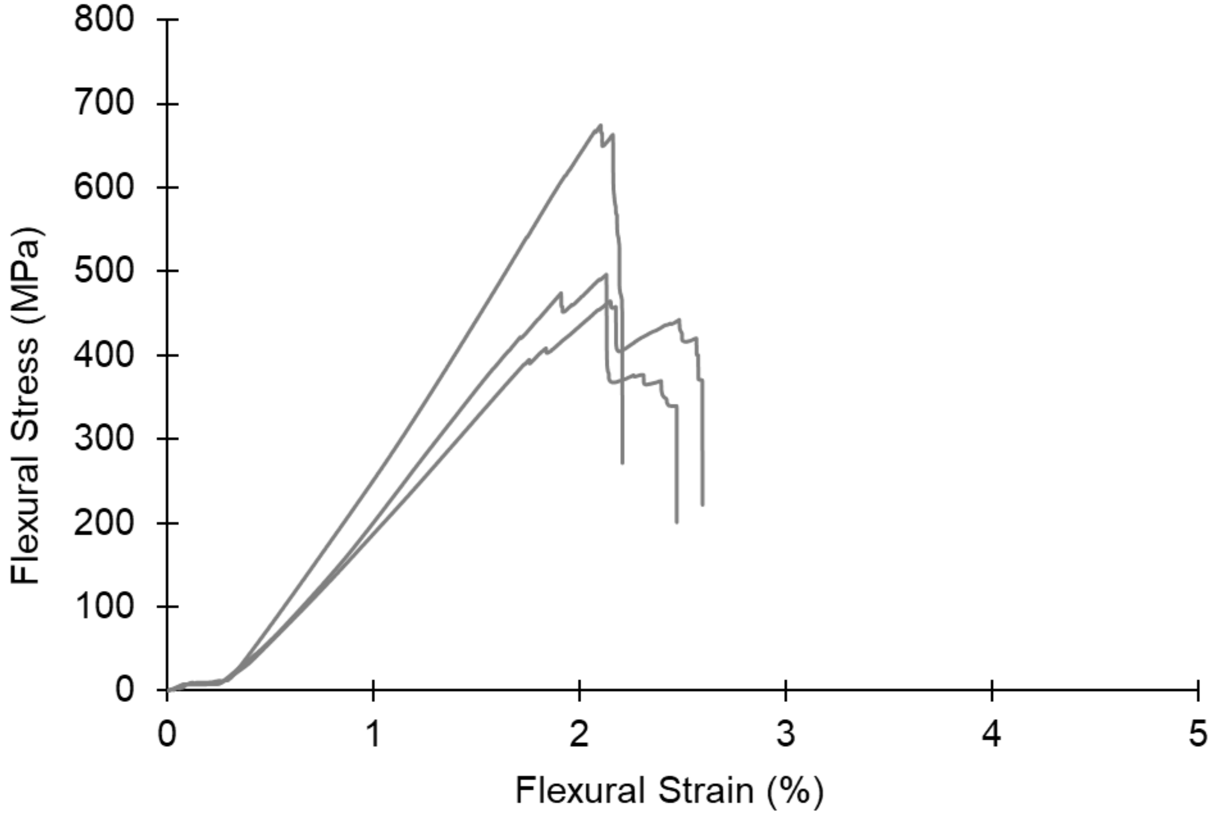

Fig. S100. Flexural testing of 4500 Infusion 4-ply composite. Performed in accordance with ASTM D790.(*56*)

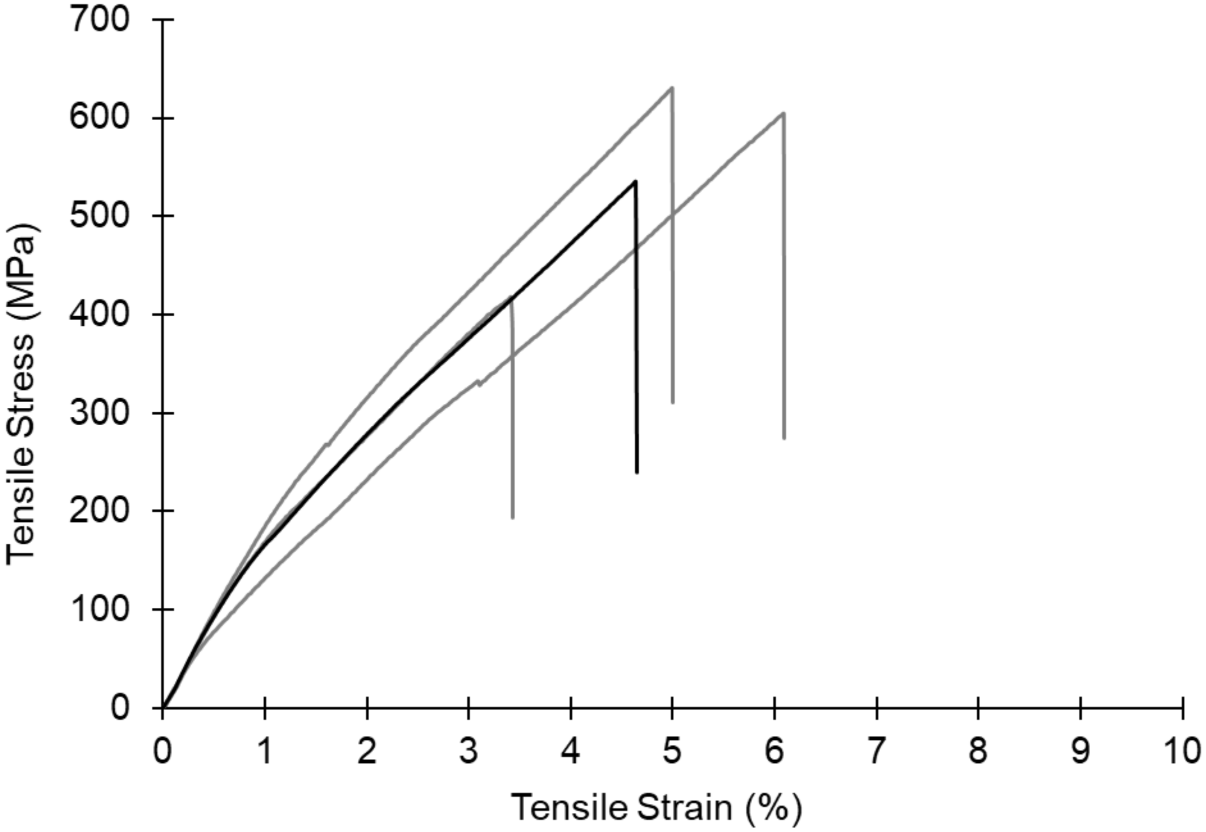

Fig. S101. Uniaxial tensile testing of Recycled **1**/TREN 4-ply composite. Performed in accordance with ASTM D3039.(*55*)

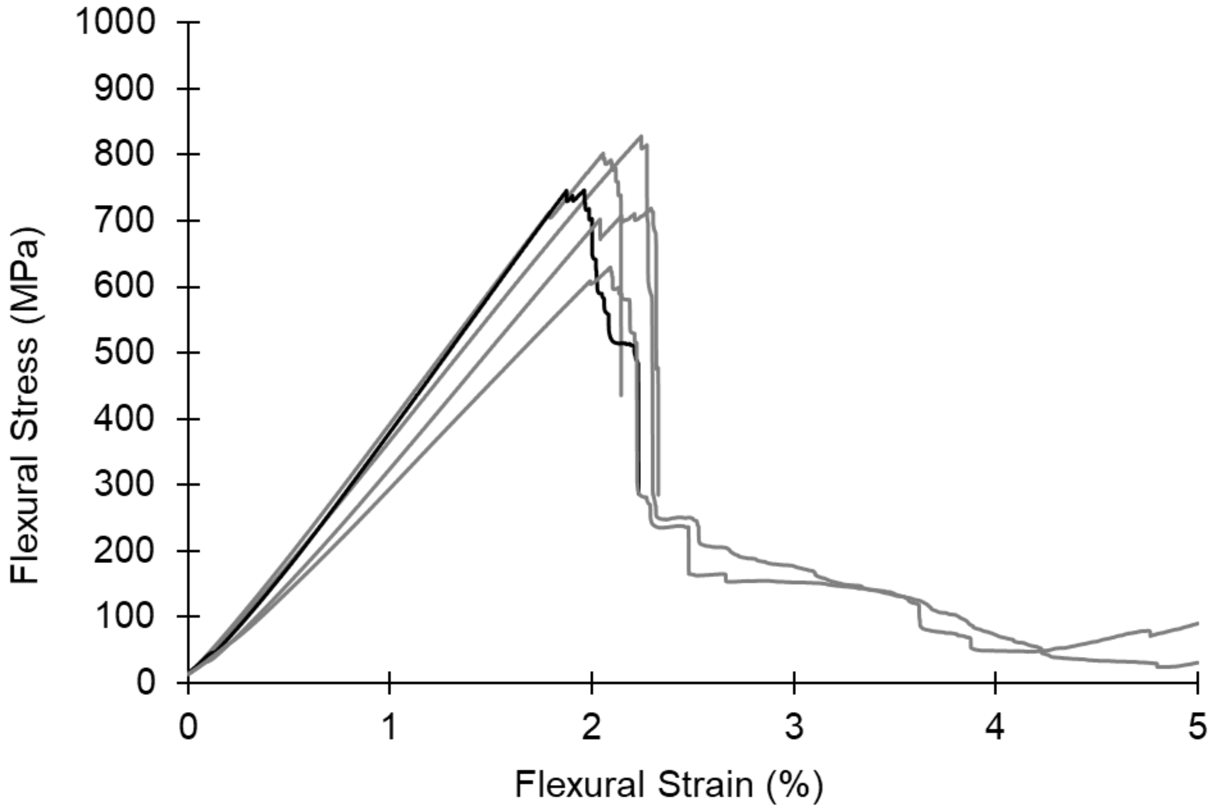

Fig. S102. Flexural testing of Recycled **1**/TREN 4-ply composite. Performed in accordance with ASTM D790.(*56*)

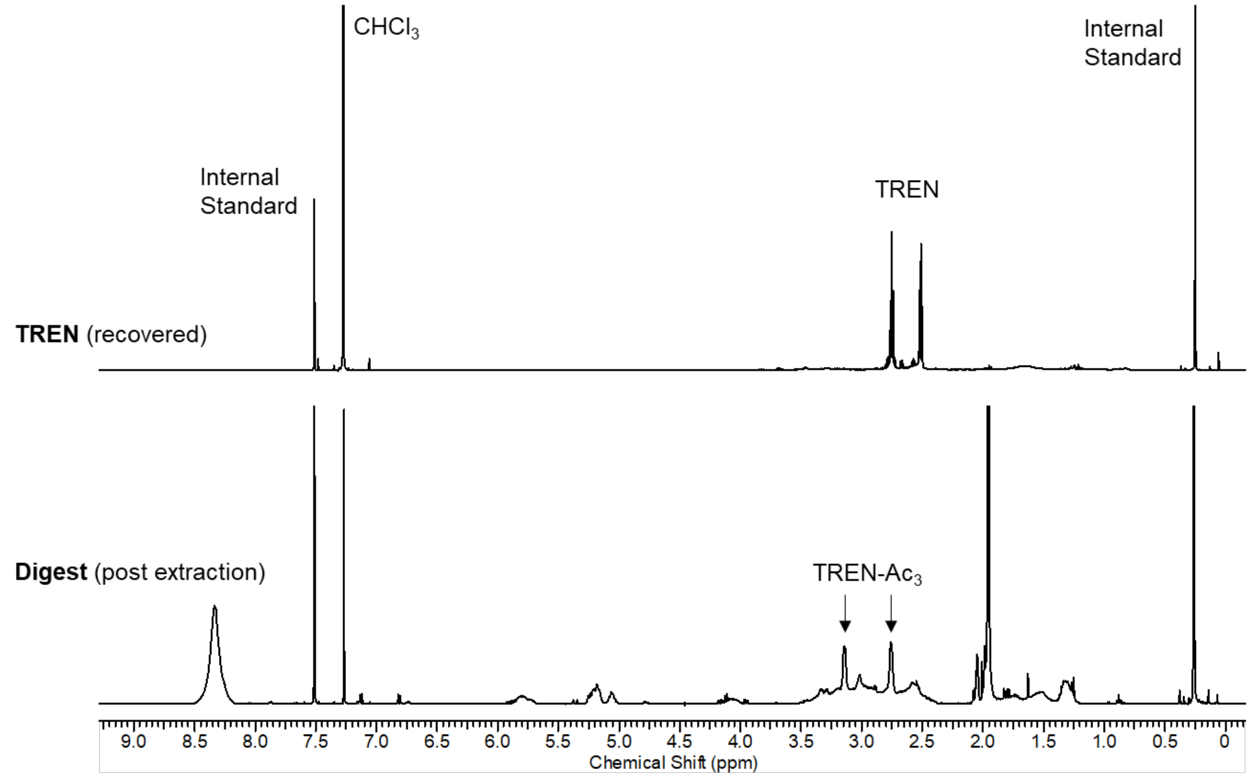

Fig. S103. ^{1}H NMR ($CDCl_3$, 500 MHz) of digested material dried from the aqueous layer of the organic extraction, showing the presence of acetylated TREN. Overlayed for comparison is the TREN recovered by hydrolysis of TREN-Ac_3 in KOH at 150°C.

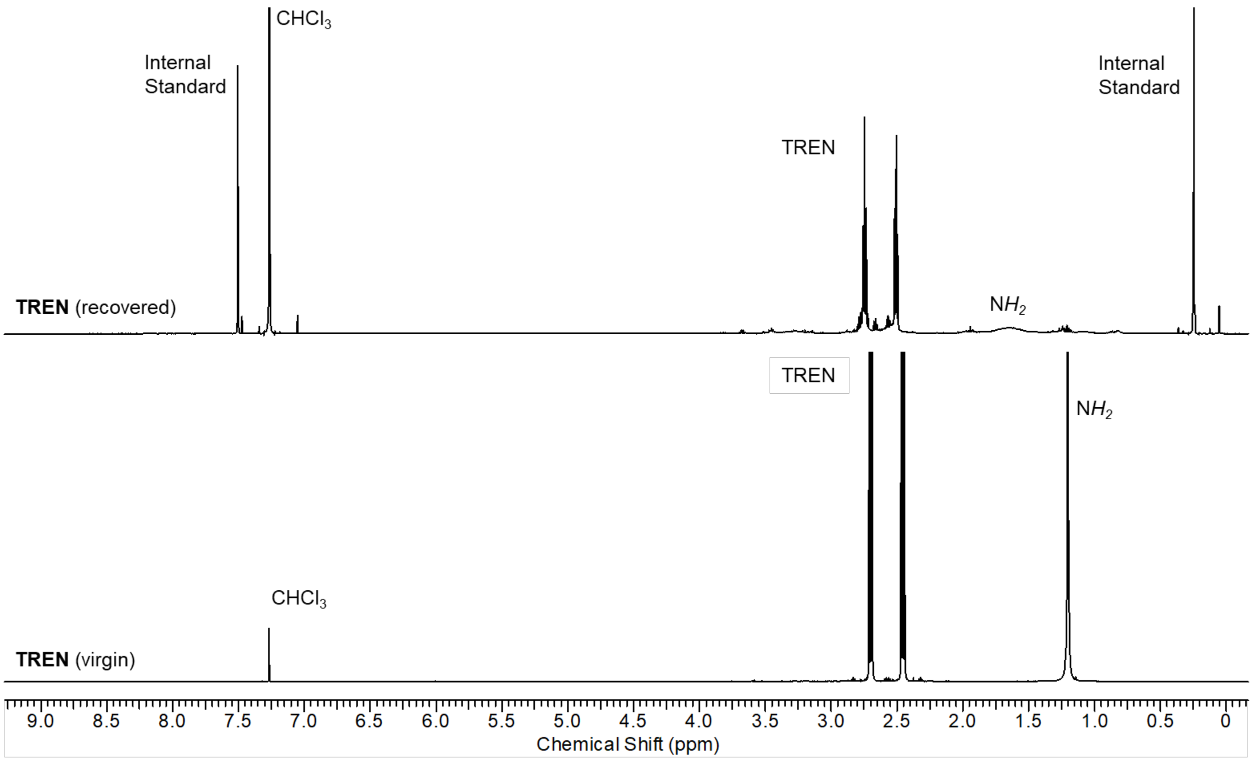

Fig. S104. ^{1}H NMR ($CDCl_3$, 500 MHz) comparing virgin TREN and TREN recovered by hydrolysis of the acetylated adduct formed during matrix digestion.

Supplementary Movie 1

Supplementary Movie 1. The digestion of a single-ply CFRP panel composed of **1**/TREN in acetic acid at 100°C.

REFERENCES

1. M. Bachmann, C. Zibunas, J. Hartmann, V. Tulus, S. Suh, G. Guillén-Gosálbez, A. Bardow, Towards circular plastics within planetary boundaries. *Nat. Sustain.* **6**, 599–610 (2023).

2. B. Winter, R. Meys, A. Sternberg, A. Bardow, Sugar-to-what? An environmental merit order curve for biobased chemicals and plastics. *ACS Sustain. Chem. Eng.* **10**, 15648–15659 (2022).

3. C. Liang, U. R. Gracida-Alvarez, T. R. Hawkins, J. B. Dunn, Life-cycle assessment of biochemicals with clear near-term market potential. *ACS Sustain. Chem. Eng.* **11**, 2773–2783 (2023).

4. M. Aresta, A. Dibenedetto, A. Angelini, Catalysis for the valorization of exhaust carbon: From CO_2 to chemicals, materials, and fuels. Technological use of CO_2. *Chem. Rev.* **114**, 1709–1742 (2014).

5. D. Cespi, F. Passarini, I. Vassura, F. Cavani, Butadiene from biomass, a life cycle perspective to address sustainability in the chemical industry. *Green Chem.* **18**, 1625–1638 (2016).

6. Y. Sasaki, Y. Inoue, H. Hashimoto, Reaction of carbon dioxide with butadiene catalysed by palladium complexes. Synthesis of 2-ethylidenehept-5-en-4-olide. *J. Chem. Soc. Chem. Commun.*, 605–606 (1976).

7. J. A. Daniels, Telomerization of butadiene and carbon dioxide, European Patent Application DE3163907D1 (1984).

8. A. Behr, Method for producing lactones, European Patent Application DE3317013A1 (1983).

9. A. Behr, M. Becker, The telomerisation of 1,3-butadiene and carbon dioxide: Process development and optimisation in a continuous miniplant. *Dalton Trans.*, 4607–4613 (2006).

10. T. A. Faßbach, A. J. Vorholt, W. Leitner, The telomerization of 1,3-dienes—A reaction grows up. *ChemCatChem* **11**, 1153–1166 (2019).

11. S. Tang, B.-L. Lin, I. Tonks, J. M. Eagan, X. Ni, K. Nozaki, Sustainable copolymer synthesis from carbon dioxide and butadiene. *Chem. Rev.* **124**, 3590–3607 (2024).

12. J. M. Eagan, The divergent reactivity of lactones derived from butadiene and carbon dioxide in macromolecular synthesis. *Macromol. Rapid Commun.* **44**, e2200348 (2022).

13. S. Tang, Y. Zhao, K. Nozaki, Accessing divergent main-chain-functionalized polyethylenes via copolymerization of ethylene with a CO_2/butadiene-derived lactone. *J. Am. Chem. Soc.* **143**, 17953–17957 (2021).

14. R. Nakano, S. Ito, K. Nozaki, Copolymerization of carbon dioxide and butadiene via a lactone intermediate. *Nat. Chem.* **6**, 325–331 (2014).

15. R. M. Rapagnani, R. J. Dunscomb, A. A. Fresh, I. A. Tonks, Tunable and recyclable polyesters from CO_2 and butadiene. *Nat. Chem.* **14**, 877–883 (2022).

16. L. D. Garcia Espinosa, K. Williams-Pavlantos, K. M. Turney, C. Wesdemiotis, J. M. Eagan, Degradable polymer structures from carbon dioxide and butadiene. *ACS Macro Lett.* **10**, 1254–1259 (2021).

17. K. Chen, Z. Zhu, T. Bai, Y. Mei, T. Shen, J. Ling, X. Ni, A topology-defined polyester elastomer from CO_2 and 1,3-butadiene: A one-pot-one-step "scrambling polymerizations" strategy. *Angew. Chem. Int. Ed. Engl.* **61**, e202213028 (2022).

18. Y. Lou, L. Xu, N. Gan, Y. Sun, B.-L. Lin, Chemically recyclable polyesters from CO_2, H_2, and 1,3-butadiene. *Int. J. Hydrogen Energ.* **3**, 100216 (2022).

19. J. Song, K. Chen, Y. Feng, X. Ni, J. Ling, One-pot orthogonal thiol-ene click polymerization and ring-opening grafting reaction of CO_2-based disubstituted δ-valerolactone. *J. Polym. Sci.* **60**, 2352–2361 (2022).

20. L. Chen, J. Ling, X. Ni, Z. Shen, Synthesis and properties of networks based on thiol-ene chemistry using a CO_2-based δ-lactone. *Macromol. Rapid Commun.* **39**, 1800395 (2018).

21. H. Dodiuk, *Handbook of Thermoset Plastics* (William Andrew, ed. 3, 2013).

22. "Bandwidth study on energy use and potential energy saving opportunities in the manufacturing of lightweight materials: Carbon fiber reinforced polymer composites" (Tech. Rep. DOE/EE-1662, US Department of Energy, 2017).

23. G. Lubin, *Handbook of Composites* (Springer Science and Business Media, 2013).

24. R. A. Witik, R. Teuscher, V. Michaud, C. Ludwig, J.-A. E. Månson, Carbon fibre reinforced composite waste: An environmental assessment of recycling, energy recovery and landfilling. *Compos. Part Appl. Sci. Manuf.* **49**, 89–99 (2013).

25. S. R. Naqvi, H. M. Prabhakara, E. A. Bramer, W. Dierkes, R. Akkerman, G. Brem, A critical review on recycling of end-of-life carbon fibre/glass fibre reinforced composites waste using pyrolysis towards a circular economy. *Resour. Conserv. Recycl.* **136**, 118–129 (2018).

26. T. Liu, L. Shao, B. Zhao, Y. Chang, J. Zhang, Progress in chemical recycling of carbon fiber reinforced epoxy composites. *Macromol. Rapid Commun.* **43**, e2200538 (2022).

27. B. M. Alameda, M. S. Kumler, J. S. Murphy, J. S. Aguinaga, D. L. Patton, Cyclic ketal bridged bisepoxides: Enabling the design of degradable epoxy-amine thermosets for carbon fiber composite applications. *RSC Appl. Polym.* **1**, 254–265 (2023).

28. W. Ballout, N. Sallem-Idrissi, M. Sclavons, C. Doneux, C. Bailly, T. Pardoen, P. Van Velthem, High performance recycled CFRP composites based on reused carbon fabrics through sustainable mild solvolysis route. *Sci. Rep.* **12**, 5928 (2022).

29. M. A. Rahman, M. S. Karunarathna, C. C. Bowland, G. Yang, C. Gainaru, B. Li, S. Kim, V. Chawla, N. Ghezawi, H. M. Meyer, A. K. Naskar, D. Penumadu, A. P. Sokolov, T. Saito, Tough and recyclable carbon-fiber composites with exceptional interfacial adhesion via a tailored vitrimer-fiber interface. *Cell Rep. Phys. Sci.* **4**, 101695 (2023).

30. C. Wang, R. E. Murray, G. T. Beckham, S. Mauger, N. A. Rorrer, Bioderived recyclable epoxy-anhydride thermosetting polymers and resins, US Patent US2022/0106442 A1 (2024).

31. P. R. Christensen, A. M. Scheuermann, K. E. Loeffler, B. A. Helms, Closed-loop recycling of plastics enabled by dynamic covalent diketoenamine bonds. *Nat. Chem.* **11**, 442–448 (2019).

32. C. Wang, A. Singh, E. G. Rognerud, R. Murray, G. M. Musgrave, M. Skala, P. Murdy, J. S. DesVeaux, S. R. Nicholson, K. Harris, R. Canty, F. Mohr, A. J. Shapiro, D. Barnes, R. Beach, R. D. Allen, G. T. Beckham, N. A. Rorrer, Synthesis, characterization, and recycling of bio-derivable polyester covalently adaptable networks for industrial composite applications. *Matter* **7**, 550–568 (2024).

33. N. J. Van Zee, R. Nicolaÿ, Vitrimers: Permanently crosslinked polymers with dynamic network topology. *Prog. Polym. Sci.* **104**, 101233 (2020).

34. H. Si, L. Zhou, Y. Wu, L. Song, M. Kang, X. Zhao, M. Chen, Rapidly reprocessable, degradable epoxy vitrimer and recyclable carbon fiber reinforced thermoset composites relied on high contents of exchangeable aromatic disulfide crosslinks. *Compos. Part B Eng.* **199**, 108278 (2020).

35. X. Wu, P. Hartmann, D. Berne, M. De Bruyn, F. Cuminet, Z. Wang, J. M. Zechner, A. D. Boese, V. Placet, S. Caillol, K. Barta, Closed-loop recyclability of a biomass-derived epoxy-amine thermoset by methanolysis. *Science* **384**, eadj9989 (2024).

36. D. J. Fortman, J. P. Brutman, G. X. De Hoe, R. L. Snyder, W. R. Dichtel, M. A. Hillmyer, Approaches to sustainable and continually recyclable cross-linked polymers. *ACS Sustain. Chem. Eng.* **6**, 11145–11159 (2018).

37. R. Auvergne, S. Caillol, G. David, B. Boutevin, J.-P. Pascault, Biobased thermosetting epoxy: Present and future. *Chem. Rev.* **114**, 1082–1115 (2014).

38. R. W. Clarke, E. G. Rognerud, A. Puente-Urbina, D. Barnes, P. Murdy, M. L. McGraw, J. M. Newkirk, R. Beach, J. A. Wrubel, L. J. Hamernik, K. A. Chism, A. L. Baer, G. T. Beckham, R. E. Murray, N. A. Rorrer, Manufacture and testing of biomass-derivable thermosets for wind blade recycling. *Science* **385**, 854–860 (2024).

39. B. Grignard, S. Gennen, C. Jérôme, A. W. Kleij, C. Detrembleur, Advances in the use of CO_2 as a renewable feedstock for the synthesis of polymers. *Chem. Soc. Rev.* **48**, 4466–4514 (2019).

40. G. Seychal, P. Nickmilder, V. Lemaur, C. Ocando, B. Grignard, P. Leclère, C. Detrembleur, R. Lazzaroni, H. Sardon, N. Aranburu, J.-M. Raquez, A novel approach to design structural natural fiber composites from sustainable CO_2-derived polyhydroxyurethane thermosets with outstanding properties and circular features. *J. Compos. Part A.* **185**, 108311 (2024).

41. T. Habets, G. Seychal, M. Caliari, J.-M. Raquez, H. Sardon, B. Grignard, C. Detrembleur, Covalent adaptable networks through dynamic N,S-acetal chemistry: Toward recyclable CO_2-based thermosets. *J. Am. Chem. Soc.* **145**, 25450–25462 (2023).

42. S. Moon, K. Masada, K. Nozaki, Reversible polymer-chain modification: Ring-opening and closing of polylactone. *J. Am. Chem. Soc.* **141**, 10938–10942 (2019).

43. A. Behr, G. Henze, L. Johnen, S. Reyer, Selective catalytic formation of unsaturated amino acids from petrochemicals and carbon dioxide—Application of high-throughput catalyst screening. *J. Mol. Catal. Chem.* **287**, 95–101 (2008).

44. A. Behr, G. Henze, Use of carbon dioxide in chemical syntheses via a lactone intermediate. *Green Chem.* **13**, 25–39 (2011).

45. G. Henze, "Wertprodukte aus Butdien, Kohlendioxid und weiteren Basischemikalien," thesis, TU Dortmund (2008).

46. D. A. Tomalia, H. Baker, J. Dewald, M. Hall, G. Kallos, S. Martin, J. Roeck, J. Ryder, P. Smith, A new class of polymers: Starburst-dendritic macromolecules. *Polym. J.* **17**, 117–132 (1985).

47. Z. Zhang, T. Shen, K. Chen, J. Zeng, Y. Mei, J. Ling, X. Ni, Polyester platform with high refractive indices and closed-loop recyclability from CO_2, 1,3-butadiene, and thiols. *ACS Macro Lett.* **13**, 741–746 (2024).

48. G. B. Desmet, D. R. D'hooge, P. S. Omurtag, P. Espeel, G. B. Marin, F. E. Du Prez, M.-F. Reyniers, Quantitative first-principles kinetic modeling of the aza-Michael addition to acrylates in polar aprotic solvents. *J. Org. Chem.* **81**, 12291–12302 (2016).

49. S. Ilieva, B. Galabov, D. G. Musaev, K. Morokuma, H. F. Schaefer, Computational study of the aminolysis of esters. The reaction of methylformate with ammonia. *J. Org. Chem.* **68**, 1496–1502 (2003).

50. K. Turney, A. Mokarizadeh, M. Tsige, J. Eagan. Carbonization of butadiene enables recyclable carbon fiber polymer composites. ChemRxiv (2025). https://doi.org/10.26434/chemrxiv-2025-7kfc4.

51. A. Behr, K.-D. Juszak, Palladium-catalyzed reaction of butadiene and carbon dioxide. *J. Organomet. Chem.* **255**, 263–268 (1983).

52. M. Sharif, R. Jackstell, S. Dastgir, B. Al-Shihi, M. Beller, Efficient and selective palladium-catalyzed telomerization of 1,3-butadiene with carbon dioxide. *ChemCatChem* **9**, 542–546 (2017).

53. J. M. Balbino, J. Dupont, J. C. Bayón, Telomerization of 1,3-butadiene with carbon dioxide: a highly efficient process for δ-lactone generation. *ChemCatChem* **10**, 206–210 (2018).

54. "Standard Test Method for Tensile Properties of Plastics; ASTM D638" (ASTM Standard, ASTM International, 2017).

55. "Standard Test Method for Tensile Properties of Polymer Matrix Composite Materials; ASTM D3039" (ASTM Standard, ASTM International, 2017).

56. "Standard Test Methods for Flexural Properties of Unreinforced and Reinforced Plastics and Electrical Insulating Materials; ASTM D790-17" (ASTM Standard, ASTM International, 2017).
